# Revisiting methotrexate and phototrexate Zinc15 library-based derivatives using deep learning *in-silico* drug design approach

**DOI:** 10.3389/fchem.2024.1380266

**Published:** 2024-03-21

**Authors:** Farhan Siddique, Ahmar Anwaar, Maryam Bashir, Sumaira Nadeem, Ravi Rawat, Volkan Eyupoglu, Samina Afzal, Mehvish Bibi, Yousef A. Bin Jardan, Mohammed Bourhia

**Affiliations:** ^1^ School of Pharmaceutical Science and Technology, Tianjin University, Tianjin, China; ^2^ Department of Pharmaceutical Chemistry, Faculty of Pharmacy, Bahauddin Zakariya University, Multan, Pakistan; ^3^ Faculty of Pharmacy, Bahauddin Zakariya University, Multan, Pakistan; ^4^ Southern Punjab Institute of Health Sciences, Multan, Pakistan; ^5^ Department of Pharmacy, The Women University, Multan, Pakistan; ^6^ School of Health Sciences & Technology, UPES University, Dehradun, India; ^7^ Department of Chemistry, Cankırı Karatekin University, Cankırı, Türkiye; ^8^ Department of Pharmaceutics, College of Pharmacy, King Saud University, Riyadh, Saudi Arabia; ^9^ Laboratory of Biotechnology and Natural Resources Valorization, Faculty of Sciences, Ibn Zohr University, Agadir, Morocco

**Keywords:** deep learning, QSAR, virtual screening, density function theory, molecular dynamics, dihydrofolate reductase

## Abstract

**Introduction:** Cancer is the second most prevalent cause of mortality in the world, despite the availability of several medications for cancer treatment. Therefore, the cancer research community emphasized on computational techniques to speed up the discovery of novel anticancer drugs.

**Methods**: In the current study, QSAR-based virtual screening was performed on the Zinc15 compound library (271 derivatives of methotrexate (MTX) and phototrexate (PTX)) to predict their inhibitory activity against dihydrofolate reductase (DHFR), a potential anticancer drug target. The deep learning-based ADMET parameters were employed to generate a 2D QSAR model using the multiple linear regression (MPL) methods with Leave-one-out cross-validated (LOO-CV) Q^2^ and correlation coefficient R^2^ values as high as 0.77 and 0.81, respectively.

**Results:** From the QSAR model and virtual screening analysis, the top hits (**09, 27, 41, 68, 74, 85, 99, 180**) exhibited pIC_50_ ranging from 5.85 to 7.20 with a minimum binding score of -11.6 to -11.0 kcal/mol and were subjected to further investigation. The ADMET attributes using the message-passing neural network (MPNN) model demonstrated the potential of selected hits as an oral medication based on lipophilic profile Log P (0.19-2.69) and bioavailability (76.30% to 78.46%). The clinical toxicity score was 31.24% to 35.30%, with the least toxicity score (8.30%) observed with compound **180**. The DFT calculations were carried out to determine the stability, physicochemical parameters and chemical reactivity of selected compounds. The docking results were further validated by 100 ns molecular dynamic simulation analysis.

**Conclusion:** The promising lead compounds found endorsed compared to standard reference drugs MTX and PTX that are best for anticancer activity and can lead to novel therapies after experimental validations. Furthermore, it is suggested to unveil the inhibitory potential of identified hits via in-vitro and in-vivo approaches.

## Introduction

Artificial neural networks employed to analyze complex biological data and accurate predictions have significantly revolutionized the canvas of drug discovery (Siddiq, 2022). This transformation can be attributed to the emergence of deep learning (DL), which is a subfield within machine learning. DL models can be employed in drug discovery to target receptors (Sánchez-Linares et al., 2012). This can be achieved by training these models using comprehensive datasets consisting of molecular structures, biological activities, and structural characteristics. This methodology facilitates the forecast of the binding affinity and potency of probable drug candidates (Zhang et al., 2017). Models can efficiently capture intricate relationships between chemical structures and biological activities (Huang et al., 2020a), thus aiding researchers in screening and prioritizing compounds more likely to interact with biological targets (Sutherland et al., 2003).

DL models can automatically learn relevant features and patterns from data, making them more flexible and potentially more accurate for predicting ligand-target interactions and ADME (Absorption, distribution, metabolism, and excretion) properties. The traditional *in-vitro* and *in-vivo* methods are no doubt valuable to estimate human pharmacokinetics (PK) parameters; however, it is usually impossible to conduct these complex and expensive experiments on a large number of investigated drug moieties. The integration of Zinc15 resembling the library of MTX and PTX with artificial intelligence (AI)-based approaches QSAR and DL based ADME lead to qualitative and quantitative prediction of human PK of a drug of interest. However, predicting drug response with these approaches is challenging, partially because of the adaptation of algorithms and limitations related to experimental data (Aziz et al., 2022a). In this study, an evaluation of ADMET properties through *in silico* methods was conducted by utilizing a message-passing neural network (MPNN). The utilization of MPNN models has been extensive in predicting diverse molecular characteristics such as blood-brain barrier permeability, human intestinal absorption, and solubility trends (Tang et al., 2023). The ADMET properties of the identified hits were predicted by employing MPNN, yielding significant insights into their pharmacokinetic profiles (Fralish et al., 2023). The ADMET data of MTX has been predicted earlier via admetSAR database (Aher et al., 2020), by TOPKAT predictions (Rana et al., 2019).

DL and Structure-Based Virtual Screening (SBVS) techniques therefore not only facilitate the screening of vast chemical libraries but also provide valuable insights into the intricate relationships between molecular structure and biological activity, particularly in identifying cancer inhibitors (Andricopulo et al., 2008; Lavecchia and Di Giovanni, 2013; Abdolmaleki and Ghasemi, 2017). DL models can examine the associations between molecular characteristics and biological activities (Lo et al., 2018), thereby providing significant insights for lead optimization and developing more potent and selective compounds (Kim et al., 2016; Huang et al., 2020b). Cancer is a profoundly debilitating condition affecting many individuals worldwide, leading to significant illness and death (Liamputtong and Suwankhong, 2015; Al-Jumaili et al., 2023a). Despite the advancements in treatment approaches, there remains a critical need for developing innovative cancer therapies (Kalaydina et al., 2018; Siddique et al., 2024). Strategically targeting specific molecular pathways associated with cancer progression can significantly improve patient prognosis (Santarpia et al., 2012). Due to its contribution to the folate metabolic pathway, the DHFR enzyme has been renowned as a prominent target for cancer therapy (Nilsson et al., 2014) as it plays a crucial role in synthesizing DNA precursors. It catalyzes the conversion of dihydrofolate to tetrahydrofolate, a vital coenzyme involved in nucleotide biosynthesis (Nazki et al., 2014) and is considered to be a promising target for cancer treatment owing to its pivotal role in cellular proliferation and growth. The inhibition of DHFR leads to the disruption of nucleotide production, ultimately inhibiting DNA synthesis and subsequent suppression of cancer cell proliferation (Singh et al., 2018; Yang et al., 2021). MTX and PTX are widely recognized pharmaceutical agents for managing diverse neoplastic conditions (Matera et al., 2018). These agents are categorized as antifolates, and their mechanism of action involves the inhibition of DHFR (Matera et al., 2019). Nevertheless, these pharmaceuticals may frequently present constraints, such as the emergence of resistance and unfavorable reactions. Hence, it is necessary to re-investigate novel derivatives of MTX and PTX that can augment their therapeutic potency while mitigating their limitations (Huennekens, 1994).

The objective of this research was to combine the benefits of DL and SBVS techniques to identify the potential inhibitors that exhibit the potential to target DHFR for the treatment of cancer. Researchers can utilize DL models trained on diverse datasets of molecular structures and their corresponding activities to accurately predict compounds’ binding affinity and potency towards DHFR (Winkler, 2021; Aziz et al., 2022a). Subsequently, the application of SBVS was employed to identify potential lead compounds that demonstrate favorable interactions with the active site of DHFR (Ferreira et al., 2015; Staszak et al., 2022; Ferreira et al., 2023). The study aimed to investigate the potential of new top-hit compounds by revisiting and reevaluating MTX and PTX for treating DHFR-associated malignancies to previously reported motivation (Raimondi et al., 2019). To achieve this, we utilized advanced computational techniques such as DL virtual screening, QSAR analysis, Density functional theory (DFT) (Aziz et al., 2022b), molecular docking, ADMET studies, and MD simulations. Our work provides a fresh perspective on these well-known inhibitors, potentially revealing novel insights and applications that can contribute to the ongoing efforts to combat cancer.

## Methodology

### Computational studies

A compound library of 271 derivatives resembling MTX and PTX was employed in this study (also look at Supplementary Table S1). These compounds were specifically chosen as structural analogues of MTX and PTX, aiming to explore their potential as anticancer agents. The selection was based on their structural similarity to established anticancer drugs, focusing on compounds with analogous features and characteristics.

### Structure-Based Virtual Screening and molecular docking

**FIGURE 1:**
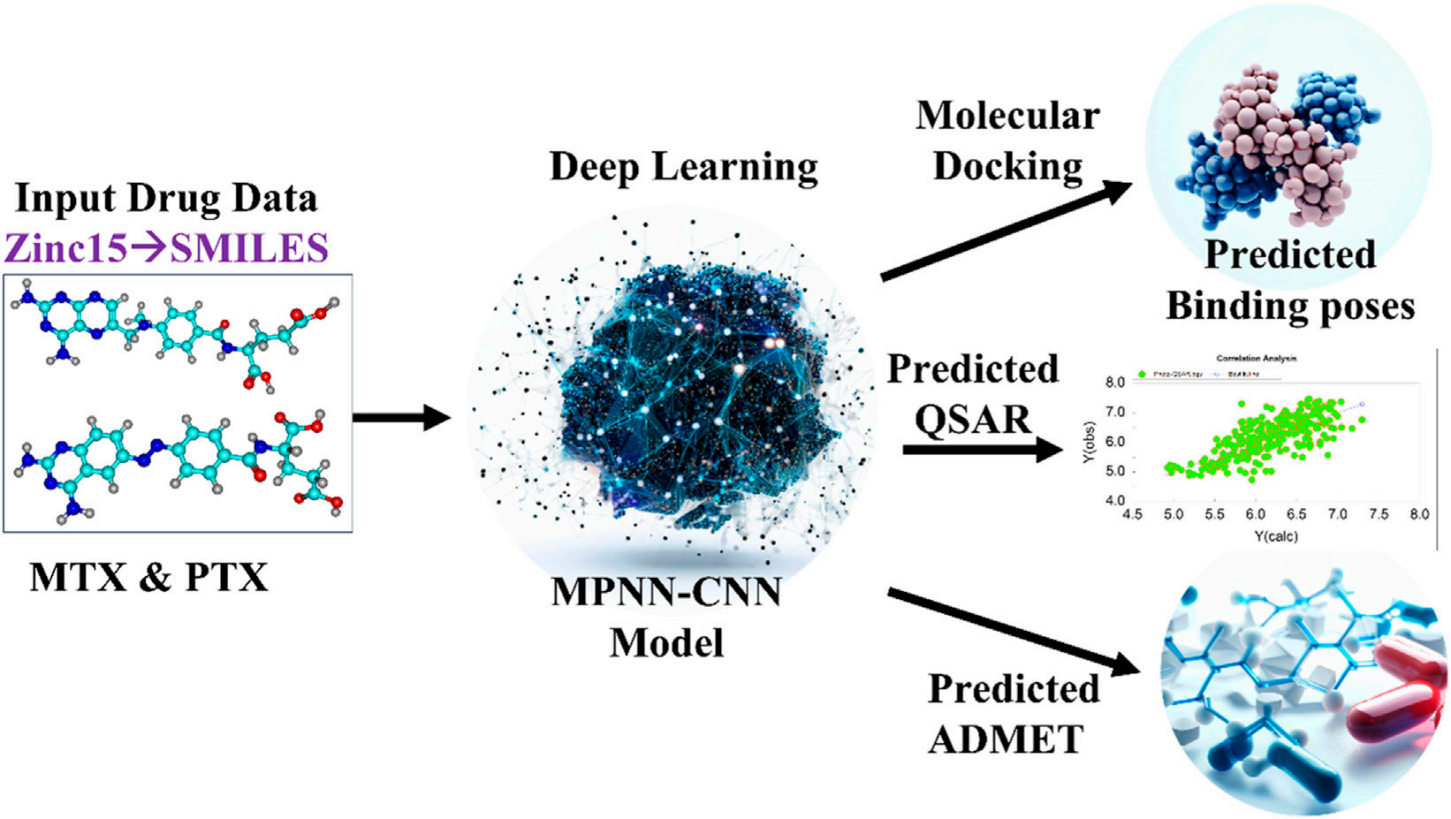
Scheme of Study for Implementation of DL Model. MTX and PTX images created by Chemcraft (Zhurko and Zhurko, 2005), Deep learning, predicted binding poses and ADMET images created with AI (magic studio module www.canva.com), predicted QSAR created with OriginLab (Deschenes and Vanden BoutUniversity of Texas, 2000).

The scheme of study with the implementation of the DL model is shown in Figure 1. The library combined contained 271 structural analogues (as shown in the Supplementary Table S2) of MTX and PTX. The compounds library was prepared using DFT-optimized geometries and converted to pdbqt format for virtual screening using Auto Dock Vina (Trott et al., 2010). The crystallographic structure of the protein of interest was obtained from the Protein Data Bank (PDB) (https://www.rcsb.org/), accessed on May 3^rd^, 2023. The target PDB identification code for the structure is 1U72 (Human Dihydrofolate Reductase Receptor). Subsequently, the macromolecules were prepared using MGL tools, involving the elimination of heteroatoms and water molecules, as well as the introduction of polar hydrogen atoms. The protein was assessed for any missing amino acid residues. Kollman’s charges were computed to neutralize the protein, ensuring its overall neutrality, while Gasteiger’s charges were calculated for the ligand, aligning with common practice in molecular simulations (Weiner et al., 1984; Dermawan et al., 2021). To conduct a virtual screening of the compound library using Auto Dock Vina, grid box size (*x* = 80, *y* = 80, *z* = 80) and dimensions (*x* = 31.10, *y* = 14.21 and *z* = −8.07) were adjusted for XYZ coordinates to cover the active binding pocket during the virtual screening using Auto Dock Vina. The process of virtual screening was conducted after the preparation of the specific protein, employing the script-based approach of Auto Dock Vina. The value for exhaustiveness was configured as 8, while the number of nodes was specified as 30. The virtual screening procedure was conducted twice to validate the precision of the docking outcomes.

The docking protocol was rigorously validated through a redocking procedure using the same algorithm and parameters employed for the investigated compounds. This validation method ensures the ability of the molecular modeling simulation to accurately replicate the ligand binding mode and residue-wise interaction patterns. Specifically, achieving a root mean square deviation (RMSD) value below 2 angstroms between the native and redocked poses confirms the reliability and biological significance of the adopted docking protocol (Katari et al., 2016). Following the conclusion of the virtual screening process, the resultant findings from the virtual screening module were subjected to analysis and docking scores of the drug candidates were compared to those of the standard drugs MTX and PTX.

### DL model

DL models were used to predict drug-target interactions (DTI) in the current work. The training data consists of a diverse dataset of drug compounds and their respective protein targets. This dataset was carefully curated from publicly available sources and annotated with high-quality labels to ensure data integrity. Including diverse compounds, targets, and proteins helps ensure the model’s predictions apply to various scenarios. The model architecture was based on encoder and decoder architectures, widely used in deep learning for sequence-to-sequence tasks. This choice of architecture was made due to its effectiveness in handling the complex relationships between drug compounds and protein sequences. The encoder processed the Simplified Molecular Input Line Entry System (SMILES) string of the drug compound, while the decoder handled the amino acid sequence of the target protein. Such architecture allows the model to capture intricate features and interactions between drugs and targets. For performance evaluation, a rigorous and well-established protocol was followed. The dataset was split into training, validation, and test sets to assess the model’s generalization ability. The model was trained using over 17 state-of-the-art deep learning techniques, validated in various studies for their efficacy in DTI prediction. Measure the model’s performance using metrics such as precision, recall, F1-score, and receiver operating characteristic (ROC) curves. This extensive performance evaluation approach ensures the model’s predictions are robust and reliable (Huang et al., 2020a; Aziz et al., 2022a; Imran et al., 2023). This study utilized the DeepPurpose architecture (http://deeppurpose.sunlab.org/), a machine-learning approach for predicting the interaction between ligands and target proteins. The pre-trained model, MPNN-CNN-Binding Data Base-IC_50_, which combines the Message Passing Neural Network (MPNN) with Convolutional Neural Network (CNN) techniques, was used to predict drug-target interactions (DTI) in terms of binding affinity (IC_50_) and predicted pIC_50_ (Huang et al., 2020a; Aziz et al., 2022a; Imran et al., 2023). This model incorporates advanced neural network approaches to enhance the accuracy of DTI predictions.

### DL based QSAR

The inherent ability of AI to revolutionize the drug discovery process has already been established by the uptake and frequent use of absorption, distribution, metabolism, excretion, and toxicity (ADMET) predictive tools, virtual screening, and quantitative structure-activity relationship (QSAR) modeling (Leelananda and Lindert, 2016). Development and use of *in silico* QSAR models to forecast drug activity, has become increasingly targeted in drug development and drug discovery over the near past (Ekins et al., 2007). Simple ML algorithms like multiple linear regression (MLR) exhibit efficient utilization for model development of relatively small data sets. QSAR modelling aims to construct a model with strong robustness and predictive capacity (Veerasamy, 2011; Hammoudan et al., 2023).

### DL based pIC_50_, binding affinities and ADMET

The FDA-approved drugs MTX and PTX have demonstrated efficacy in cancer treatment by effectively inhibiting the DHFR pathway (Yamashita et al., 2020). The binding affinity (IC_50_) and ADMET profile predominantly influence the drug’s effectiveness (El-Adl et al., 2021). Consequently, DL models have been utilized to forecast the IC_50_, pIC_50_, and ADMET characteristics of the most promising candidates obtained via virtual screening (Al-Jumaili et al., 2023b; Choudhary et al., 2023). This approach aims to facilitate a direct evaluation of the binding affinity of these candidates regarding the established standards of MTX and PTX. Applying *in silico* methods for predicting binding affinity and ADMET characteristics presents a promising alternative to experimental approaches (Brogi, 2020). In the present study, DL models were employed to predict interactions between drugs and their respective targets, commonly referred to as drug-target interactions (DTI). These models were constructed based on the encoder and decoder architectures (Yu et al., 2022). The DL model scheme uses the SMILES string (Table 1) and amino acid sequence of the specific protein of interest as its input, employing more than 17 cutting-edge DL techniques to forecast indicators of drug efficacy (Figure 1). In this study, the researchers used the MPNN-CNN deep learning algorithms to predict affinity and specifically applied the MPNN model for ADMET predictions (Huang et al., 2020a).

**TABLE 1:** Top hit compounds obtained through SBVS, Zinc15-ID, SMILES names and Docking score visa Autodock4 (AD4) and Autodock Vina.

Top hits	ZINC15-ID	IUPAC Names	Docking score (AD4) kcal/mol	Docking score (Vina) kcal/mol
**09**	ZINC000003807186	(R)-2-((4-carboxy-4-(4-(((2,4-diaminopteridin-6-yl)methyl)amino)benzamido)butyl)carbamoyl)benzoic acid	−11.36	−11.2
**27**	ZINC000006118800	6-(naphthalen-2-ylsulfonyl)quinazoline-2,4-diamine	−11.78	−11.6
**41**	ZINC000005647485	(R)-6-(naphthalen-2-ylsulfinyl)quinazoline-2,4-diamine	−11.58	−11.0
**68**	ZINC000005891475	5-methyl-6-((quinolin-3-ylamino)methyl)pyrido[2,3-d]pyrimidine-2,4-diamine	−11.34	−11.0
**74**	ZINC000025968633	N-(2,4-diamino-5-methylquinazolin-6-yl)-2-(3,4-dichlorophenyl)acetamide	−11.56	−11.0
**85**	ZINC000003814848	6-(acridin-10(9H)-ylmethyl)pteridine-2,4-diamine	−11.75	−11.6
**99**	ZINC000005939955	6-((9H-carbazol-9-yl)methyl)pteridine-2,4-diamine	−11.76	−11.6
**180**	ZINC000003830554	3,3'-((1E,1′E)-[1,1′-biphenyl]-4,4′-diylbis(diazene-2,1-diyl))bis(4-aminonaphthalene-1-sulfonic acid)	−11.23	−11.1
MTX^a^	RD	(4-(((2,4-diaminopteridin-6-yl)methyl)(methyl)amino)benzoyl)-L-glutamic acid	−9.7	−9.8
PTX^b^	RD	(E)-(4-((2,4-diaminoquinazolin-6-yl)diazenyl)benzoyl)-L-glutamic acid	−10.1	−9.4

RD, Reference drug.

^a^ Methotrexate.

^b^ Phototrexate

### Density functional theory (DFT)

DFT methods are a reliable and proficient approach for the correct estimation of the electronic features of the compound (Hohenberg and Kohn, 1964). The complete geometry optimization for all the Zinc15 library database (https://zinc15.org/) compounds of MTX and PTX as shown in Supplementary Table S1 was conducted by applying the DFT methodology at the ground state level, employing three functional parameters. The Becke, Lee-Yang-Parr (B3-LYP) functional (Prieto-Martínez et al., 2018; Jemai et al., 2023) was used, consistently incorporating the GD3 correction for dispersion (DiLabio et al., 2013). The absence of imaginary frequencies observed during a harmonic vibrational analysis confirmed the existence of local energy minima in their character (Yang et al., 2019). The obtained results were utilized to calculate various electronic properties, such as the energy gap (Eg), for the top hits under investigation. The energy gap can be juxtaposed with specific molecular attributes, such as reactivity and electrical conductivity. The energies of the lowest unoccupied molecular orbital are commonly denoted as E_LUMO_, while those of the highest occupied molecular orbital are denoted as E_HOMO_. In addition to the aforementioned electronic properties, the values of physiochemical descriptors such as hardness (η), softness (S), chemical potential (µ), and electrophilicity index (ω) were determined employing Koopman’s theorem (Tsuneda et al., 2010). The relationship between chemical stability and reactivity can be elucidated by considering the parameters of chemical hardness (η), electronegativity (ꭓ), and softness (S) (El-Shamy et al., 2022). The basis set for all examined structures was 6-311+g(d,p). The calculations were executed using the Gaussian09 software suite (Frisch, 2009), while the Chemcraft suite was used to visualize the HOMO-LUMO Frontier molecular orbitals (Kanagathara et al., 2022). The Gauss View utility was also employed for visualization (Hanwell et al., 2012).

### Molecular dynamics simulation

MD simulations provide information about the stability of the best complex (Rasheed et al., 2021). The following steps were employed to execute the MD simulation. The 3-dimensional (3D) models of the enzyme Human DHFR (**PDB ID: 1U72**) (Rose et al., 2010) in complex with methotrexate molecule were exported to the.pdb format using Pymol (DeLano, 2002). The dynamic behavior of the complexes was assessed through MD simulation using the GROMACS software package (version 2022.2) (Bekker, 1993; Ganesan et al., 2017; Thalla et al., 2020). The protein topology was generated using the CHARMM27 force field (Schmid et al., 2011) through the pdb2gmx tool. The ligand topology was also created using the SwissParam server (Van Aalten et al., 1996). The complexes were subsequently introduced into the system upon implementing the force field. The solvation of the system was carried out using the TIP3P water model (Mark and Nilsson, 2001). A cubic box with dimensions greater than 1 nm from the protein’s edge was employed and periodic boundary conditions were applied. The system was rendered inert by introducing Na^+^ ions, followed by the execution of energy minimization for a total of 50,000 steps utilizing the steepest descent algorithm. Subsequently, a 100 picosecond (ps) NVT simulation at a temperature of 300 Kelvin (K) was conducted, followed by 100ps NPT simulation to achieve equilibrium for the entire system. The Leapfrog algorithm was utilized in the constant-temperature, constant-pressure (NPT) ensemble to independently couple each component, including the protein, ligand, water molecules, and ions (Van Gunsteren and Berendsen, 1988). The temperature and pressure coupling constants for the Berendsen method were assigned values of 0.1 and 2, respectively. These values were chosen to maintain a stable environment for the system, with a temperature of 300K and a pressure of 1 bar (Berendsen et al., 1995). The molecular dynamics (MD) simulation was conducted for 100 nanoseconds under isothermal and isobaric conditions in an ensemble at 300 Kelvin. The time constant for pressure coupling was configured to 1 picosecond to ensure a constant pressure of 1 bar.

Additionally, the LINCS algorithm (Hess et al., 1997) was employed to enforce constraints on bond lengths. The Van der Waals and Coulomb interactions were truncated at a distance of 1.2 nm. The PME algorithm (Di Pierro et al., 2015), integrated into GROMACS, was employed to minimize the error resulting from truncation. The trajectory files were visualized using Visual Molecular Dynamics (VMD) 1.9.2 (Humphrey et al., 1996) and analyzed using the in-house developed tool HeroMD Analysis (Devi et al., 2021; Rawat et al., 2021) and Xmgrace 5.1.25 (Vaught, 1996).

## Results and discussion

### Molecular docking results

This study has yielded significant findings in the realm of virtual screening and QSAR profiling utilizing DL techniques, alongside an evaluation of ADMET properties. The detailed results, including Supplementary Tables S1, S2, showcase the comprehensive outcomes of our investigation.

A total of eight compounds represented in Table 1 exhibited docking scores surpassing those of conventional drugs. The highest-ranking results underwent additional examination utilizing DL algorithms and QSAR. Thus, the utilization of DL models facilitated the prediction of drug affinity and the assessment of protein-ligand complex stability (Rasheed et al., 2021; Aziz et al., 2022a).

**FIGURE 2:**
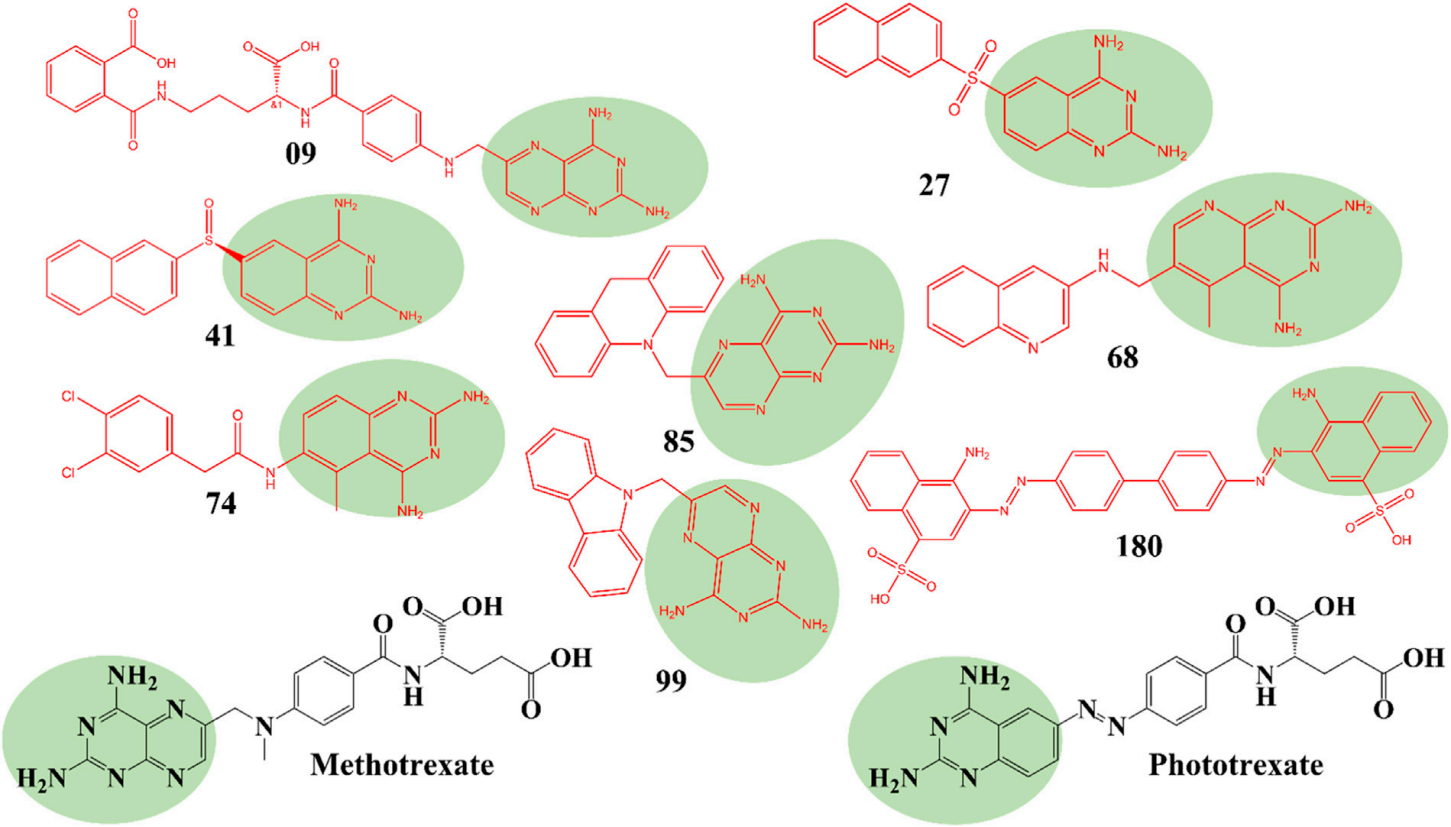
Top hit compounds obtained through SBVS utilizing a common Pharmacophore similar to the standard drugs MTX and PTX (green highlighting is showing same shared pharmacophore).

The structures of SBVS are presented in Figure 2, which highlights the most significant hits. It is important to note that each occurrence exhibits a shared pharmacophore with established MTX and PTX, recognized as inhibitors of DHFR. A common pharmacophore indicates that these compounds can bind to the active site of DHFR in a way that is analogous to MTX and PTX.

The implementation of a shared pharmacophore with established DHFR inhibitors presents numerous benefits. Initially, it creates a foundation for these compound’s logical development and improvement to augment their inhibitory efficacy and specificity towards DHFR. Through comprehension of the fundamental structural components accountable for binding, alterations can be implemented to enhance the potency and effectiveness of the compounds. In addition, a common pharmacophore among these compounds implies that they may operate through comparable pathways such as MTX and PTX, thereby reinforcing their viability as DHFR inhibitors for addressing DHFR-linked malignancies.

**FIGURE 3:**
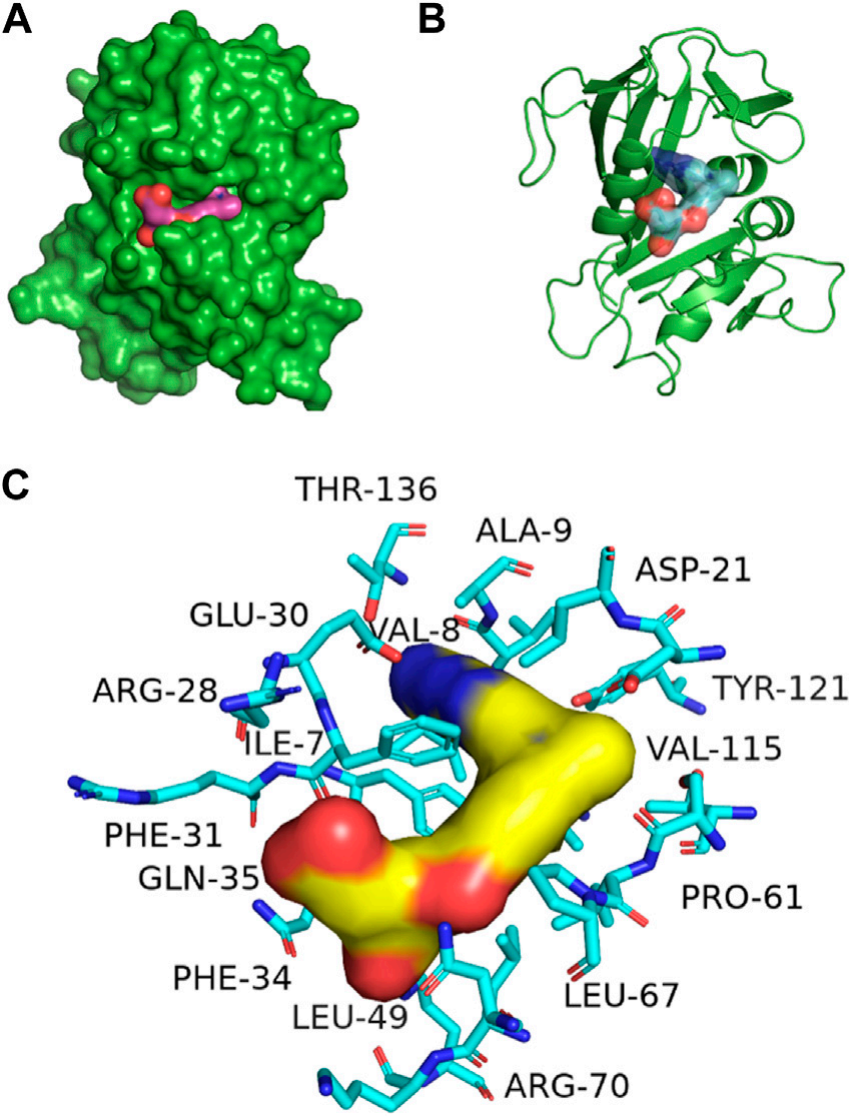
3D representation of 1U72 protein structure **(A)** Catalytic domain, **(B)** ligand binding pocket **(C)** binding site interaction with key amino acids, some amino acids are hidden behind ligand also.

**FIGURE 4:**
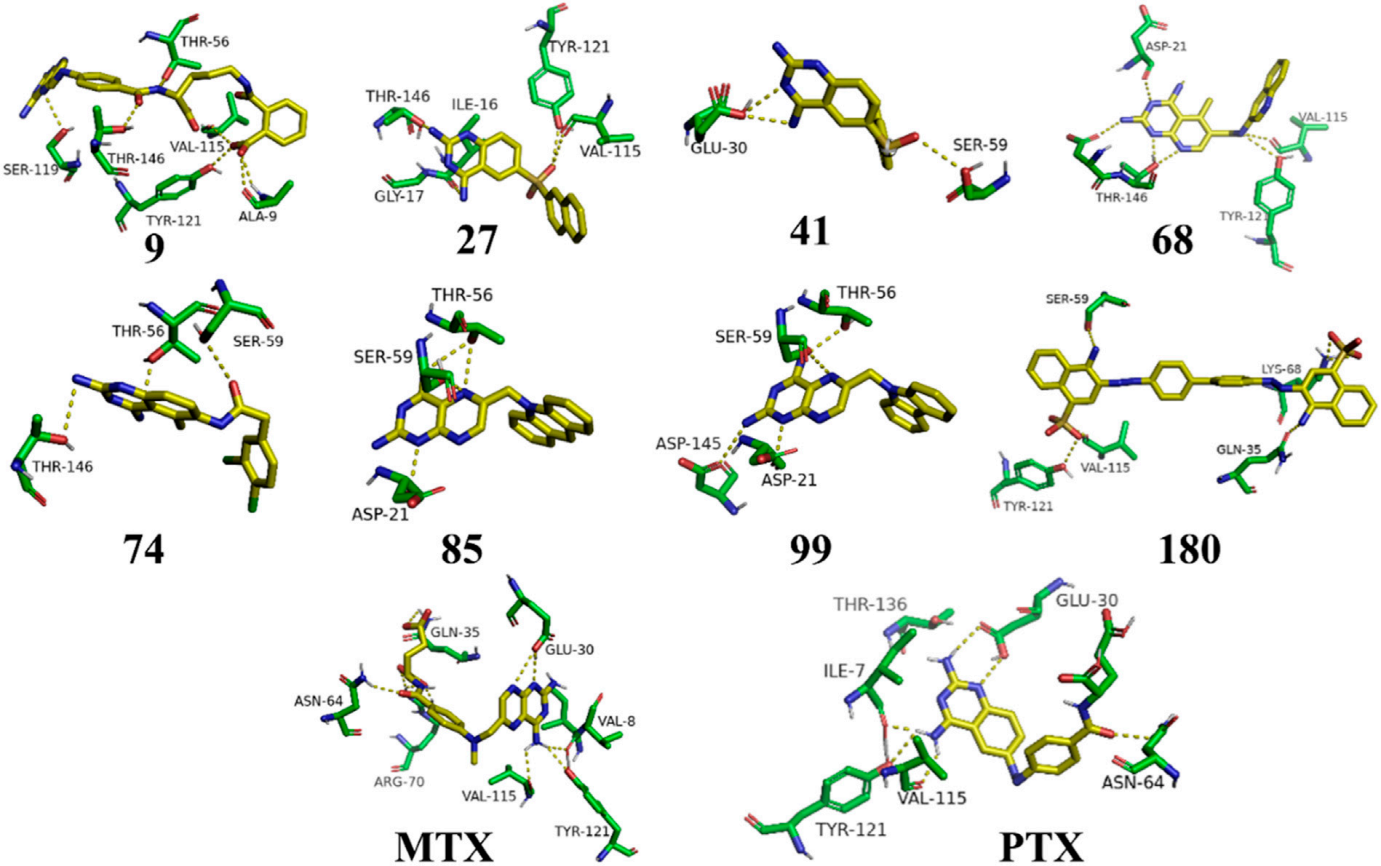
3D representation of H-bonding interactions of top hit compounds with 1U72.

**FIGURE 5:**
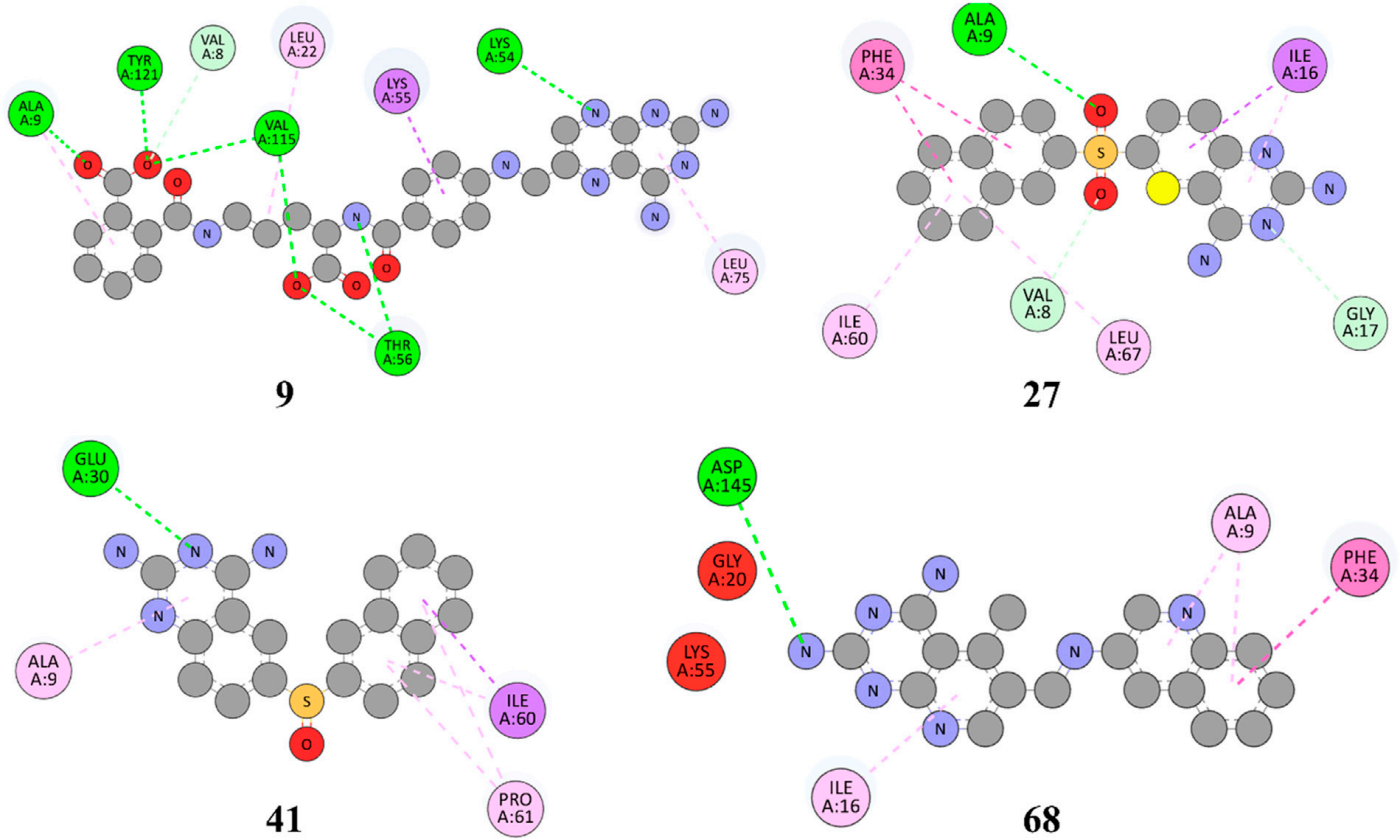
2D representation of hydrophobic and hydrogen bonding interactions of compounds **9, 27, 41** and **68** with 1U72.

**FIGURE 6:**
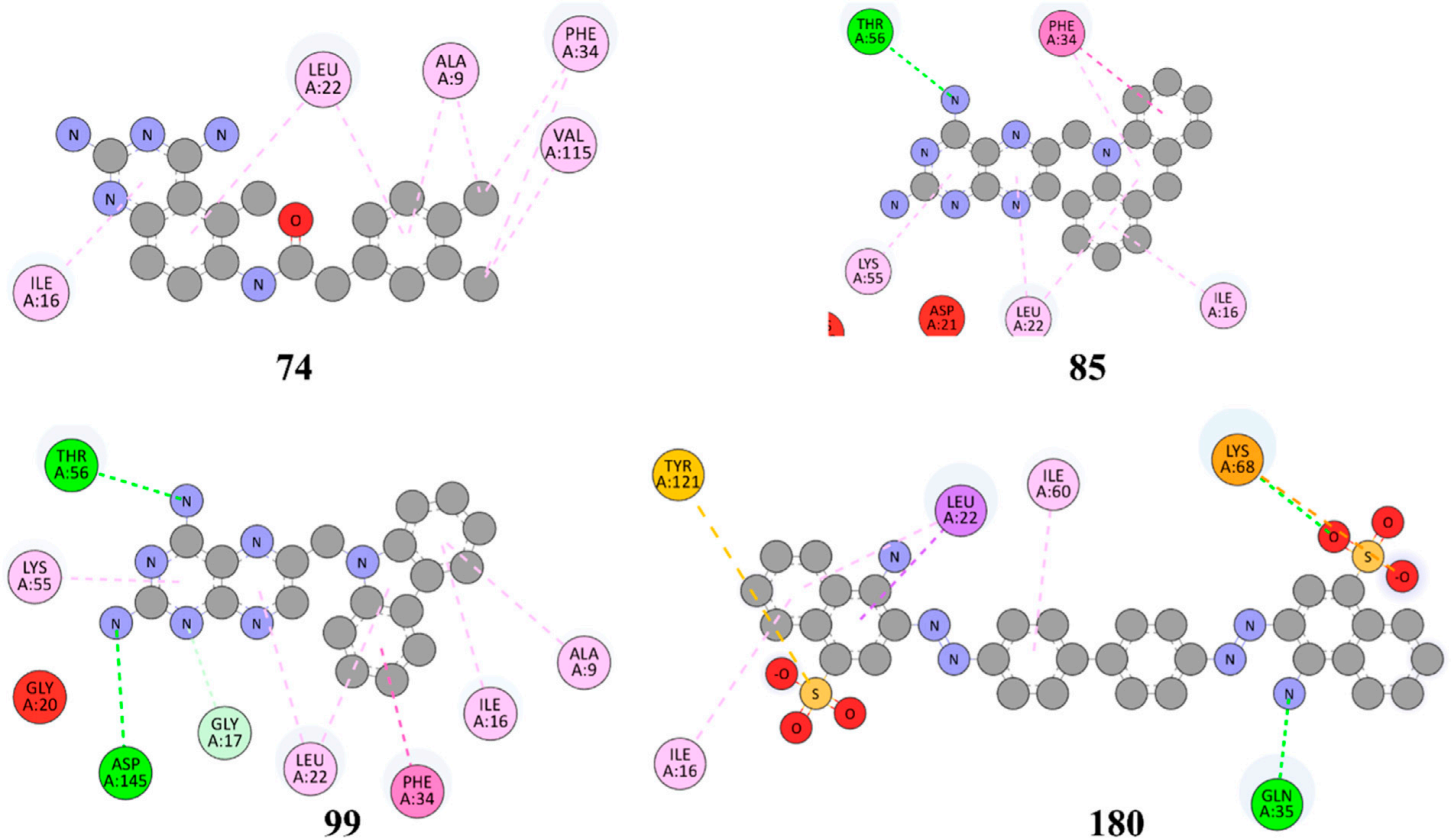
2D representation of hydrophobic and hydrogen bonding interactions of compounds **74, 85, 99** and **180** with 1U72.

**FIGURE 7:**
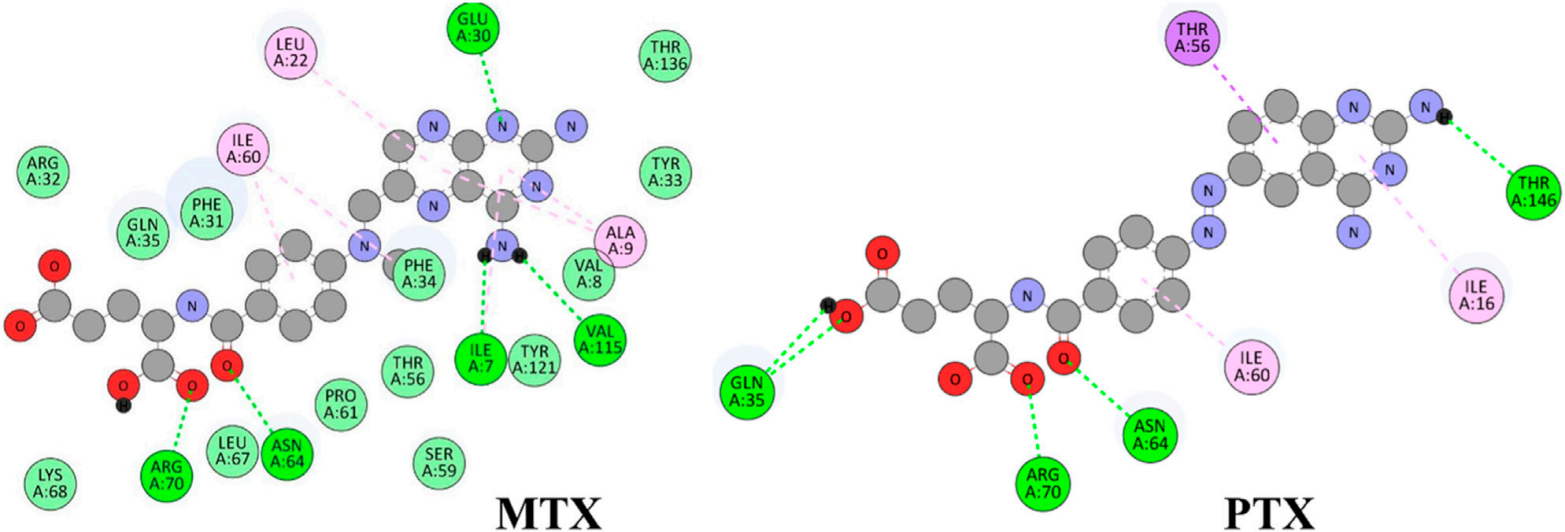
2D representation of hydrophobic and hydrogen bonding interactions of reference drugs **MTX** and **PTX** with 1U72.

Figure 3 represents the 3D protein structure of 1U72 and catalyic binding pocket. The molecular docking results of the top hits with the lowest binding energy are illustrated in Table 2. The 3D hydrogen bonding interactions of top hits with residual amino acids within the binding pocket of 1U72 are shown in Figure 4 whereas the 2D interactions of top hits and reference compounds are presented in Figures 5–7.

**TABLE 2:** The binding interactions and distance (Å) of top hit compounds with amino acid residues.

Top hits	Amino acid residues	Type of interactions	Distance in Å	Top hits	Amino acid residues	Type of interactions	Distance in Å
**9**	ALA9	H-bond	2.256	**74**	ALA9	Hydrophobic	3.707
	LYS54	H-bond	2.798		VAL115	Hydrophobic	4.425
	THR56	H-bond	2.991		PHE34	Hydrophobic	4.980
	THR56	H-bond	2.959		PHE34	Hydrophobic	4.554
	VAL115	H-bond	3.255		LEU22	Hydrophobic	5.208
	VAL115	H-bond	3.302		ILE16	Hydrophobic	5.243
	TYR121	H-bond	2.767		ALA9	Hydrophobic	5.416
	VAL8	H-bond	3.620		LEU22	Hydrophobic	5.476
	LYS55	Hydrophobic	3.767				
	LEU22	Hydrophobic	4.925				
	LEU75	Hydrophobic	5.488				
	ALA9	Hydrophobic	4.691				
**27**	ALA9	H-bond	2.841	**85**	THR56	H-bond	3.027
	VAL8	H-bond	3.436		PHE34	Hydrophobic	4.249
	GLY17	H-bond	3.735		LEU22	Hydrophobic	5.390
	ILE16	Hydrophobic	3.978		PHE34	Hydrophobic	5.493
	PHE34	Hydrophobic	4.363		ILE16	Hydrophobic	4.723
	PHE34	Hydrophobic	3.992		LYS55	Hydrophobic	5.230
	ILE16	Hydrophobic	4.681		LEU22	Hydrophobic	5.347
	ILE60	Hydrophobic	4.907				
	LEU67	Hydrophobic	5.396				
**41**	GLU30	H-bond	2.170	**99**	ASP145	H-bond	3.378
	ILE60	Hydrophobic	3.918		THR56	H-bond	2.961
	ALA9	Hydrophobic	4.293		GLY17	H-bond	3.439
	ILE60	Hydrophobic	4.570		PHE34	Hydrophobic	4.379
	PRO61	Hydrophobic	5.441		LEU22	Hydrophobic	5.275
	PRO61	Hydrophobic	5.346		ALA9	Hydrophobic	5.453
					ILE16	Hydrophobic	4.841
					LYS55	Hydrophobic	5.183
					LEU22	Hydrophobic	5.206
**68**	ASP145	H-bond	3.178	**180**	LYS68	Electrostatic	5.087
	PHE34	Hydrophobic	4.443		LYS68	H-bond	2.796
	ILE16	Hydrophobic	4.652		GLN35	H-bond	3.269
	ALA9	Hydrophobic	4.840		LEU22	Hydrophobic	3.781
	ALA9	Hydrophobic	5.087		TYR121	Hydrophobic	5.795
					ILE60	Hydrophobic	4.656
					ILE16	Hydrophobic	5.496
					LEU22	Hydrophobic	5.232
**MTX**	GLU30	H-bond	1.951	**PTX**	GLU30	H-bond	2.020
	ASN64	H-bond	2.307		GLN35	H-bond	2.990
	ARG70	H-bond	1.817		ASN64	H-bond	2.317
	VAL115	H-bond	2.438		ARG70	H-bond	1.813
	ILE7	H-bond	2.125		ARG70	H-bond	2.335
	ILE60	Hydrophobic	5.205		GLU1	H-bond	2.407
	ILE60	Hydrophobic	4.361		GLU1	H-bond	2.225
	ALA9	Hydrophobic	4.870		ILE7	H-bond	1.820
	LEU22	Hydrophobic	5.053		VAL115	H-bond	2.244
	ILE7	Hydrophobic	5.305		GLU30	H-bond	2.328
	ALA9	Hydrophobic	4.180		PRO61	H-bond	3.738
					ILE60	Hydrophobic	4.654
					LEU67	Hydrophobic	5.207
					PHE31	Hydrophobic	5.147
					ILE60	Hydrophobic	4.132
					ALA9	Hydrophobic	5.180
					LEU22	Hydrophobic	5.098
					ILE7	Hydrophobic	5.367
					ALA9	Hydrophobic	4.180

The Figure 5 and Table 2 illustrate that compound 9 exhibited binding interactions with amino acid residues VAL08, ALA09, VAL115, and TYR121, which are also known to interact with MTX and PTX. It is worth noting that compound 9 displayed a greater number of H-bonding interactions with their corresponding distance in angstroms, reflecting that ALA9 (2.256 Å), LYS54 (2.2.798 Å), THR56 (2.991, 2.959Å), VAL115 (3.255 Å), VAL115 (3.302 Å), TYR121 (2.767 Å), and VAL8 (3.620 Å), thus suggesting the possibility of better anticancer potential compared to other selected top hits. However, it is important to acknowledge that these findings are based on *in silico* analysis, and further experimental validation would be necessary to confirm the anticancer efficacy of compound 9. The sulfonyl oxygen in compound **27** formed H-bonds with ALA9 (2.841 Å), VAL8 (3.436 Å), and GLY17 (3.735 Å), and aromatic rings displayed π-π/π-σ interactions with ILE16, PHE34, ILE60 and LEU67 suggested a favorable binding mode for compound **27**. In comparison, **MTX** and **PTX** exhibited similar H-bonding interactions with amino acid residues, emphasizing the potential of compound **27** as a promising anticancer candidate. The sulphone moiety of compound-**41** could not interact with the receptor due to the steric effect of adjacent ring systems. However, the ring system is engaged in H-bonding interactions with GLU30 at a distance of 2.170 Å and hydrophobic interactions with ALA9, ILE60, and PRO61 demonstrating a favorable binding profile for compound **41**. In comparison, **MTX** and **PTX** primarily formed H-bonds with specific residues, highlighting the distinct binding characteristics of compound **41**. Compound **68** formed H-bonds with ASP145 at the relevant distance of 3.17 Å. PHE34, ILE16, PHE34, and ALA9 depicted the hydrophobic interactions thus displaying favorable binding interactions. These H-bonding interactions contribute to its potential as an effective anticancer agent. **MTX** and **PTX** also exhibited H-bonding and hydrophobic interactions, demonstrating similar binding patterns to compound **68**.

The results in Figure 6 and Table 2, Compound **74** showed hydrophobic interactions with ALA9, VAL115, PHE34, LEU22, and ILE16, indicating its ability to interact favorably with the target residues. The hydrophobic interactions observed for compound **74** suggest a potential role in disrupting the hydrophobic core of the target protein. MTX and PTX similarly engaged in hydrophobic interactions, demonstrating comparable binding characteristics to compound **74**. Compound **85** formed H-bonds with THR56 and LEU22 at the distance of 3.027 and 4.249 Å, highlighting its favorable binding interactions. Additionally, it exhibited hydrophobic interactions with PHE34, LEU22, ILE16 and LYS55. These binding features suggest a promising potential for compound **85** as an anticancer agent. **MTX** and **PTX** also demonstrated H-bonding and hydrophobic interactions, indicating similar binding profiles. Compound **99** was found to be engaged in H-bonding interactions with ASP145 (3.378 Å), THR56 (2.961 Å), and GLY17 (3.439Å) with different bond distances, suggesting a favorable binding affinity. It also displayed hydrophobic interactions with GLY17, PHE34, LEU22, ALA9, ILE16, and LYS55. These interactions contribute to the potential of compound **99** as an effective anticancer candidate. Comparatively, **MTX** and **PTX** exhibited similar H-bonding and hydrophobic interactions, aligning with the binding characteristics of compound **99**. The amino acid residues TYR 121 LYS68 interacted with compound **180** by electrostatic interactions whereas GLN35 (3.269 Å), showed H-bonding interactions with aromatic amino nitrogen. The hydrophobic interactions via pi-sigma bonding and π-π stacking with ILE16, LEU22 and ILE60 are crucial for favorable electron correlation. Our analysis unveiled a robust connection between the binding modes of studied ligands, MTX and PTX, highlighted by hydrogen bonds that play a crucial role in stabilizing interactions. In addition, the distances and angles of the hydrogen bonds were thoroughly analyzed to confirm the optimal hydrogen bonding for both ligands. By aligning these interactions with the known binding mode of MTX and its conformation, the biological significance of the binding poses of studied ligands and PTX has been established. This explanation offers valuable insights into the structural foundation of ligand-protein interactions and highlights the potential therapeutic significance of studied ligands and PTX as a new version of MTX. Overall, these interactions indicate a unique binding profile for compound **180** and exhibited binding similarities with MTX and PTX. **MTX** and **PTX** binding interactions are shown in Figure 4 (3D interactions) and Figure 7 (2D interactions).

The findings from the molecular docking analysis demonstrated the varied binding interactions between the top-hit compounds and the specific amino acid residues of the target. The observed compounds exhibited a synergistic interplay between H-bonding and hydrophobic interactions, suggesting their promising utility as efficacious agents for combating cancer. Although **MTX** and **PTX** displayed different binding patterns, the top-hit compounds exhibited comparable or superior binding characteristics. This implies that the compounds with the highest activity show considerable promise as innovative agents for combating cancer, thus justifying the need for additional research and refinement.

## DL model results

The DL-based QSAR model outcomes for all 271 compounds from the Zinc15 library have been meticulously documented and can be found in Supplementary Figures S1–S17. These figures depicted the ADMET profiling based on our DL-driven model, presenting a comprehensive analysis of key properties and characteristics for each compound in the library. These findings highlighted the potential efficacy and safety profiles of these compounds, making them crucial for further drug discovery and development efforts.

### DL based QSAR

The current study employed the multiple linear regression (MLR) method to conduct regression analysis (Liu and Long, 2009), as illustrated in Table 3. As shown in Supplementary Table S1, the dataset was partitioned into separate training and test sets. The analysis was conducted using the substitution groups and the inhibitory activity of compounds within the dataset. The training dataset was utilized for constructing the QSAR model, while the test dataset was employed to assess the predictive ability of the developed models. A dataset comprising 271 compounds, shown in Supplementary Table S1 from the Zinc15 library, was used in a DL model to forecast ADMET properties. Subsequently, the DL-based ADMET predictions were employed to construct a model.

**TABLE 3:** QSAR-based model representation with equation parameters.

MLR QSAR model equation		Fitting parameters	
DL-ADMET Equation parameters	Y1 = - 0.5310 (±0.0780) X1 + 0.0717 (±0.1912) X2 + 0.2381 (±1.0861) X3 + 0.0997 (±0.0322) X4 + 0.0131 (±0.0078) X5 - 0.3159 (±0.0847) X6 - 0.0569 (±0.0061) X7 - 0.0235 (±0.0053) X8 - 0.0000 (±0.0051) X9 + 0.0065 (±0.0026) X10 + 0.0109 (±0.0034) X11 + 0.0035 (±0.0032) X12 - 0.0126 (±0.0057) X13 - 0.6279 (±0.6323) X14 + 0.0011 (±0.4962) X15 + 0.0338 (±0.0066) X16 + 28.3293 (±8.3012) (n = 229; R = 0.897; s = 0.293; F = 54.714; *p* < 0.0001; Q2 = 0.773; SPress = 0.316; SDEP = 0.305)	**N**	229
		**K**	16
		**R** ^**2**^	0.81
		**R** ^**2**^ **-Adj.**	0.79
		**S**	0.29
		**F**	54.71
		**P**	0
		**Q** ^**2**^	0.77
		**SPress**	0.32
		**SDEP**	0.31
		**C.V.**	4.74

Y1 = pIC_50_, X1 = Solubility, X2 = Lipophilicity, X3 = (Absorption) Caco-2, X4 = (Absorption) HIA, X5 = (Absorption) Pgp, X6 = (Absorption)Bioavailability, X7 = (Distribution) BBB, X8 = (Distribution) PPBR, X9 = (Metabolism) CYP2C19, X10 = (Metabolism) CYP2D6, X11 = (Metabolism) CYP3A4, X12 = (Metabolism) CYP1A2, X13 = (Metabolism) CYP2C9, X13 = (Metabolism) CYP2C9, X14 = (Excretion) Half-life, X15 = (Excretion) Clearance, X16 = Clinical Toxicity.

The selection process was guided by a focus on structural similarity to established anticancer drugs, emphasizing compounds with analogous features and characteristics. Lipinski’s rules were applied to evaluate the resemblance of the investigated molecules to pharmaceutical compounds (Abdelrheem et al., 2020). A drug candidate that adheres to no more than one of the rules above will probably be pursued for development as a potential oral medication. Furthermore, we assessed the drug-like properties of the recently developed molecules using deep-learning ADMET techniques. This analysis aimed to identify the molecular structures exhibiting favorable oral drug administration characteristics. To validate this choice, an *in silico* assessment was conducted to evaluate the pharmacokinetic parameters of the selected compounds using ADMET prediction (Rifaioglu et al., 2019).

### Predicted binding affinities

By comparing the binding affinities of the compounds to the DHFR cancer target protein, it is possible to observe variations in their predicted IC_50_ values and pIC_50_ values, as shown in Table 4. Compound **68** exhibits notable characteristics concerning binding affinity, as evidenced by its predicted IC_50_ value of 78.66 nM and pIC_50_ value of 7.10. The data indicates that compound **68** exhibits a robust and efficacious binding affinity towards the DHFR oncogenic protein. Compound **74** demonstrates a notable binding affinity, as evidenced by its anticipated IC_50_ value of 62.46 nM and pIC_50_ value of 7.20. This observation suggests a robust binding affinity between the compound and the target protein, similar to compound **68**.

**TABLE 4:** DL model-based binding energies and predicted binding affinities.

Top hits	Binding energies kJ/mol	Predicted binding affinity (IC_50_) nM	pIC_50_ (predicted using deep learning model)
**09**	−46.89	218.22	6.66
**27**	−48.57	1,423.03	5.85
**41**	−46.05	1,394.69	5.86
**68**	−46.05	78.66	7.10
**74**	−46.05	62.46	7.20
**85**	−48.57	724.71	6.14
**99**	−48.57	1,587.48	5.80
**180**	−46.47	1,172.72	5.93
MTX	−39.38	129.91	6.89
PTX	−39.36	773.98	6.11

In contrast, it can be observed that compounds **27, 41,** and **180** exhibit moderate binding affinities, as their anticipated IC_50_ values fall within the range of 1,394.69 nM to 1,423.03 nM, thereby yielding pIC_50_ values that vary from 5.85 to 5.93. Although the binding affinity of these compounds is comparatively weaker than that of compounds **68** and **74**, they display significant interactions with the DHFR protein, which is a target for cancer treatment. Compounds **09, 85, 99, MTX,** and **PTX** exhibit reduced binding affinities, as indicated by their predicted IC_50_ values, which range from 129.91 nM to 2,187.48 nM and correspond to pIC_50_ values ranging from 5.80 to 6.89. The studied compounds display lower binding affinities towards the target protein than those above. To summarize, the binding affinities of the compounds **68** and **74** are the highest, while compounds **27, 41**, and **180** exhibit moderate affinities. Compounds **09, 85, 99, MTX,** and **PTX** demonstrate reduced binding affinities with DHFR target protein when they were subjected to the DL prediction model.

### MPNN-deep learning model-based ADMET

Table 5 presents a comparative analysis of the ADMET (absorption, distribution, metabolism, excretion, and toxicity) outcomes generated by the DL, highlighting notable patterns and variations across the compounds currently under study using the MPNN model. The solubility values, expressed in logarithmic units of mol/L, range from −3.93 to −5.45. The solubility of the compounds can be inferred from their respective negative values, where higher solubility is associated with more negative values. Compound **180** has the highest solubility with a value of −5.45, while compound **09** has the lowest solubility of −3.93. As a result, it is possible that compound **180** exhibits superior dissolution properties in comparison to the remaining compounds. The range of lipophilicity, as determined by the logarithmic ratio, falls between 0.19 and 2.69. The lipophilicity of compound **74** is the highest among the tested compounds, as indicated by its value of 2.69. This suggests that it has a greater tendency to dissolve in lipids or fats, with higher values indicating increased fat solubility. On the other hand, it can be observed that compound **09** displays the least degree of lipophilicity (0.19), thereby suggesting a comparatively reduced capacity for dissolution in fats.

**TABLE 5:** MPNN-Deep Learning model based ADMET, top hit compounds predicted results.

Top hits	Solubility Log mol/L	Lipophilicity Log-ratio	Absorption (HIA) %	Absorption (Pgp) %	Absorption (bioavailability F20) %	Distribution (BBB) %	Distribution (PPBR) %
**09**	−3.93	0.19	93.6	13	76.3	47.1	35.3
**27**	−4.77	1.89	98.61	12.7	78.46	60.38	90.89
**41**	−5.27	2.64	98.83	33.3	78.32	68.93	91.53
**68**	−4.05	2.38	98.4	16	78.16	68.24	55.5
**74**	−5.05	2.69	97.38	12.5	77.02	74.06	64.5
**85**	−4.38	2.45	98.92	21.3	78.46	74.68	73.47
**99**	−4.83	2.31	99.12	20.5	78.83	68.33	71.55
**180**	−5.45	1.22	97.44	12.9	77.94	43.47	94.11
MTX	−3.00/Soluble	0.00/No	91.51/Low	8.06/Yes	75.76/Yes	49.95/Yes	19.90/Yes
PTX	−3.94	0.14	93.24	8.76	75.96	52.15	41.96

Top Hits	(Metabolism) CYP2C19%	(Metabolism) CYP2D6%	(Metabolism) CYP3A4%	(Metabolism) CYP1A2%	(Metabolism) CYP2C9%	(Excretion) Half life hours	Clinical Toxicity %
**09**	7.5	4	9.7	9	3.2	8.26	32
**27**	37.46	16.66	23.16	61.66	8.75	8.11	31.64
**41**	50.74	17.93	28.66	79.34	6.84	7.98	32.24
**68**	39.35	39.73	47.01	90.49	6.67	7.98	34.64
**74**	70.8	21.66	38.74	80.44	30.32	7.97	32.66
**85**	42.84	24.28	27.57	79.94	21.42	7.94	34.88
**99**	23.53	12	27.71	90.5	12.67	7.97	35.2
**180**	94.11	14.59	13.86	28.4	61.74	6.53	8.3
MTX	3.66/good	3.14/good	4.22/good	5.02/good	1.42/good	8.26/Medium	31.78/Medium
PTX	4.04	5.00	3.93	18.69	1.45	8.33	30.12

HIA, human intestinal absorption; Pgp, permeability-glycoprotein; BBB, blood brain barrier; PPBR, plasma protein binding rati; MTX results obtained from literature incorporated as MTX, deep learning module value**/**Literature predictions (Uddin et al., 2019; Singh et al., 2022; Rana et al., 2019; Kawabata et al., 2013).

The absorption percentages, specifically related to human intestinal absorption (HIA), transportation by P-glycoprotein (Pgp), and Bioavailability-F20, have been included in the study for reference. These values indicate how well a compound is absorbed in the human intestines, the extent to which it is transported by P-glycoprotein, and its potential bioavailability, with a focus on Bioavailability-F20. These parameters are valuable for assessing a compound’s suitability for further development and its potential as a drug candidate. The values mentioned above denote the proportion of the substance assimilated following ingestion via the oral route. The oral absorption rates of the compounds were found to be high, as evidenced by the absorption (HIA) values ranging from 93.6% (compound **09**) to 99.12% (compound **99**). The absorption rate of compounds across the gastrointestinal tract through P-glycoprotein, an efflux transporter, exhibits a range of variability from 12.5% (compound **74**) to 33.3% (compound **41**). The bioavailability of the administered compounds, ranging from 76.3% (compound **09**) to 78.83% (compound **99**), pertains to the extent of absorption and represents the proportion of the dose that enters the systemic circulation. Distribution (BBB) and Distribution (PPBR) percentages are presented as distribution values. The distribution (BBB) parameter denotes the capacity of the compound to traverse the blood-brain barrier, with a range of values observed between 43.47% (compound **180**) and 74.68% (compound **85**). Elevated values indicate superior distribution across the blood-brain barrier. The distribution of a compound, as measured by its plasma protein binding rate (PPBR), indicates its affinity for binding to proteins in the bloodstream. The observed PPBR values for the investigated compounds range from 35.3% (compound **09**) to 94.11% (compound **180**).

The metabolic rates of specific cytochrome P450 enzymes (CYP2C19, CYP2D6, CYP3A4, CYP1A2, CYP2C9) that participate in drug metabolism are indicated as percentages in metabolism values. The metabolic profile of Compound **41** marks a significant metabolism rate by CYP3A4 (28.66%) and CYP1A2 (79.34%), implying that these enzymes are playing a crucial role in the compound’s metabolic pathway. The results indicate that Compound **180** undergoes considerable metabolism by CYP2C9, with a rate of 61.74%, suggesting a significant contribution of this enzyme in the metabolic process of this compound. The column “half-life” indicates the duration required for the concentration of a given compound within an organism to decrease by 50%. Compound **6** demonstrates a brief half-life of 6.53 h, implying a swift elimination rate. Conversely, compound **68** displays the lengthiest half-life of 8.33 h, indicating a more gradual elimination process.

The clinical toxicity values indicate each compound’s toxicity rating or score in the last column compound **180** exhibits the most favorable clinical toxicity ratings, with a value of 8.3%, respectively. Conversely, compounds **09, 27, 41, 68,** and **99** demonstrate clinical toxicity ratings ranging from 31.64% to 35.2%. The clinical toxicity ratings of compounds MTX and PTX are 31.78% and 30.12%, respectively, suggesting the presence of potential toxicity. Methotrexate, in particular, is known to be a highly toxic drug, classified as a cytotoxic agent. The level of toxicity can vary significantly based on factors such as dosage, duration of administration, individual patient characteristics, and other relevant determinants. Additionally, toxicity can indeed vary in terms of its impact and severity on patients. In brief, these DL model findings presented in Table 5 indicate that compound **180** manifests the most outstanding solubility, compound **74** displays the highest lipophilicity, compound **99** exhibits the highest rate of oral absorption, compound **85** demonstrates the highest distribution across the blood-brain barrier, compound **41** undergoes substantial metabolism by CYP3A4 and CYP1A2, compound **68** has the lengthiest half-life, and compounds **180** exhibit the least clinical toxicity ratings.

### DFT-based physiochemical descriptor profile

The physiochemical descriptors of the top hit compounds obtained through virtual screening and the standard anticancer drugs MTX and PTX were determined using DFT analysis and are presented in Table 6. The aforementioned descriptors in the table offer valuable insights into the electronic properties and reactivity of the compounds, thereby playing a crucial role in comprehending their behavior and potential applications. The electronic energy values obtained for the compounds ranged from −2,810.59 atomic units to −1,079.78 atomic units. Electronic energy is a comprehensive measure of the energy the electrons possess within a given system, and the molecular structure and composition of the compound determines its magnitude (Closs et al., 1986). The dipole moment, denoted in Debye units (D), is a quantitative measure of the spatial displacement between the positive and negative charges within a given molecule (Minkin, 2012). The dipole moment values presented in Table 6 exhibited a range spanning from 0.92 D to 9.31 D, which can be attributed to the polarity and asymmetry characteristics of the compounds. The E_Homo_ values, which denote the highest occupied molecular orbital energy, exhibited a range from −6.24 eV to −5.50 eV. These values determine compounds’ electron donation ability, where more negative E_Homo_ values indicate a greater propensity to donate electrons in chemical reactions. The values of E_Lumo_, which denote the energy of the lowest unoccupied molecular orbital, exhibited a range spanning from −2.73 eV to −1.62 eV.

**TABLE 6:** DFT electronic parameters of top-hit anticancer compounds and standard drugs.

Top hit lead compounds	Electronic energy (a.u)	Dipole moment (Debye)	E_Homo_ (eV)	E_Lumo_ (eV)	Energy gap (eV)	Ionization potential (eV)	Electron affinity (eV)
						(I) = -E_HOMO_	(A) = -E_LUMO_
**9**	−1989.98	9.31	−5.97	−2.58	3.39	5.97	2.58
**27**	−1,462.33	4.87	−6.24	−2.03	4.21	6.24	2.03
**41**	−1,387.09	2.13	−5.97	−1.80	4.17	5.97	1.80
**68**	−1,079.78	3.71	−5.89	−1.94	3.95	5.89	1.94
**74**	−1926.65	4.67	−5.67	−1.62	4.04	5.67	1.62
**85**	−1,156.02	3.47	−5.50	−2.28	3.22	5.50	2.28
**99**	−1,116.70	3.80	−5.81	−2.33	3.48	5.81	2.33
**180**	−2,810.59	4.32	−5.95	−3.09	2.85	5.95	3.09
**MTX**	−1,590.11	4.99	−5.82	−2.38	3.44	5.82	2.38
**PTX**	−1,533.50	0.92	−5.92	−2.73	3.20	5.92	2.73

The E_Lumo_ plays a crucial role in the acceptance of electrons. Compounds exhibiting lower E_Lumo_ values demonstrate a heightened capacity for electron acceptance. The energy gap, which is determined by the disparity between the E_Homo_ and E_Lumo_, explains the electron transfer capabilities of the compound. The observed energy gap values range from 2.85 eV to 4.21 eV, signifying the compounds’ diverse capacities for electron donation or acceptance, as shown in Table 7. The ionization potential is a fundamental concept in chemistry, which can be mathematically expressed as the negative of the highest occupied molecular orbital energy (E_Homo_). It signifies the minimum amount of energy needed to extract an electron from a molecule that is in a neutral state. This study’s range of ionization potential values spanned from 5.50 eV to 6.24 eV. This range suggests that the compounds examined in this research exhibit varying degrees of stability and resistance to remove electrons. The electron affinity of a molecule is determined by the energy change that occurs when an electron is acquired, and it is quantified as the negative value of the energy of the lowest unoccupied molecular orbital (E_Lumo_). The observed electron affinity values ranged from 1.62 to 3.09 eV, signifying the compounds’ inherent capacity to accept electrons. Supplementary Table S3 showing the optimized XYZ coordinates for top-hit anticancer compounds and reference drugs.

**TABLE 7:** E_HOMO_ and E_LUMO_ with energy gap (ΔE) values for top-hit compounds represented in electron volts (eV) and with frontial orbitals.

Top hits	EHomo (eV)	HOMO	ELumo (eV)	LUMO	Energy gap (ΔEeV)
**9**	−5.97	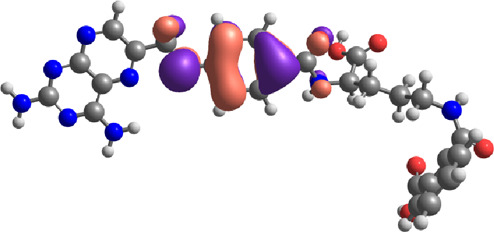	−2.58	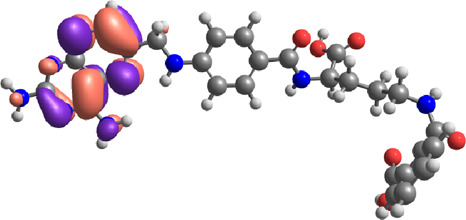	3.39
**27**	−6.24	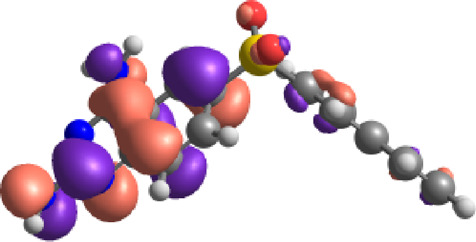	−2.03	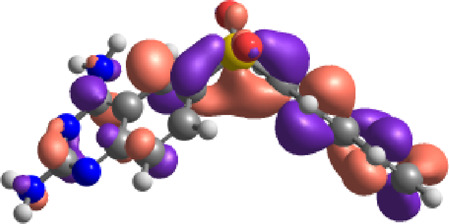	4.21
**41**	−5.97	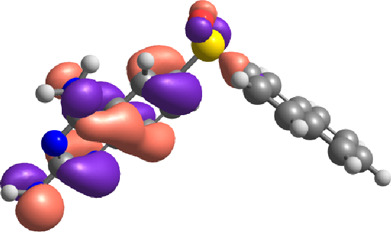	−1.80	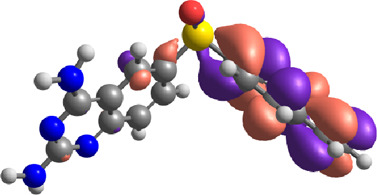	4.17
**68**	−5.89	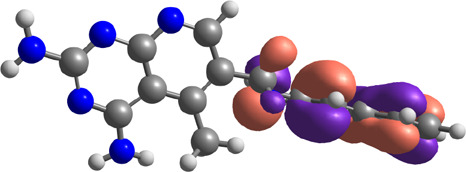	−1.94	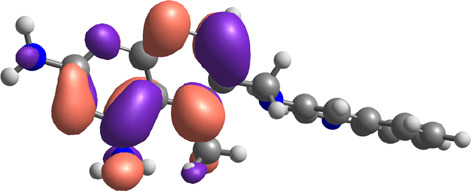	3.95
**74**	−5.67	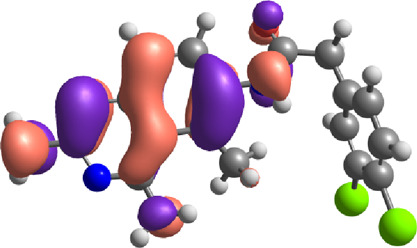	−1.62	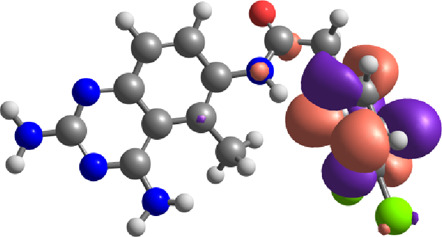	4.04
**85**	−5.50	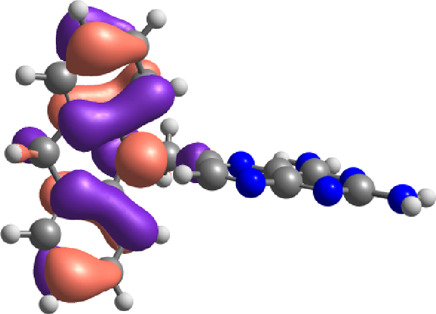	−2.28	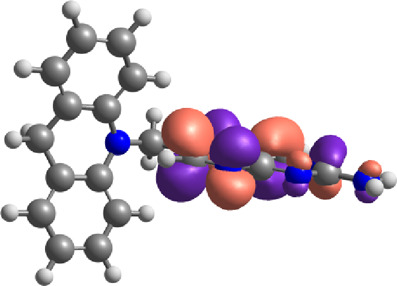	3.22
**99**	−5.81	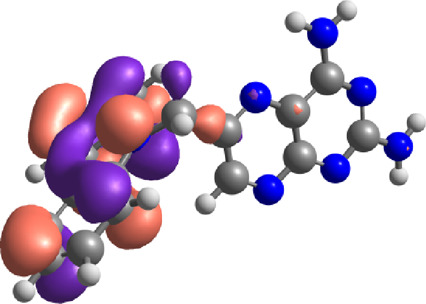	−2.33	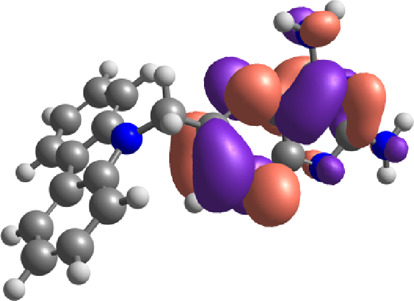	3.48
**180**	−5.95	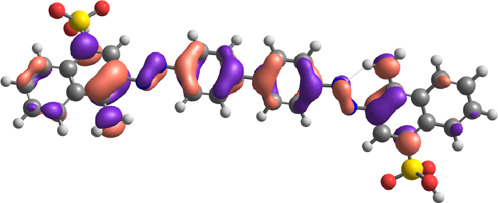	−3.09	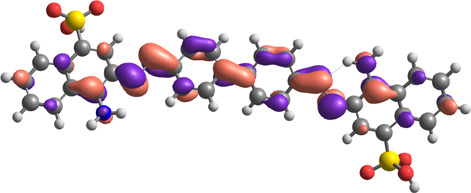	2.85
**MTX**	−5.82	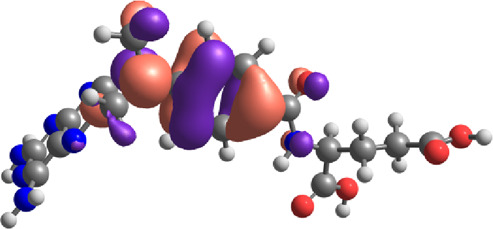	−2.38	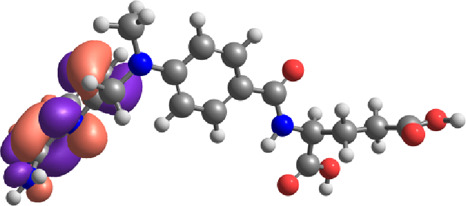	3.44
**PTX**	−5.92	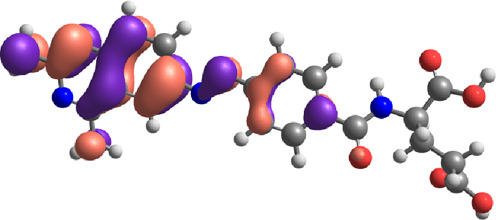	−2.73	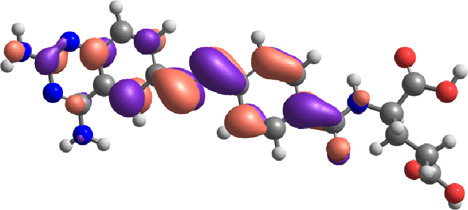	3.20

The chemical descriptors of the compounds were virtually screened as top hits, and the standard anticancer drugs MTX and PTX were assessed using DFT calculations. The results are presented in Table 8, which showcases the outcomes obtained from the DFT calculations. These descriptors provide valuable understandings of the compounds’ electronic structure, reactivity, and stability, elucidating their chemical behavior. The chemical potential (μ) is determined by the arithmetic mean of the ionization potential (I) and the electron affinity (A), and it signifies the inclination of a compound to either donate or accept electrons. The chemical potential values in Table 8 ranged from −4.52 eV to −3.65 eV. These values indicate the compounds’ electronic stability and capacity to engage in electron transfer phenomena. Electronegativity (χ) measures an element’s ability to attract electrons, calculated as the average ionization potential and electron affinity. It is a quantitative indicator of a compound’s capacity to attract electrons. The compounds exhibited a range of electronegativity values, ranging from 4.52 eV to 3.65 eV, indicating their diverse propensities to attract electrons during chemical reactions. The chemical hardness (η) is determined by taking half the difference between the ionization potential and the electron affinity. It characterizes the ability of a compound to resist alterations in its electronic configuration. The chemical hardness values in Table 8, range from 1.43 eV to 2.11 eV, suggesting the compounds’ inherent stability and capacity to endure electronic perturbations. Global softness (σ), which is the inverse of chemical hardness, offers valuable insights into the reactivity and vulnerability of a compound to electron transfer. The global softness values exhibited a range spanning from 0.48 eV^−1^ to 0.70 eV^−1^, whereby lower values were indicative of heightened electron transfer resistance and chemical reactivity. A compound’s electrophilicity (ω) can be determined by evaluating the square of its chemical potential divided by twice its chemical hardness. This parameter measures the compound’s capacity to accept electrons and engage in electrophilic reactions. The electrophilicity values exhibited a range spanning from 6.64 eV to 10.22 eV, thereby indicating the diverse propensities of the compounds to function as electron acceptors. Most of the selected top hits exhibited ionization potential, electrophilicity, and electron affinity values similar to those of the reference drugs, suggesting they behave electrochemically similarly to MTX and PTX under physiologic conditions (Avendaño, 2008). However, compounds 27 and 180 showed greater ionization potential and electrophilicity, which may contribute to improved interactions and potentially enhanced therapeutic efficacy. The literature also showed similar results of related studies with the DFT method use (Graffner-Nordberg et al., 2000; Zia et al., 2019; Aziz et al., 2022a).

**TABLE 8:** DFT-based physiochemical descriptors of top-hit anticancer compounds and standard drugs.

Top hits	Chemical potential (eV)	Electronegativity (eV)	Chemical Hardness (eV)	Global softness (eV^−1^)	Electrophilicity (eV)
	(μ) = - (I + A)/2	(χ) = (I + A)/2	(η) = (I - A)/2	(σ) = 1/η	(ω) = μ^2^/2η
**9**	−4.28	4.28	1.70	0.59	9.14
**27**	−4.14	4.14	2.11	0.48	8.55
**41**	−3.89	3.89	2.09	0.48	7.55
**68**	−3.92	3.92	1.98	0.51	7.66
**74**	−3.65	3.65	2.03	0.49	6.64
**85**	−3.89	3.89	1.61	0.62	7.57
**99**	−4.07	4.07	1.74	0.57	8.28
**180**	−4.52	4.52	1.43	0.70	10.22
**MTX**	−4.10	4.10	1.72	0.58	8.41
**PTX**	−4.33	4.33	1.60	0.63	9.35

The physicochemical and chemical descriptors of the top hit compounds identified through virtual screening and the standard anticancer drugs MTX and PTX were thoroughly examined using DFT analysis. The discoveries above contributed to an enhanced comprehension of the compounds’ electronic characteristics, reactivity, and stability, which are pivotal in assessing their potential efficacy as anticancer agents. The results obtained from this study thus established a valuable resource for future drug design efforts, as they contribute to the development of new compounds that exhibit improved therapeutic effectiveness and targeted anticancer properties and also contributed in alignment with the existing literature (Hobani et al., 2017; Mammen et al., 2020; Rana et al., 2020).

### Molecular dynamics simulation

#### Molecular dynamics simulation

**FIGURE 8:**
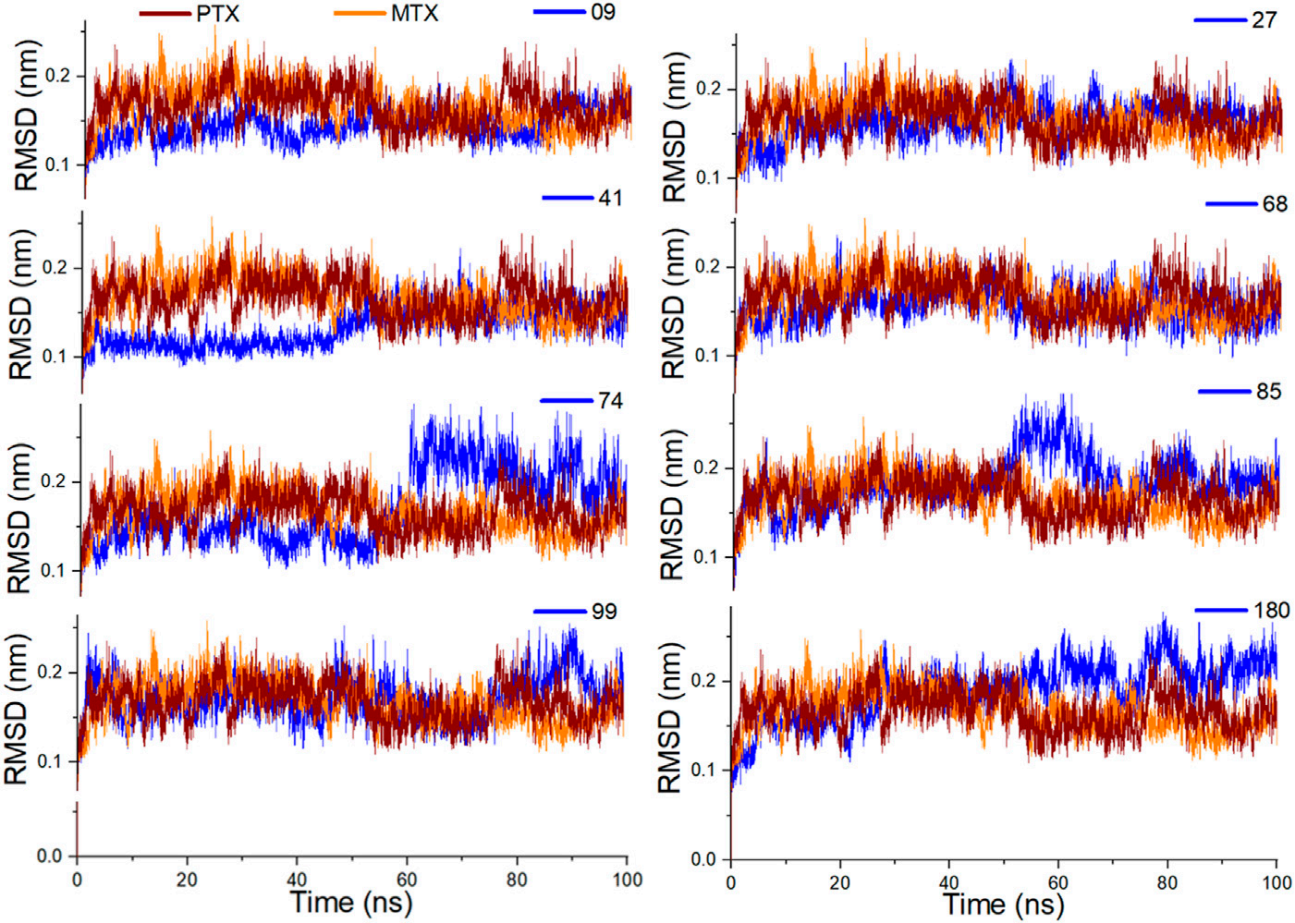
Analysis of MD simulation trajectories; Root mean square deviation (RMSD) of investigated ligands compared with RMSDs of MTX and PTX over time (100 ns).

**FIGURE 9:**
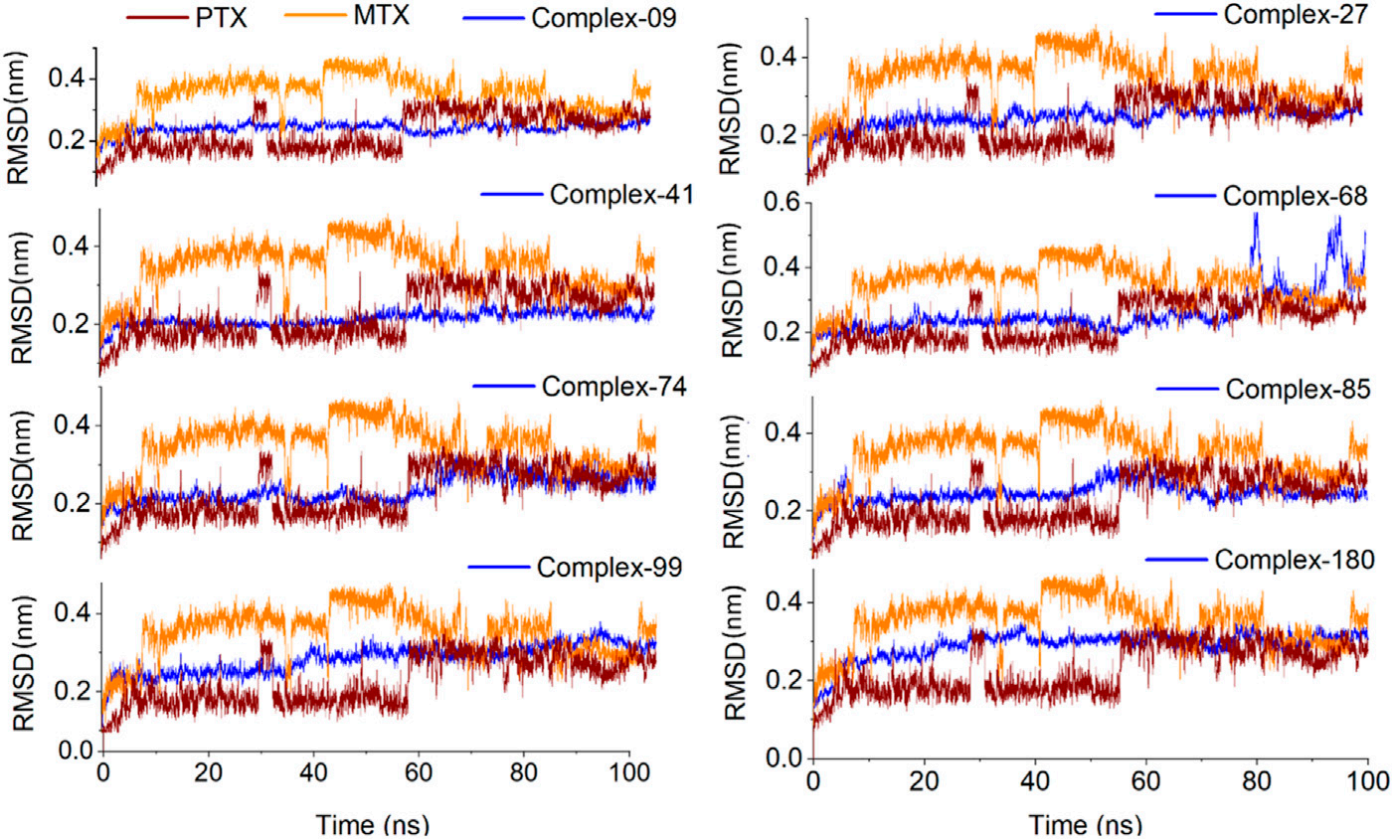
Analysis of MD simulation trajectories; Root mean square deviation (RMSD) of protein-ligand complex compared with RMSDs of MTX and PTX complex over time (100 ns).

To determine the binding stability of protein-ligand complexes, the MD simulation trajectories were evaluated by plotting the root mean square deviation (RMSD), root mean square fluctuation (RMSF), hydrogen-bond profile, solvent accessible surface area and radius of gyration. The root-mean-square deviation (RMSD) pattern provides remarkable insight into an average change in the displacement of atoms to a frame. Figure 8 presented the RMSD graphs of investigated ligands and compared them with those of MTX and PTX. The average RMSDs for the protein-ligand complexes of **09, 27, 41, 68, 74, 84, 99** and **180** were found to be 0.24nm, 0.20nm, 0.21nm, 0.26nm, 0.23nm, 0.24nm, 0.28nm and 0.29 nm respectively. To assess the impact of ligands on a conformational change in the protein structure, the RMSD of each system was compared with the reference compounds MTX and PTX, as shown in Figure 9. It has been observed that these ligand complexes exhibited only slight variations in RMSDs throughout the entire simulation, and the results were comparable with the reference MTX and PTX. The ligands **09, 27** and **41** reached equilibrium at about 6–10 ns and remained equilibrated for the entire simulation duration, demonstrating the high stability of these complexes with the target protein. The ligand **99** also formed a stable complex; only slight variation is observed in the last 20 frames of simulation. There is an increase in the RMSD values of the ligand **74** during 62–75 ns. After that, the system was again equilibrated and was found to be stable for the rest of the simulation. A significant variance was observed in the RMSD values of ligands **68** and **85** after about 55ns and remained least stable for the rest of the time.

**FIGURE 10:**
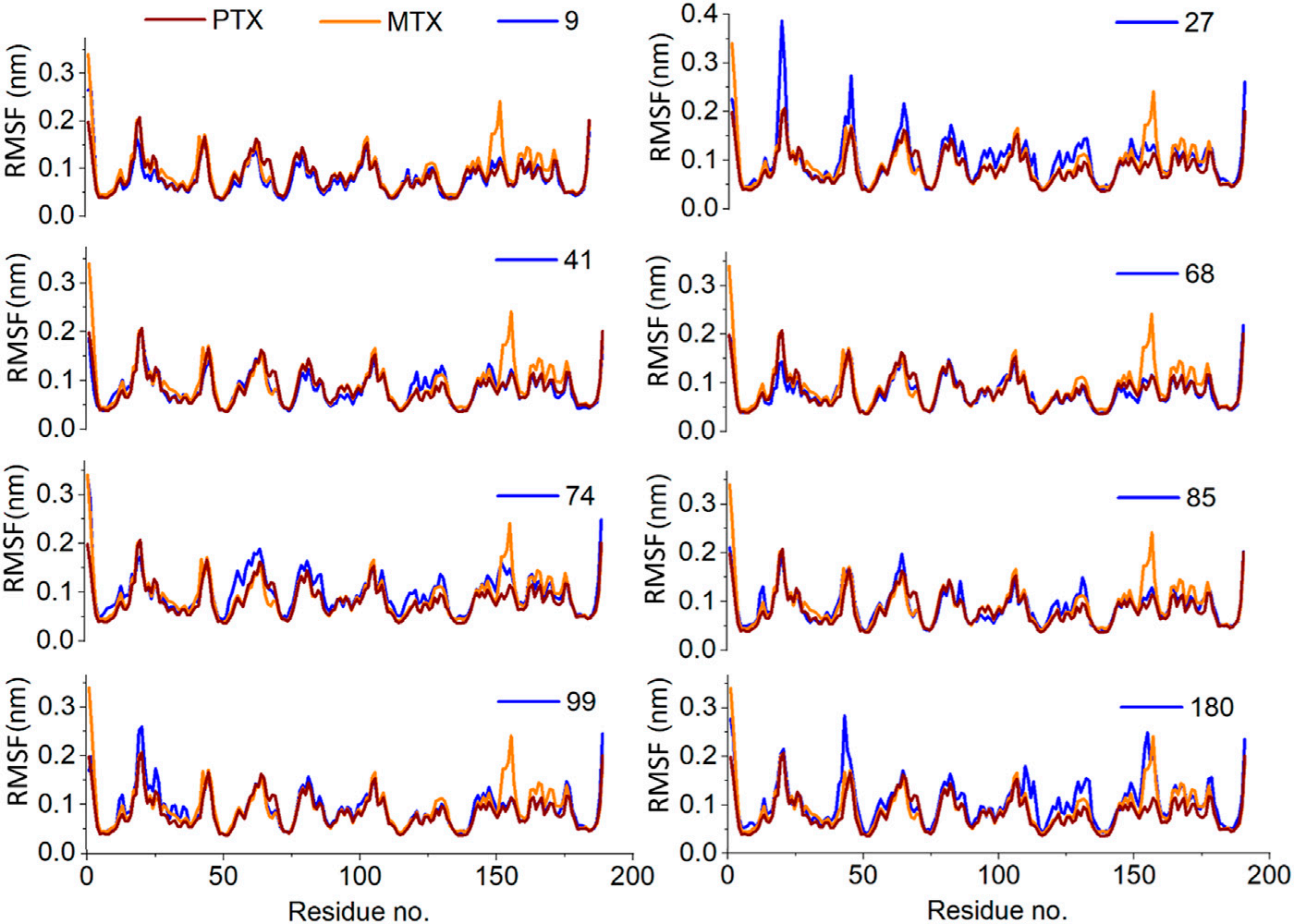
Analysis of MD simulation trajectories; Residue-wise root mean square fluctuation (RMSF) of protein ligand complex compared with RMSF of reference MTX and PTX complex.

The RMSF plots represent the protein regions that revealed a great fluctuation in their conformation during the simulation. In the present study, the receptor was found to be stable throughout the simulation. Only slight fluctuations were evident in the binding site region with RMSF values less than 0.2nm, considered negligible (Figure 10).

**FIGURE 11:**
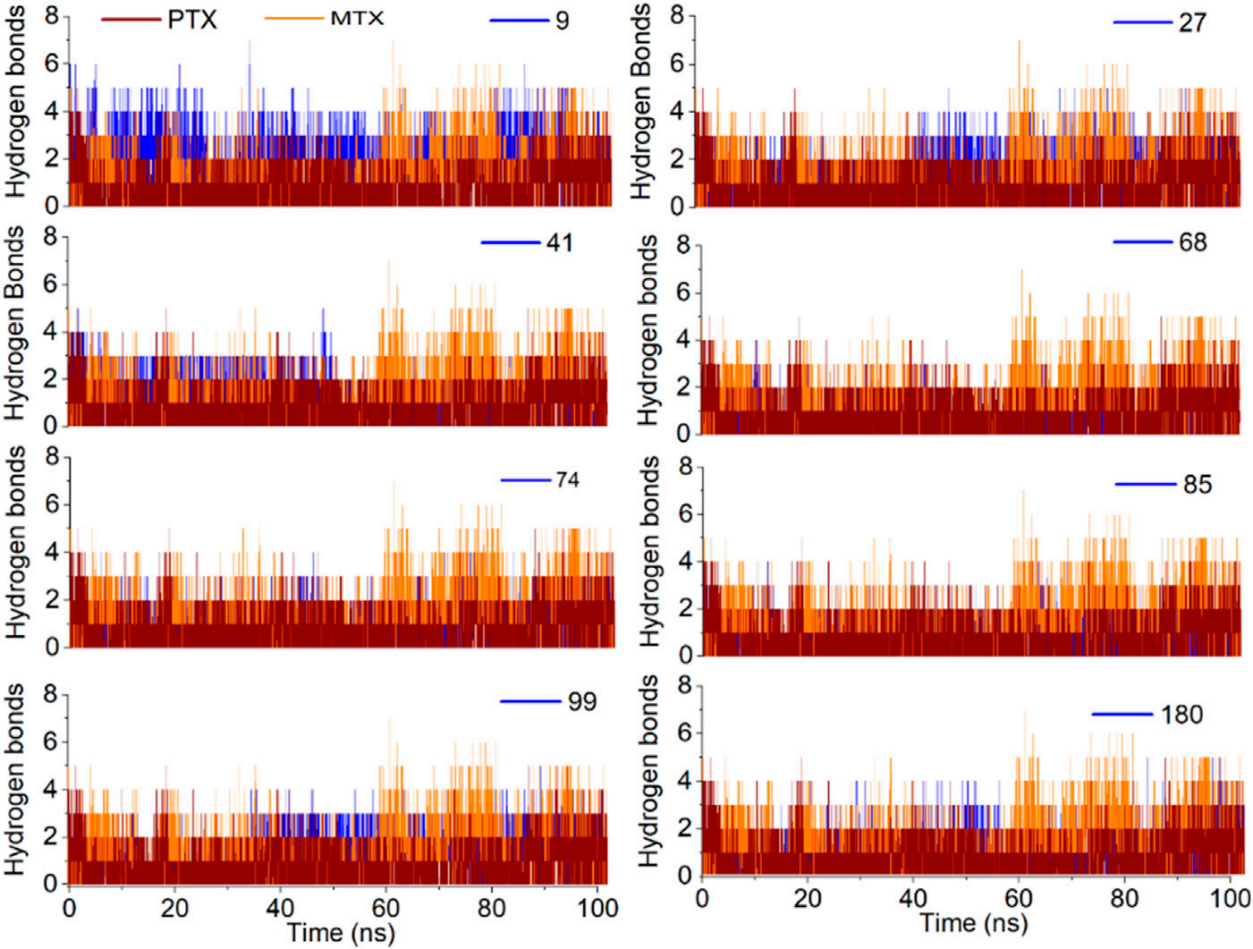
Analysis of MD simulation trajectories; H-bond profile of the protein-ligand complex over time (100 ns) compared with reference MTX and PTX complex.

The H-bond profile for all investigated ligands is illustrated in Figure 11 and found to be in good agreement with RMSD analysis. The most stable complexes of ligands **09, 27** and **41** were found to be due to the formation of 3–4 H-bonds with the target protein for most of the time during simulation. The ligands **74** and **99** formed 2–3 H-bonds, whereas ligand **180** formed 2–1 H-bonds over 100 ns simulation that depicts the relative binding stability of these ligands for time. Ligand **68** showed only 0–1 H-bond with high RMSDs in the last 20 ns of simulation, resulting in decreased stability of protein-ligand complex for the rest of the time. The binding stability of ligand **85** also decreased during 55–80 ns due to the decreased number of H-bonds with the target protein.

**FIGURE 12:**
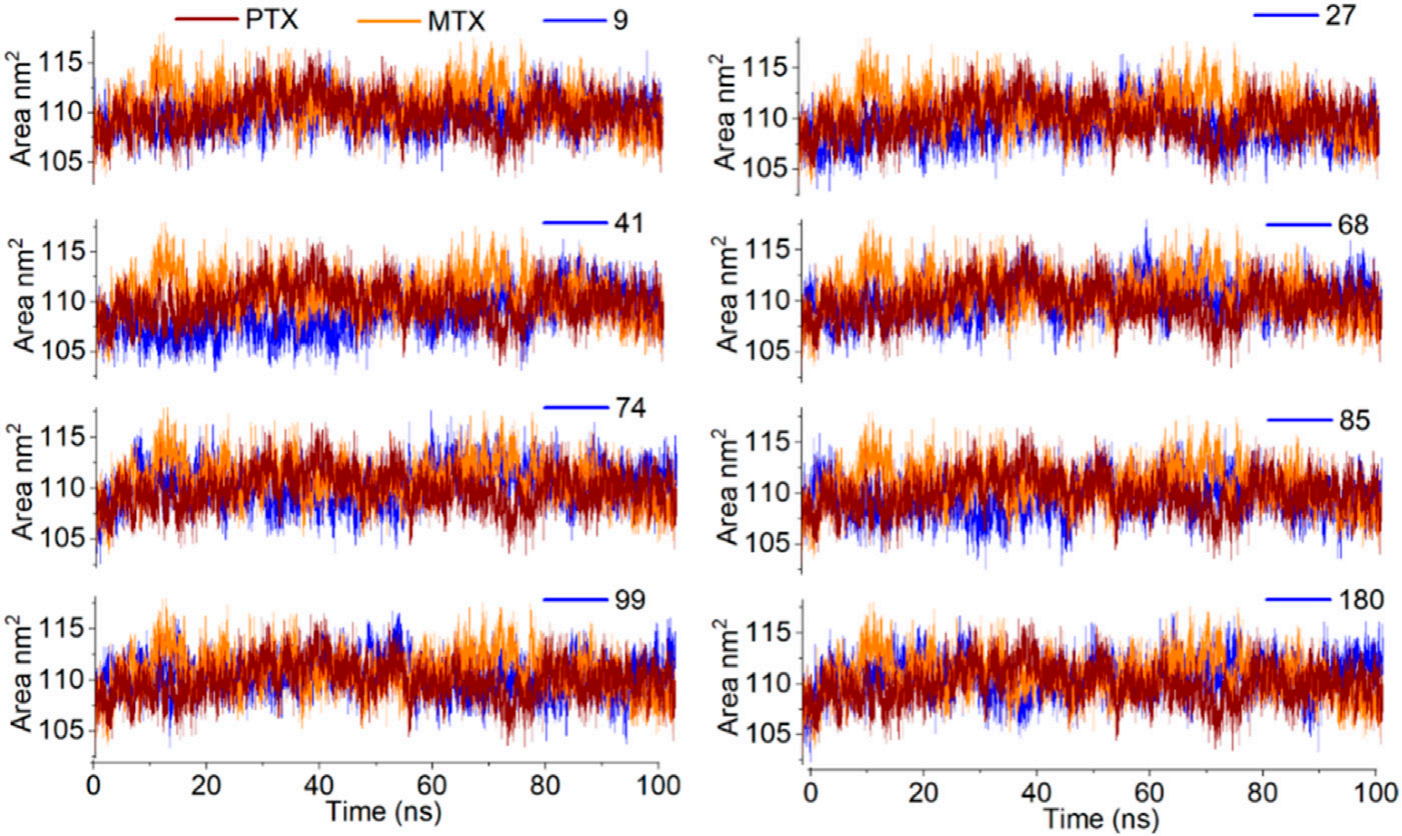
Analysis of MD simulation trajectories; Solvent accessible surface area of protein-ligand complex compared with reference MTX and PTX complex over time (100 ns).

Solvent accessible surface area (SASA) is attributed to the bimolecular surface area accessible to the solvent molecules. The greater the increase in the value for SASA concerning time, the lower the stability of the protein. In the current study, SASA of all complexes was observed between 100 nm^2^ to 120 nm^2^, with an average SASA value of 110 nm^2^, which is quite acceptable (Figure 12).

**FIGURE 13:**
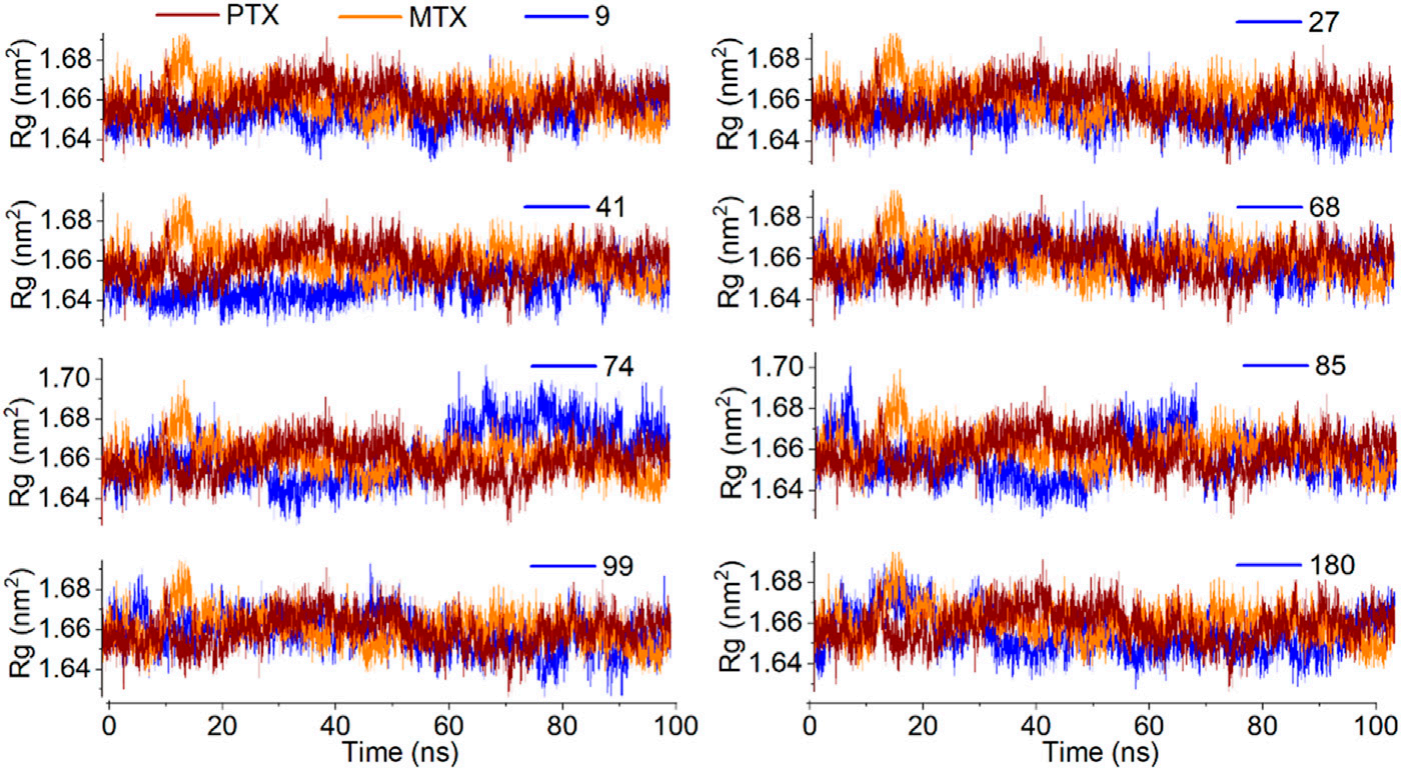
Analysis of MD simulation trajectories; Radius of gyration of protein-ligand complex compared with reference MTX and PTX complex over time (100 ns).

The analysis of the radius of gyration (Rg) provides information about the overall dimensions of the protein during the 100 ns simulation. All the protein-ligand complexes under investigation have shown a steady radius of gyration with an average value of 1.66 nm. The entire simulation indicates good protein stability (Figure 13).

Supplementary Figure S18 presented the snapshots of investigated ligands and reference compounds in complex with protein at 0 ns. Supplementary Figures S19–S28 illustrates the snapshots of investigated ligands along with MTX and PTX at 25 ns, 50 ns, 75 ns and 100 ns indicating the stability of investigated ligands and reference compounds within the binding pocket**.** The compound 68 complex with target protein is found to be stable up to 75 ns as depicted in Supplementary Figure S21 and at the last run of MD simulation, the compound is out of the binding pocket. These results are by the RMSD graph of compound 68-complex illustrating its unstability in the last 20 ns of MD simulation as shown in Figure 9.

The findings from Table 9, which outline the MM-PBSA of MM-GBSA binding free energies in kJ/mol, reveal that Ligands 9, 27, 68, 99, MTX, and PTX consistently demonstrate the lowest binding energies throughout the molecular dynamics (MD) simulation timeframe. This observation has important scientific implications, indicating that these ligands have high affinities for the target receptor or binding site during the simulation. The uniform low binding energies observed with various ligands suggest strong and enduring interactions with the receptor, demonstrating advantageous binding orientations and energetic environments. These discoveries play a vital role in drug discovery and design, as ligands with consistently low binding energies are more likely to demonstrate strong and dependable binding profiles, positioning them as promising candidates for additional experimental validation and drug development endeavours. Furthermore, these findings highlight the success of the MM-PBSA method in capturing the intricate molecular interactions and offering important insights into the energetics of ligand-receptor binding throughout MD simulations.

**TABLE 9:** MM-PBSA of MM-GBSA binding free energies estimated in kJ/mol.

Energy (kJ/mol)	**9**	**27**	41	**68**	74	85	**99**	180	**MTX**	**PTX**
**MMGBSA**										
∆E_vdw_	−197.32	−182.26	−270.6	−179.62	−290.58	−210.58	−220.6	−230.6	−192.23	−185.23
∆E_elec_	−551.54	−543.39	−478	−556.85	−498.02	−403.02	−439.1	−467.0	−537.41	−511.71
∆E_solv_	654.78	641.93	585.32	645.36	532.23	525.39	517.23	505.32	654.95	626.41
∆E_SASA_	−22.18	−21.85	−21.89	−21.1	−22.89	−24.849	−23.89	−21.89	−21.07	−20.96
∆G_binding_	−116.26	−105.57	−185.17	−112.21	−279.26	−113.06	−166.36	−214.17	−95.76	−91.49

Lowest free binding energy ligand numbers is shown in bold, vdw van der Waals, elect electrostatic, solv polar solvation, SASA solvent-accessible surface area.

## Conclusion

In summary, the 2D-QSAR model was generated to retrieve the potential of the Zinc15 compound library as DHFR inhibitors. QSAR modelling, virtual screening and deep learning-based ADMET resulted in screening the top hits compounds. The FDA-approved drugs, MTX & PTX, were considered as standard drugs to which *in silico* findings could be compared and examined. The eight top hits labelled as **09, 27, 41, 68, 74, 85, 99, and 180** were chosen with binding affinity ≤ −11.0 kal/mol and pIC_50_ ranges from 5.85 to 7.64. The ADMET parameters, Lipinski rule of five and clinical toxicity matrices were considered to ensure drug likeliness features and safety of the compounds under investigation. The DFT was used to optimize the investigating compound’s stability and physicochemical parameters. The top hits exhibited electrophilicity values ranging from 6.24 eV to 10.22 eV, representing the electron acceptor property required for varying receptor interactions. Further, the molecular docking and dynamics studies revealed significantly higher binding affinity scores and stability of protein-ligand complex over 100 ns simulation compared to reference drugs MTX and PTX, endorsing their reliable anticancer activity. The study provided considerable insight for developing selected compounds as novel anticancer drugs in the future. In short, the findings of the current study identified top hits that could prove themselves as an effective treatment strategy for DHFR inhibition leading to treat and manage cancer malignancies. These findings will assist researchers to develop newer leads without consuming much time and financial resources so further experimental studies are also recommended for future prospects and to explore its newer aspects. In view of the current study, there is always a platform for future studies focusing on the incorporation of a more extensive and diverse set of experimental data to apply the approach developed here to *in-vivo* for the sake of developing a generalized model.

## Data Availability

The original contributions presented in the study are included in the article/Supplementary Material, further inquiries can be directed to the corresponding authors.
