# Supplementary material for: Revisiting methotrexate and phototrexate Zinc15 library-based derivatives using deep learning in-silico drug design approach
Source: Front Chem. 2024 Mar 21;12:1380266. doi: 10.3389/fchem.2024.1380266 (PMC10991842; doi:10.3389/fchem.2024.1380266)
Supplement: Supplementary file 1 [file DataSheet1.pdf]

Revisiting Methotrexate and Phototrexate Zinc15 Library-Based Derivatives Using Deep Learning *In-Silico* Drug Design Approach

Farhan Siddique[1,2*], Ahmar Anwaar[3], Maryam Bashir[2,4], Sumaira Nadeem[5], Ravi Rawat[6], Volkan Eyupoglu[7], Samina Afzal[2], Mehvish Bibi[2], Yousef A. Bin Jardan [8], Mohammed Bourhia[9*]

Supplementary Information

Table of Contents

Table S1 2D chemical structures, SMILES (Simplified Molecular Input Line Entry System) name. Zinc ID of studied PTX and MTX library........ 2

Table S2 Ligands used for screening of top hit compounds using AD4 and Autodock vina........ 50

***Figure S*1:** Deep learning based QSAR model for method validation and correlation analysis. 54

***Figure S*2**: Deep learning based QSAR *model* for absorption HIA prediction. 55

***Figure S*3:** Deep learning based QSAR *model* for absorption Caco2 prediction........ 55

***Figure S*4**: Deep learning based QSAR *model* for absorption Pgp prediction. 56

***Figure S*5:** Deep learning based QSAR *model* for bioavailability prediction........ 56

Figure S6: Deep learning based QSAR *model* for excretion clearance prediction. 57

***Figure S*7:** Deep learning based QSAR *model* for distribution prediction........ 57

***Figure S*8:** Deep learning based QSAR *model* for distribution PPBR prediction........ 58

***Figure S*9**: Deep learning based QSAR *model* for excretion Half-life prediction........ 58

Figure *S*10: Deep learning based QSAR *model* for metabolism by CYP2C9 prediction........ 59

***Figure S*11:** Deep learning based QSAR *model* for metabolism by CYP2C19 prediction. 59

***Figure S*12:** Deep learning based QSAR *model* for metabolism by CYP2D6 prediction........ 60

***Figure S*13:** Deep learning based QSAR *model* for metabolism by CYP3A4 prediction........ 60

***Figure S*14**: Deep learning based QSAR *model* for metabolism by CYP1A2 prediction........ 61

***Figure S*15**: Deep learning based QSAR *model* for lipophilicity prediction........ 61

***Figure S*16**: Deep learning based QSAR model for solubility prediction........ 62

Figure S17*:* Deep learning based QSAR *model* for clinical toxicity prediction. 62

Table S3 Optimized XYZ cartesian data for all the top-hit lead compounds used in the study. 63

Figure S18: The snapshots of investigated ligands- and reference-protein complexes at 0 ns. 77

Figure S19: The snapshots of ligand-27 protein complex at 25, 50, 75 and 100 ns. 78

Figure S20: The snapshots of ligand-41 protein complex at 25, 50, 75 and 100 ns. 79

Figure S21: The snapshots of ligand-68 protein complex at 25, 50, 75 and 100 ns. 80

Figure S23: The snapshots of ligand-85 protein complex at 25, 50, 75 and 100 ns. 82

Figure S24: The snapshots of ligand-99 protein complex at 25, 50, 75 and 100 ns. 83
Figure S25: The snapshots of ligand-185 protein complex at 25, 50, 75 and 100 ns. 84
Figure S26: The snapshots of ligand-185 protein complex at 25, 50, 75 and 100 ns. 85
Figure S27: The snapshots of MTX-protein complex at 25, 50, 75 and 100 ns. 86
Figure S28: The snapshots of PTX-protein complex at 25, 50, 75 and 100 ns. 87

Table S1 2D chemical structures, SMILES (Simplified Molecular Input Line Entry System) name. Zinc ID of studied PTX and MTX library

Lig. Code	Chemical Structure 2D (Chem Draw)	SMILES	Zinc ID
1	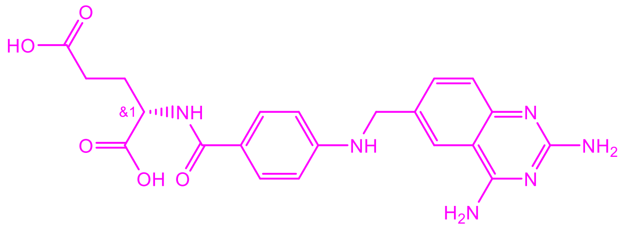	NC1=NC(N)=C2C=C(C=CC2=N1)CNC3=CC=C(C=C3)C(N[C@H](C(O)=O)CCC(O)=O)=O	ZINC0000016074 21
2	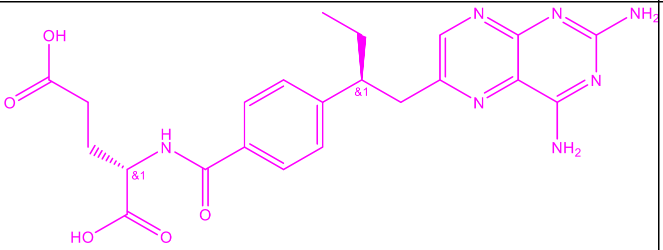	CC[C@H](C1=CC=C(C=C1)C(N[C@H](C(O)=O)CCC(O)=O)=O)CC2=CN=C3N=C(N=C(C3=N2)N)N	ZINC0000016187 02
3	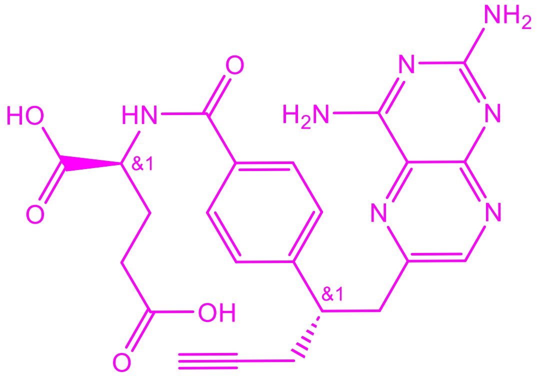	C#CC[C@H](C1=CC=C(C=C1)C(N[C@H](C(O)=O)CCC(O)=O)=O)CC2=CN=C3N=C(N=C(C3=N2)N)N	ZINC0000015361 09
4	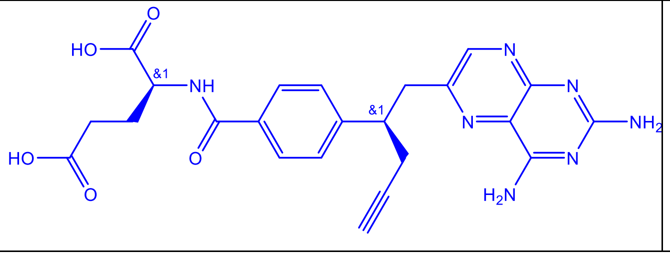	C#CC[C@@H](C1=CC=C(C=C1)C(N[C@H](C(O)=O)CCC(O)=O)=O)CC2=CN=C3N=C(N=C(C3=N2)N)N	ZINC0000116169 25

5	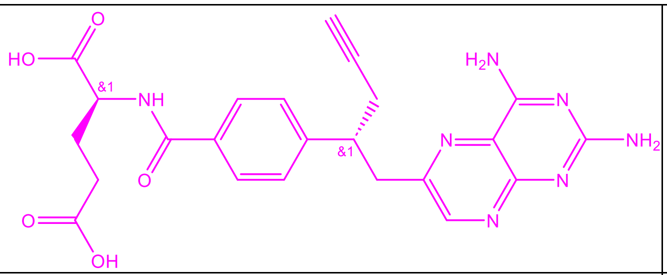	C#CC[C@H](C1=CC=C(C=C1)C(N[C@@H](C(O)=O)CCC(O)=O)=O)CC2=CN=C3N=C(N=C(C3=N2)N)N	ZINC000011616926
6	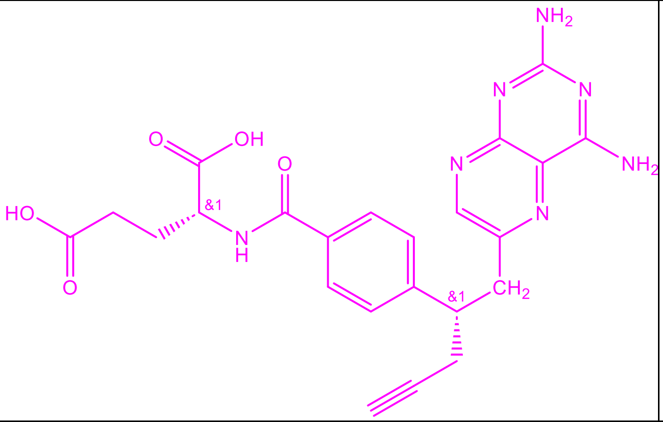	C#CC[C@H](C1=CC=C(C=C1)C(N[C@@H](C(O)=O)CCC(O)=O)=O)CC2=CN=C3N=C(N=C(C3=N2)N)N	ZINC000011616927
7	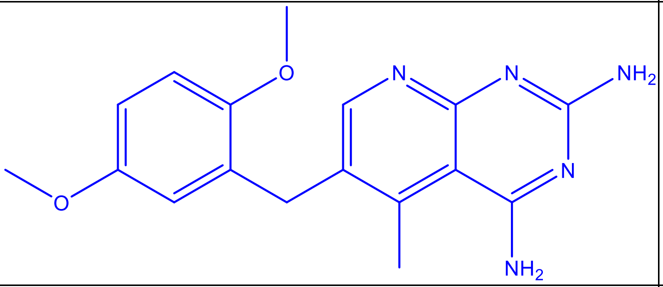	COC1=CC=C(C(CC2=CN=C3N=C(N=C(C3=C2C)N)N)=C1)OC	ZINC000000000640
8	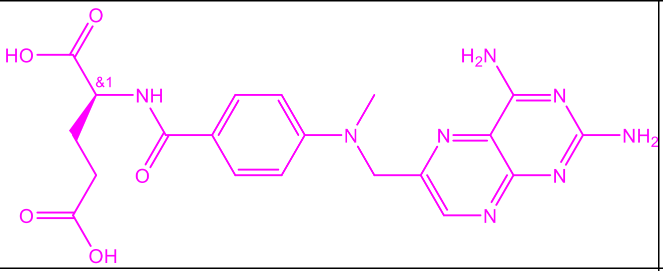	CN(C1=CC=C(C=C1)C(N[C@H](C(O)=O)CCC(O)=O)=O)CC2=CN=C3N=C(N=C(C3=N2)N)N	ZINC000001529323
9	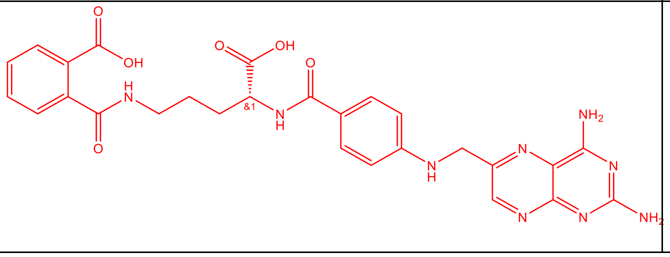	NC1=NC(N)=C2N=C(C=NC2=N1)CNC3=CC=C(C=C3)C(N[C@@H](C(O)=O)CCCNC(C4=CC=CC=C4C(O)=O)=O)=O	ZINC000003807186

10	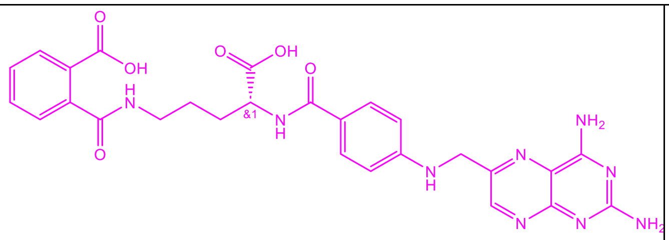	NC1=NC(N)=C2N=C(C=NC2=N1)CNC3=CC=C(C=C3)C(N[C@@H](C(O)=O)CCCNC(C4=CC=CC=C4C(O)=O)=O)=O	ZINC000005545634
11	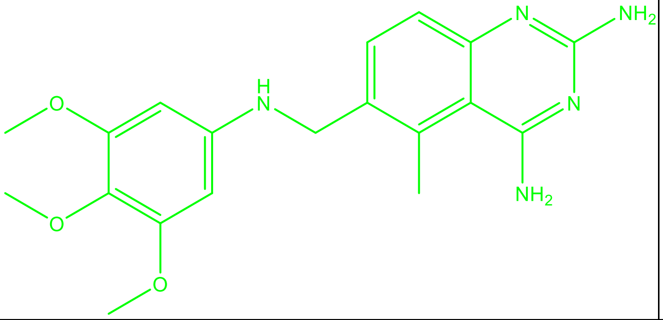	COC1=CC(NCC2=CC=C3N=C(N=C(C3=C2C)N)N)=CC(OC)=C1OC	ZINC000000598852
12	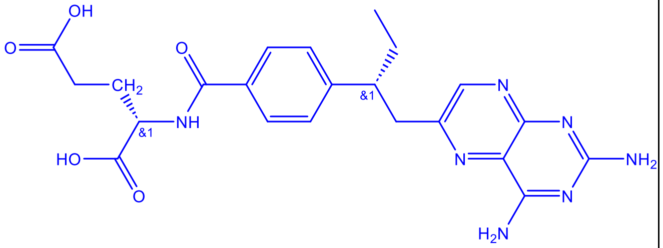	CC[C@@H](C1=CC=C(C=C1)C(N[C@H](C(O)=O)CCC(O)=O)=O)CC2=CN=C3N=C(N=C(C3=N2)N)N	ZINC000001618703
13	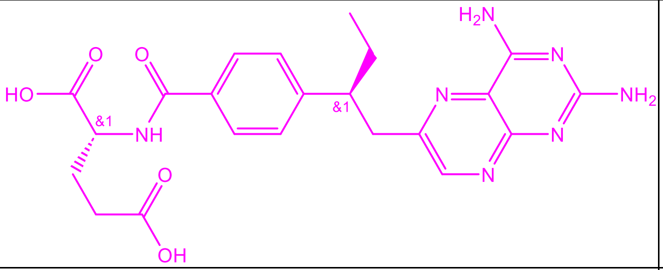	CC[C@H](C1=CC=C(C=C1)C(N[C@@H](C(O)=O)CCC(O)=O)=O)CC2=CN=C3N=C(N=C(C3=N2)N)N	ZINC000001618704
14	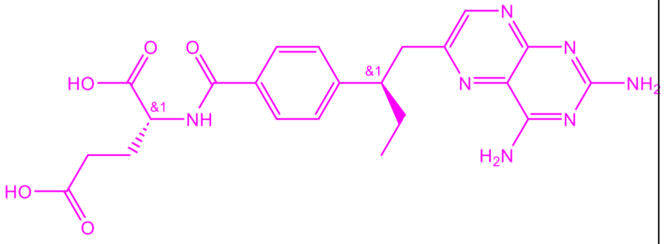	CC[C@@H](C1=CC=C(C=C1)C(N[C@@H](C(O)=O)CCC(O)=O)=O)CC2=CN=C3N=C(N=C(C3=N2)N)N	ZINC000001618705
15	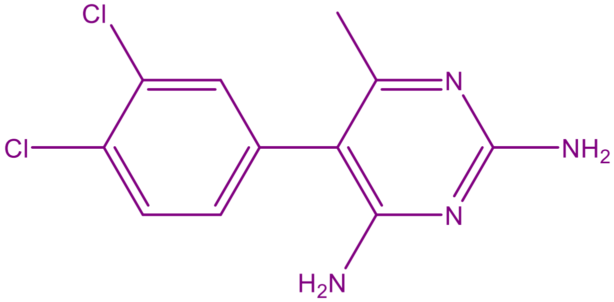	CC1=NC(N)=NC(N)=C1C2=CC=C(C(Cl)=C2)Cl	ZINC000000001723

16	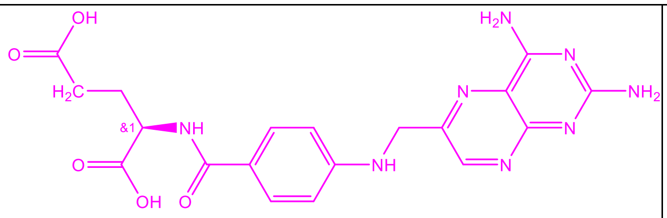	NC1=NC(N)=C2N=C(C=NC2=N1)CNC3=CC=C(C=C3)C(N[C@@H](C(O)=O)CCC(O)=O)=O	ZINC000002036915
17	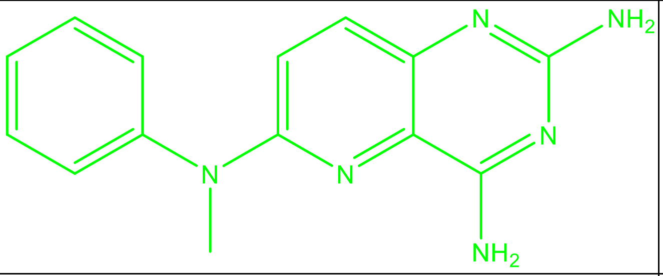	CN(C1=CC=C2N=C(N=C(C2=N1)N)N)C3=CC=CC=C3	ZINC000003814936
18	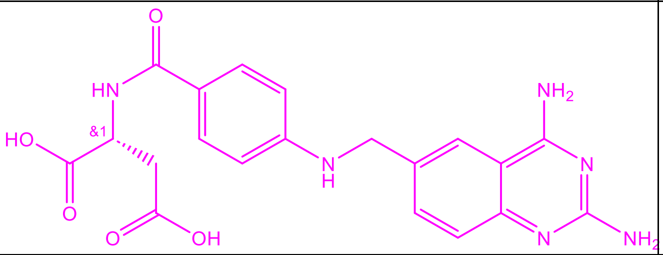	NC1=NC(N)=C2C=C(C=CC2=N1)CNC3=CC=C(C=C3)C(N[C@@H](C(O)=O)CC(O)=O)=O	ZINC000001703901
19	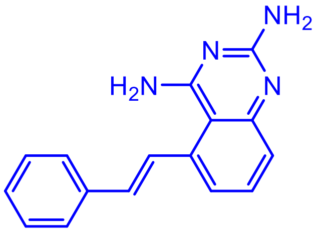	NC1=NC(N)=C2C(/C=C/C3=CC=CC=C3)=CC=CC2=N1	ZINC000005974460
20	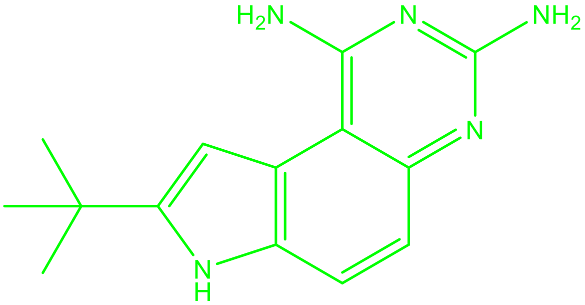	CC(C)(C1=CC2=C(N1)C=CC3=NC(N)=NC(N)=C23)C	ZINC000005881950
21	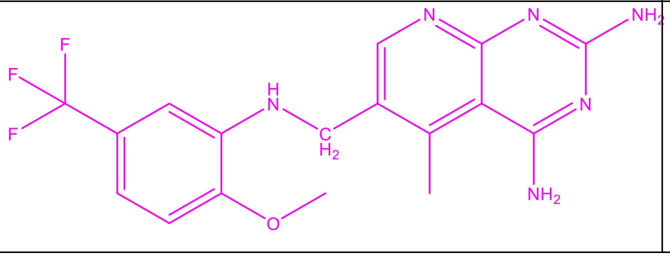	COC1=CC=C(C=C1NCC2=CN=C3N=C(N=C(C3=C2C)N)N)C(F)(F)F	ZINC000001489187

22	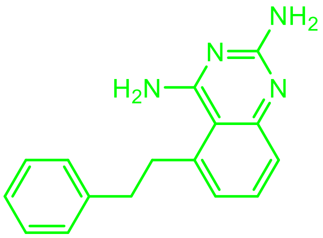	NC1=NC(N)=C2C(CCC3=CC=CC=C3)=CC=CC2=N1	ZINC000013740535
23	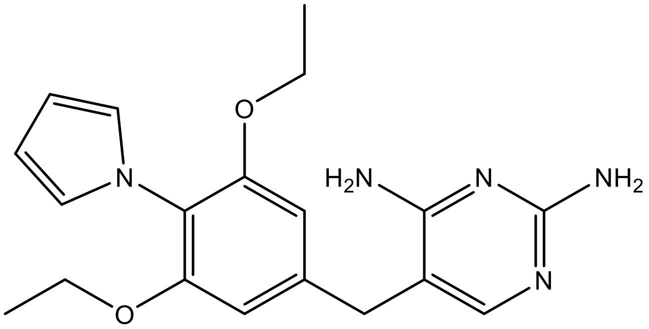	CCOC1=CC(CC2=CN=C(N=C2N)N)=CC(OCC)=C1N3C=CC=C3	ZINC000000598590
24	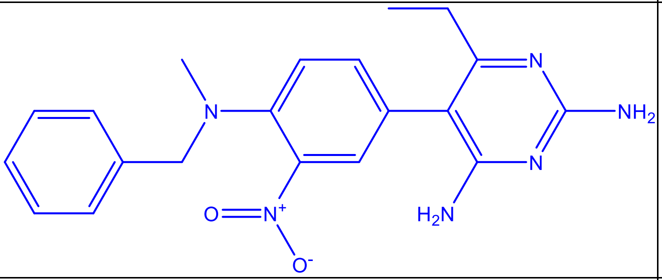	CCC1=NC(N)=NC(N)=C1C2=CC=C(C([N+]([O-])=O)=C2)N(CC3=CC=CC=C3)C	ZINC000003777839
25	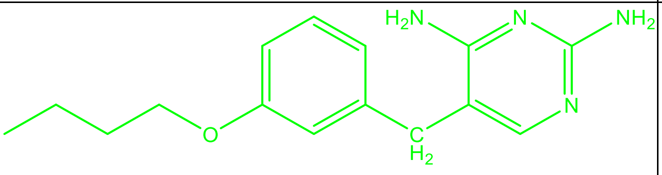	CCCCOC1=CC=CC(CC2=CN=C(N=C2N)N)=C1	ZINC000013726735
26	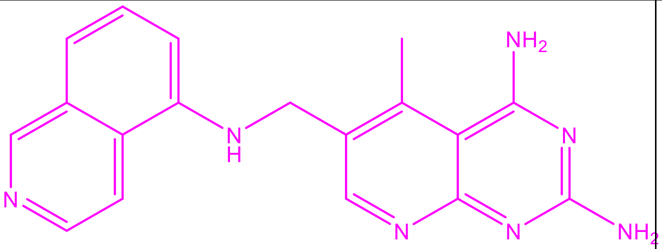	CC1=C(C=NC2=NC(N)=NC(N)=C12)CNC3=CC=CC4=CN=CC=C34	ZINC000005891520
27	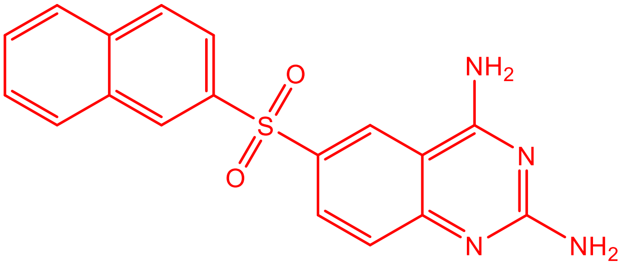	NC1=NC(N)=C2C=C(C=CC2=N1)S(=O)(C3=CC=C4C=CC=CC4=C3)=O	ZINC000006118800

28	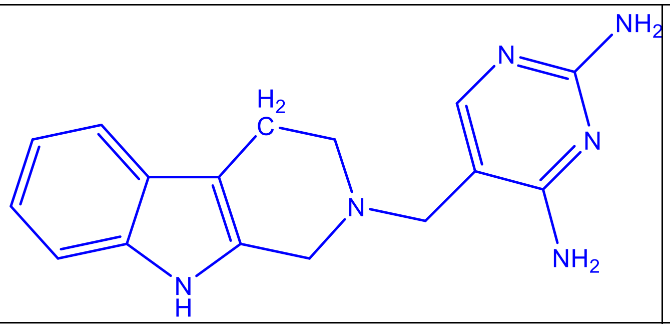	NC1=NC=C(C(N)=N1)CN2CCC3=C(C2)NC4=CC=CC=C34	ZINC000013520554
29	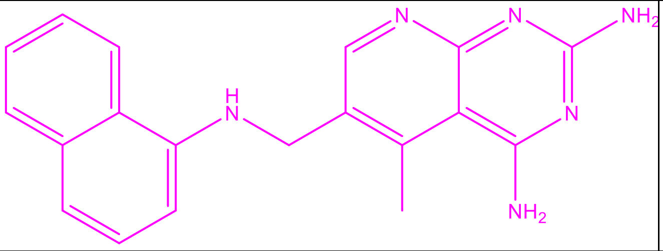	CC1=C(C=NC2=NC(N)=NC(N)=C12)CNC3=CC=CC4=CC=CC=C34	ZINC000003814957
30	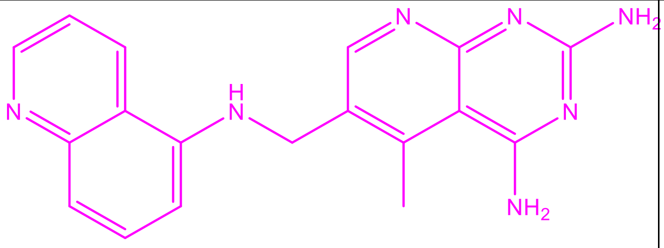	CC1=C(C=NC2=NC(N)=NC(N)=C12)CNC3=CC=CC4=NC=CC=C34	ZINC000012503486
31	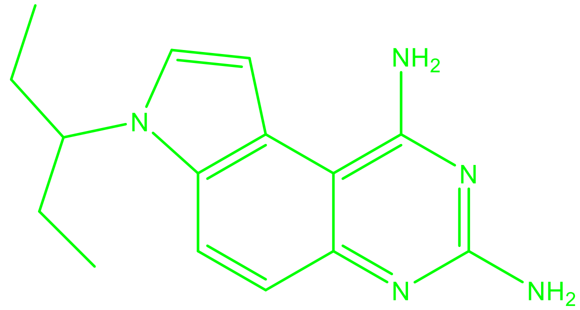	CCC(N1C=CC2=C1C=CC3=NC(N)=NC(N)=C32)CC	ZINC000000015885
32	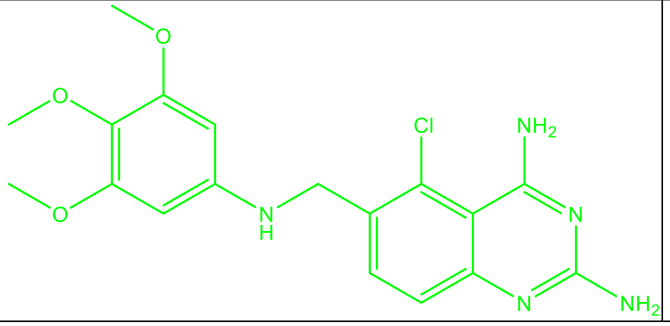	COC1=CC(NCC2=CC=C3N=C(N=C(C3=C2Cl)N)N)=CC(OC)=C1OC	ZINC000003814857

33	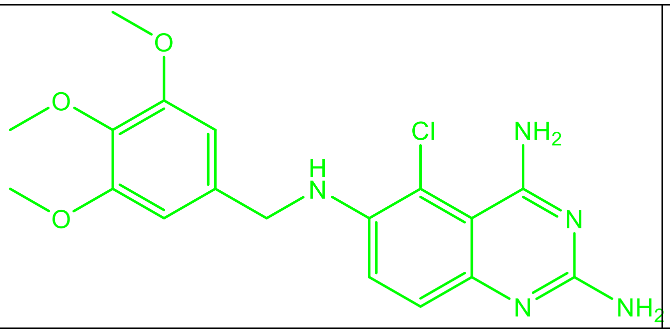	COC1=CC(CNC2=CC=C3N=C(N=C(C3=C2Cl)N)N)=CC(OC)=C1OC	ZINC000003814852
34	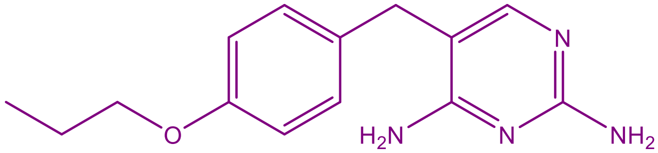	CCCOC1=CC=C(C=C1)CC2=CN=C(N=C2N)N	ZINC000003157274
35	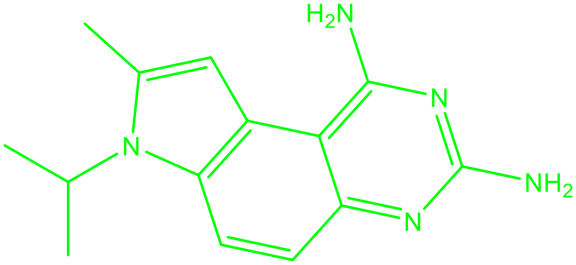	CC1=CC2=C(N1C(C)C)C=CC3=NC(N)=NC(N)=C23	ZINC000005892288
36	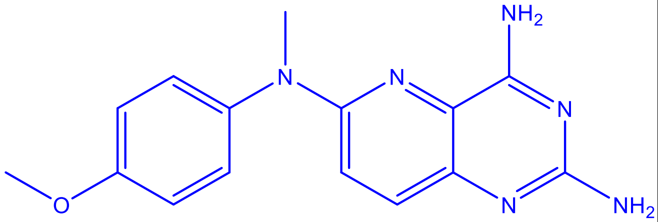	COC1=CC=C(C=C1)N(C2=CC=C3N=C(N=C(C3=N2)N)N)C	ZINC000003814937
37	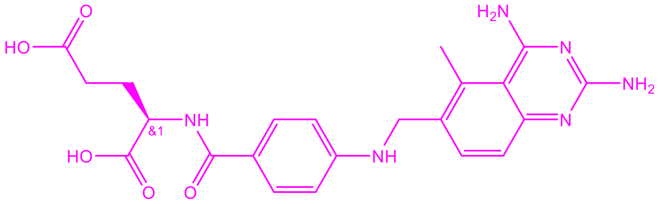	CC1=C(C=CC2=NC(N)=NC(N)=C12)CNC3=CC=C(C=C3)C(N[C@@H](C(O)=O)CCC(O)=O)=O	ZINC000021289010
38	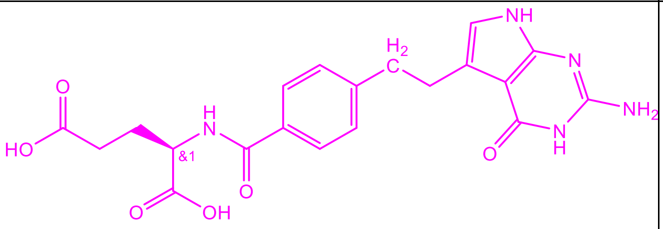	NC1=NC2=C(C(N1)=O)C(CCC3=CC=C(C=C3)C(N[C@@H](C(O)=O)CCC(O)=O)=O)=CN2	ZINC000001540998

39	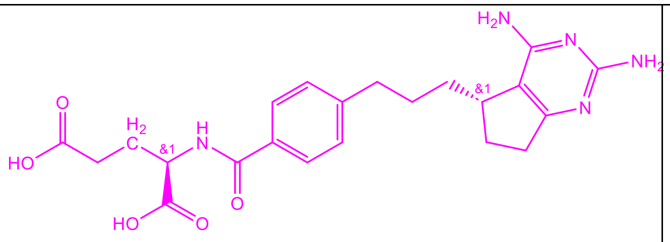	NC1=NC2=C([C@H](CC2)CCCC3=CC=C(C=C3)C(N[C@@H](C(O)=O)CCC(O)=O)=O)C(N)=N1	ZINC000003789075
40	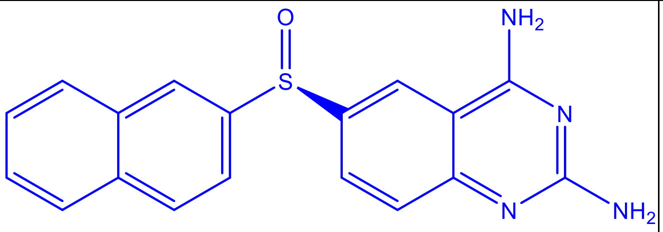	NC1=NC(N)=C2C=C([S@@](C3=CC=C4C=CC=CC4=C3)=O)C=CC2=N1	ZINC000005386242
41	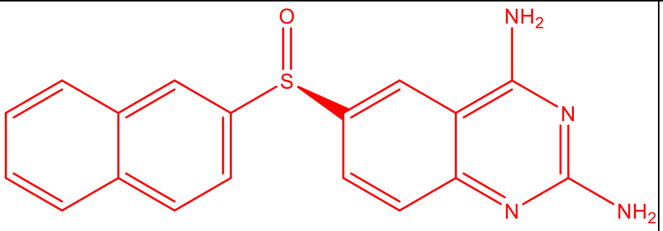	NC1=NC(N)=C2C=C([S@@](C3=CC=C4C=CC=CC4=C3)=O)C=CC2=N1	ZINC000005647485
42	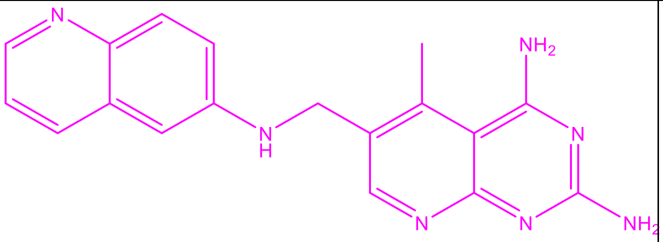	CC1=C(C=NC2=NC(N)=NC(N)=C12)CNC3=CC=C4N=CC=CC4=C3	ZINC000005891502
43	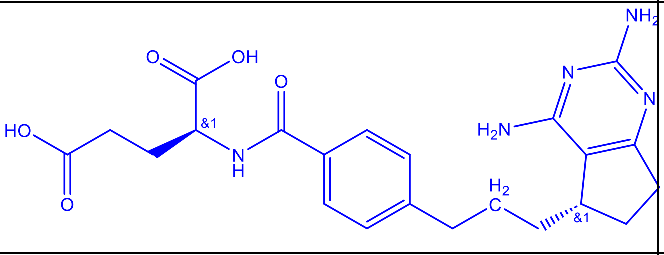	NC1=NC2=C([C@H](CC2)CCCC3=CC=C(C=C3)C(N[C@H](C(O)=O)CCC(O)=O)=O)C(N)=N1	ZINC000011686992
44	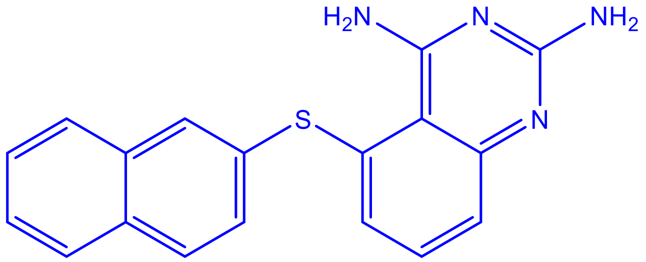	NC1=NC(N)=C2C(C=CC=C2SC3=CC=C4C=CC=CC4=C3)=N1	ZINC000027657972

45	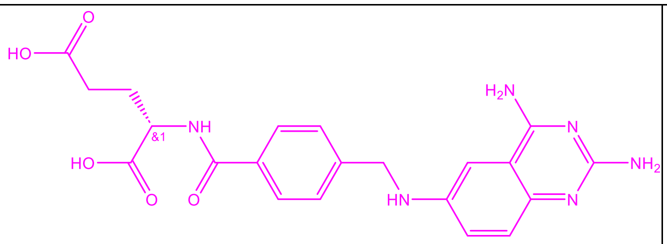	NC1=NC(N)=C2C=C(C=CC2=N1)NCC3=CC=C(C=C3)C(N[C@H](C(O)=O)CCC(O)=O)=O	ZINC000017106148
46	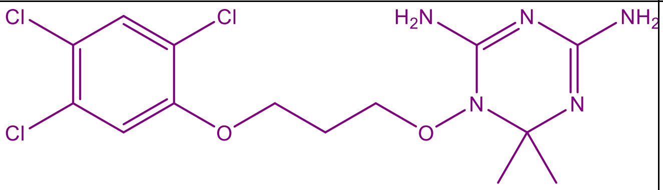	CC1(N=C(N=C(N1OCCCOC2=CC(Cl)=C(C=C2Cl)Cl)N)N)C	ZINC000003581056
47	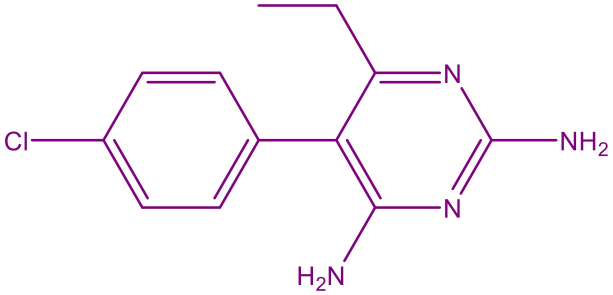	CCC1=NC(N)=NC(N)=C1C2=CC=C(C=C2)Cl	ZINC000000057464
48	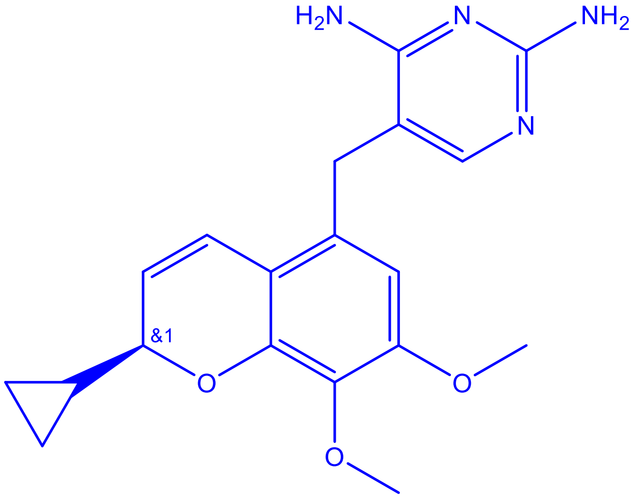	COC1=CC(CC2=CN=C(N=C2N)N)=C3C(O[C@H](C=C3)C4CC4)=C1OC	ZINC000001486728

49	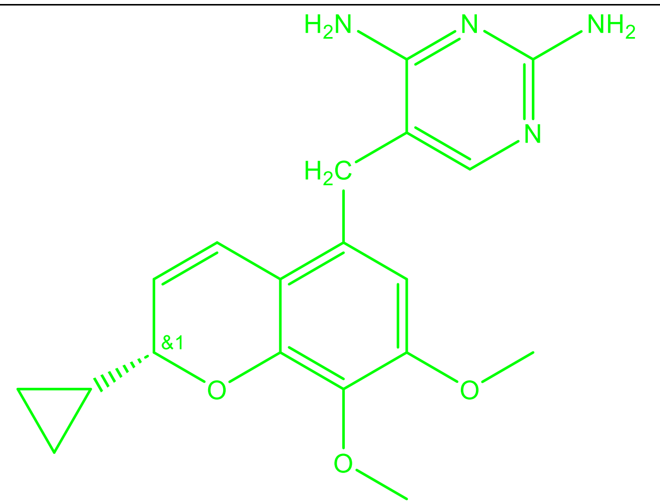	COC1=CC(CC2=CN=C(N=C2N)N)=C3C(O[C@@H](C=C3)C4CC4)=C1OC	ZINC000003612862
50	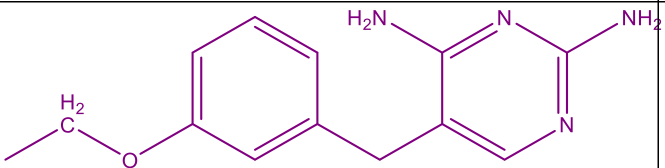	CCOC1=CC=CC(CC2=CN=C(N=C2N)N)=C1	ZINC000013801424
51	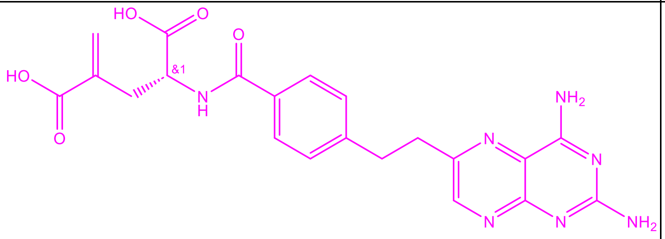	C=C(C(O)=O)C[C@H](C(O)=O)NC(C1=CC=C(C=C1)CCC2=CN=C3N=C(N=C(C3=N2)N)N)=O	ZINC000001542008
52	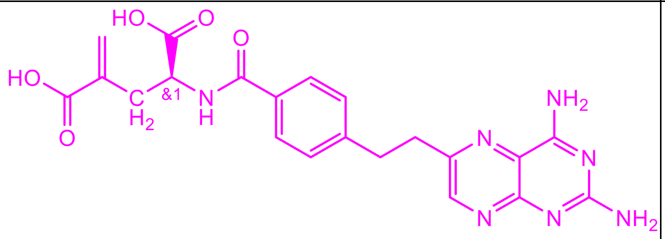	C=C(C(O)=O)C[C@@H](C(O)=O)NC(C1=CC=C(C=C1)CCC2=CN=C3N=C(N=C(C3=N2)N)N)=O	ZINC000013860914
53	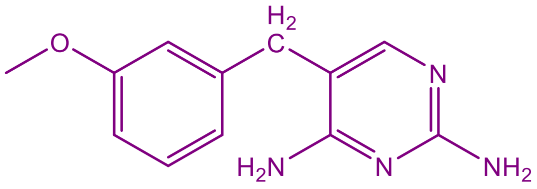	COC1=CC=CC(CC2=CN=C(N=C2N)N)=C1	ZINC000005115792

54	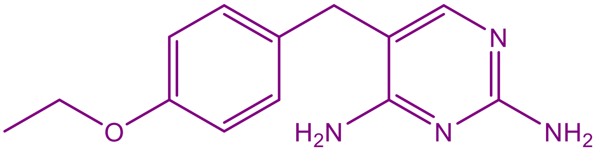	CCOC1=CC=C(C=C1)CC2=CN=C(N=C2N)N	ZINC000000289771
55	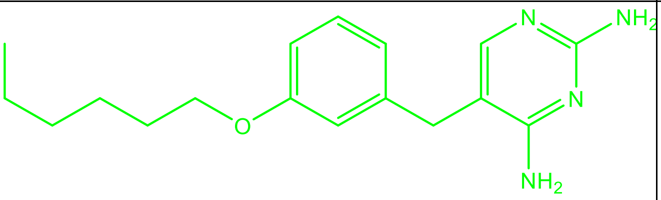	CCCCCCOC1=CC=CC(CC2=CN=C(N=C2N)N)=C1	ZINC000013726744
56	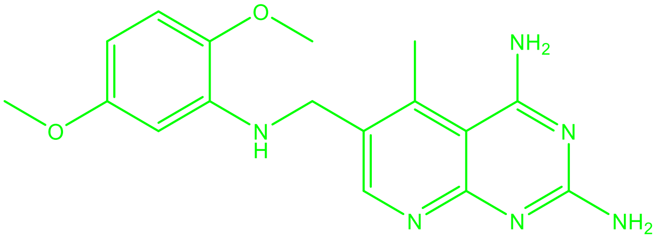	COC1=CC=C(C(NCC2=CN=C3N=C(N=C(C3=C2C)N)N)=C1)OC	ZINC000001996101
**57**	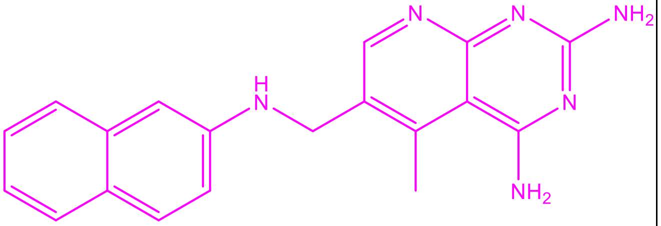	CC1=C(C=NC2=NC(N)=NC(N)=C12)CNC3=CC=C4C=CC=CC4=C3	ZINC000005891512
58	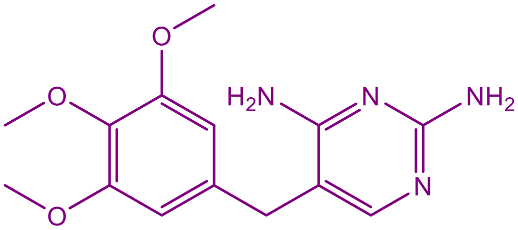	COC1=CC(CC2=CN=C(N=C2N)N)=CC(OC)=C1OC	ZINC000006627681
59	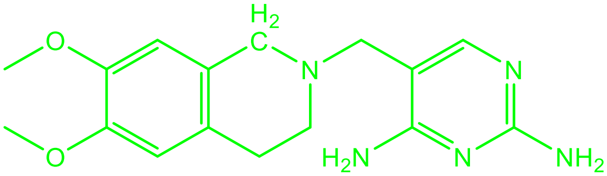	COC1=CC2=C(CN(CC2)CC3=CN=C(N=C3N)N)C=C1OC	ZINC000013520499

60	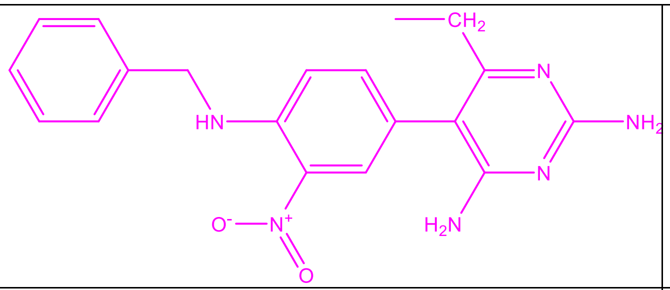	CCC1=NC(N)=NC(N)=C1C2=CC=C(C([N+]([O-])=O)=C2)NCC3=CC=CC=C3	ZINC000013765339
61	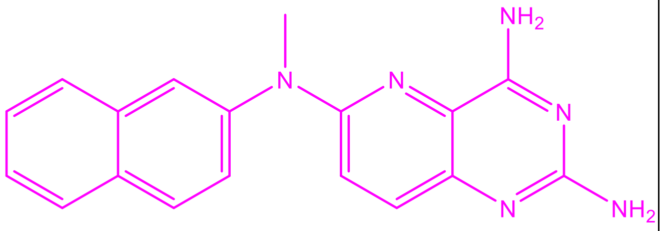	CN(C1=CC=C2N=C(N=C(C2=N1)N)N)C3=CC=C4C=CC=CC4=C3	ZINC000013781976
62	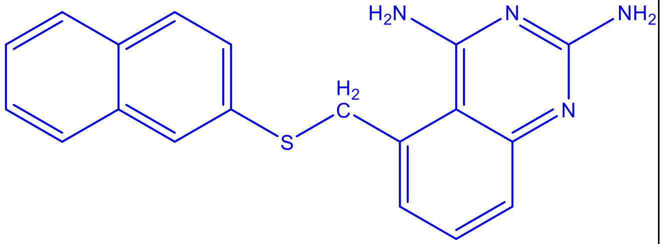	NC1=NC(N)=C2C(CSC3=CC=C4C=CC=CC4=C3)=CC=CC2=N1	ZINC000025970531
63	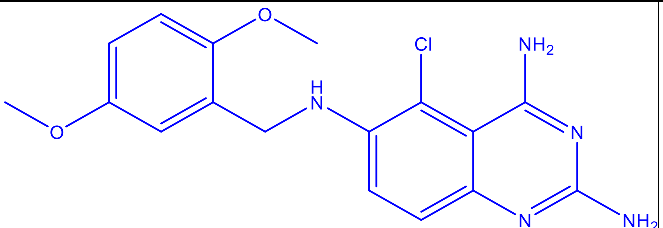	COC1=CC=C(C(CNC2=CC=C3N=C(N=C(C3=C2Cl)N)N)=C1)OC	ZINC000003814851
64	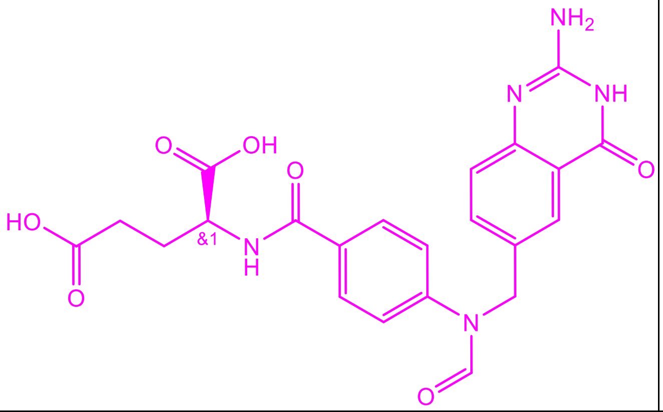	NC1=NC2=CC=C(C=C2C(N1)=O)CN(C3=CC=C(C=C3)C(N[C@H](C(O)=O)CCC(O)=O)=O)C=O	ZINC000002004188
65	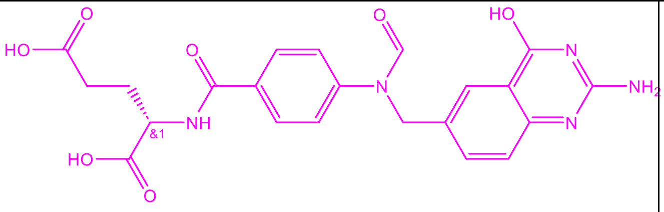	NC1=NC(O)=C2C=C(C=CC2=N1)CN(C3=CC=C(C=C3)C(N[C@H](C(O)=O)CCC(O)=O)=O)C=O	ZINC000005373985

66	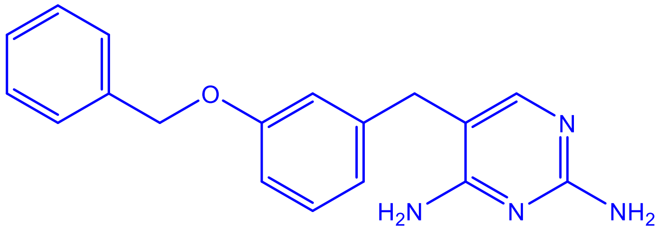	NC1=NC=C(C(N)=N1)CC2=CC=CC(OCC3=CC=CC=C3)=C2	ZINC000013532872
67	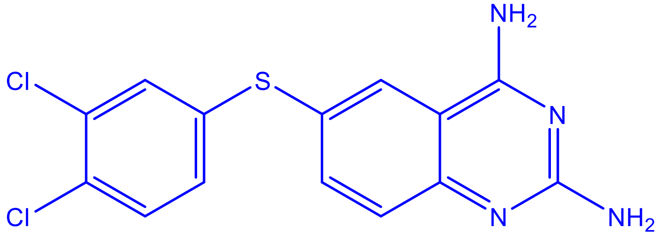	NC1=NC(N)=C2C=C(C=CC2=N1)SC3=CC=C(C(Cl)=C3)Cl	ZINC000025968813
68	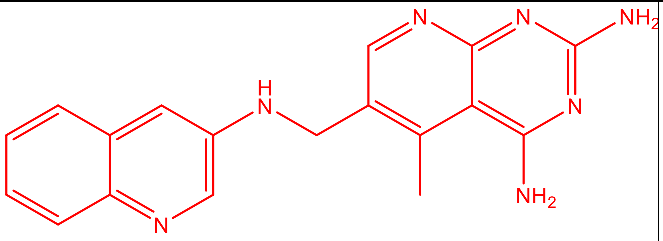	CC1=C(C=NC2=NC(N)=NC(N)=C12)CNC3=CN=C4C=CC=CC4=C3	ZINC000005891475
69	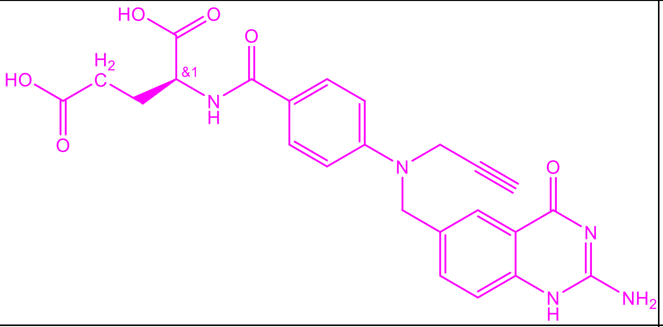	C#CCN(C1=CC=C(C=C1)C(N[C@H](C(O)=O)CCC(O)=O)=O)CC2=CC=C3NC(N)=NC(C3=C2)=O	ZINC000008655373
70	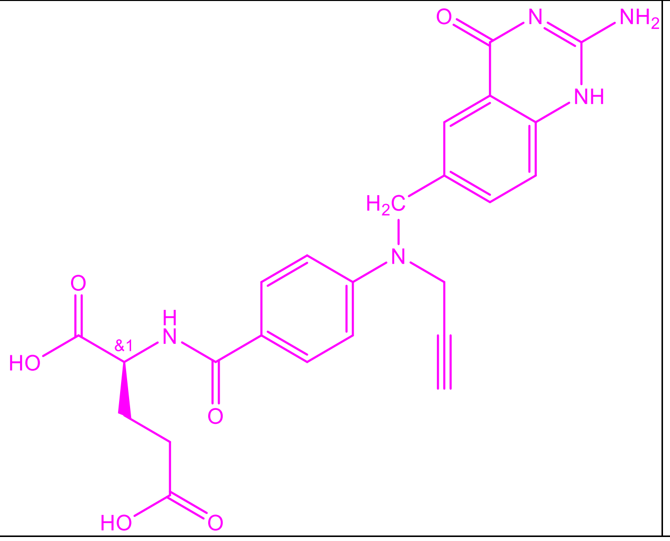	C#CCN(C1=CC=C(C=C1)C(N[C@H](C(O)=O)CCC(O)=O)=O)CC2=CC=C3NC(N)=NC(C3=C2)=O	ZINC000008655374

71	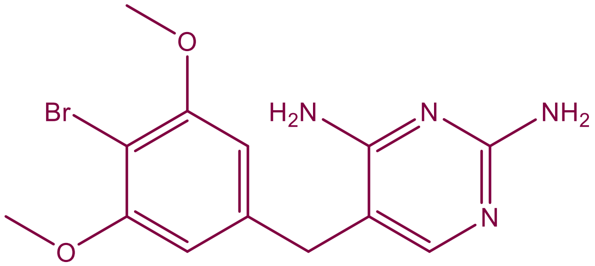	COC1=CC(CC2=CN=C(N =C2N)N)=CC(OC)=C1Br	ZINC000 00000582 4
72	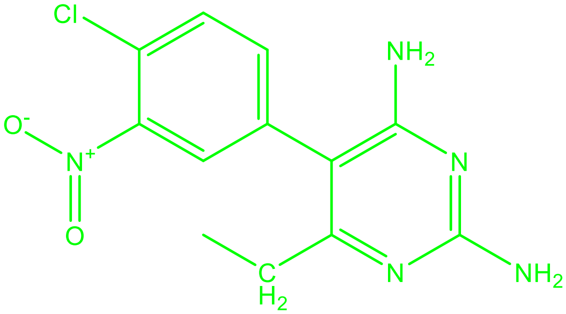	CCC1=NC(N)=NC(N)=C1 C2=CC=C(C([N+]([O-])=O)=C2)Cl	ZINC000 00156710 7
73	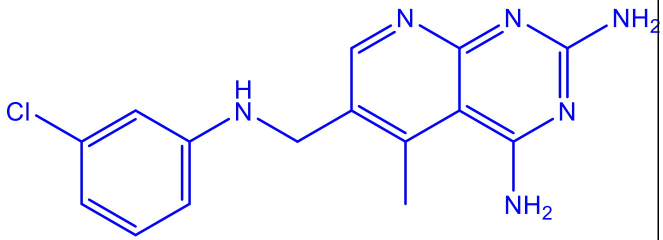	CC1=C(C=NC2=NC(N)=N C(N)=C12)CNC3=CC=CC (Cl)=C3	ZINC000 00381497 2
74	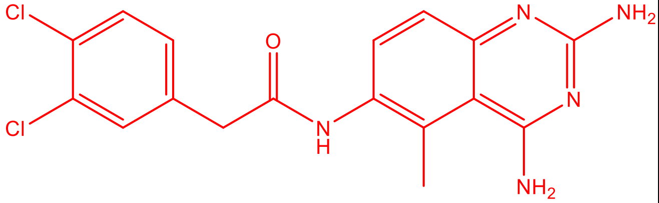	CC1=C(C=CC2=NC(N)=N C(N)=C12)NC(CC3=CC= C(C(Cl)=C3)Cl)=O	ZINC000 02596863 3
75	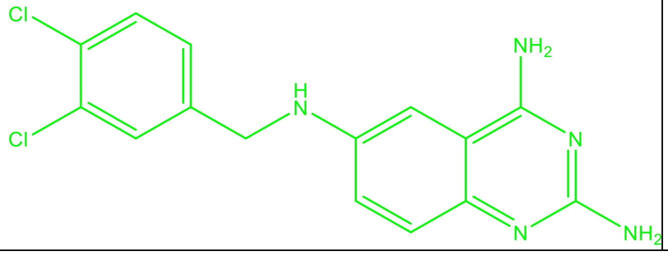	NC1=NC(N)=C2C=C(C=C C2=N1)NCC3=CC=C(C(Cl)=C3)Cl	ZINC000 01328509 5

76	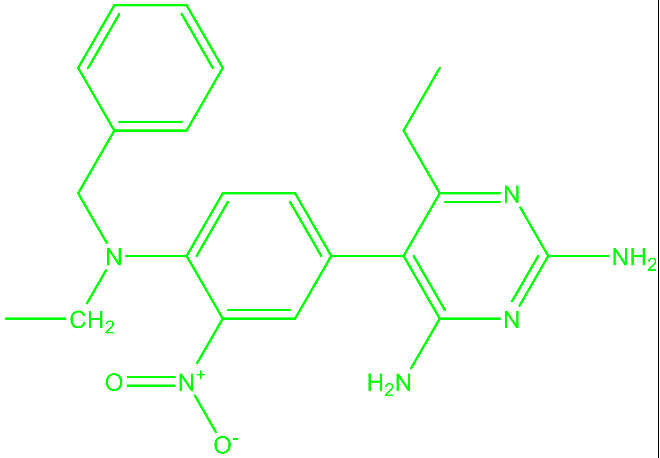	CCC1=NC(N)=NC(N)=C1C2=CC=C(C([N+]([O-])=O)=C2)N(CC3=CC=CC=C3)CC	ZINC000005115147
77	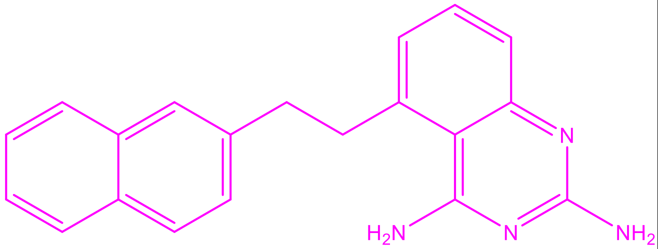	NC1=NC(N)=C2C(CCC3=CC=C4C=CC=CC4=C3)=CC=CC2=N1	ZINC000025970004
78	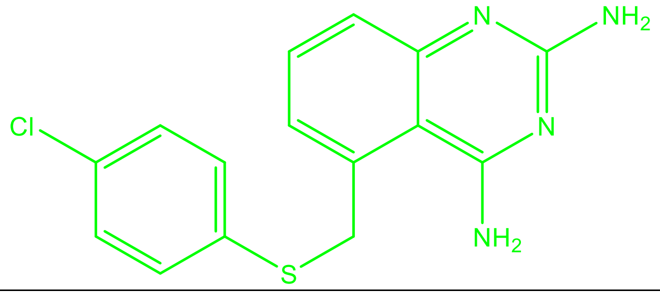	NC1=NC(N)=C2C(CSC3=CC=C(C=C3)Cl)=CC=CC2=N1	ZINC000025970010
79	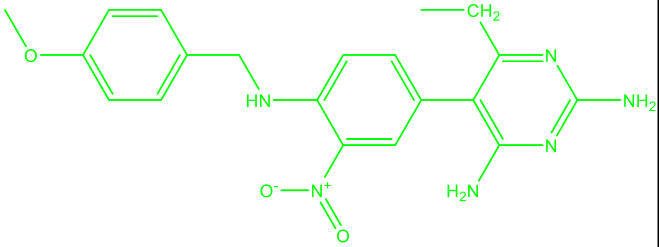	CCC1=NC(N)=NC(N)=C1C2=CC=C(C([N+]([O-])=O)=C2)NCC3=CC=C(C=C3)OC	ZINC000026503483
80	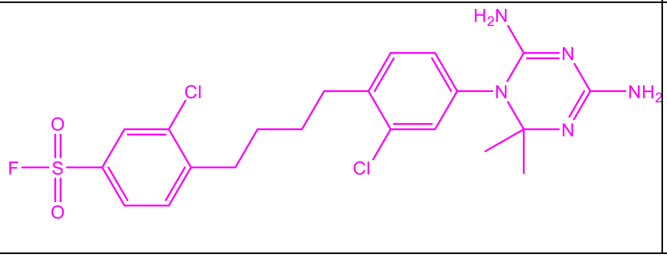	CC1(N=C(N=C(N1C2=CC=C(C(Cl)=C2)CCCCC3=CC=C(C=C3Cl)S(=O)(F)=O)N)N)C	ZINC000043706278

81	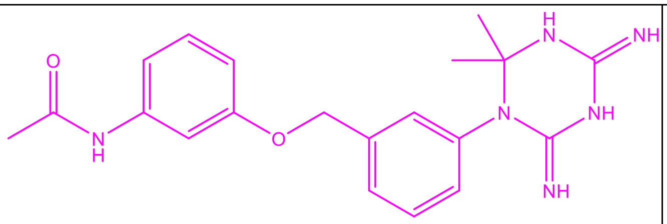	CC(NC1=CC=CC(OCC2=CC=CC(N3C(NC(NC(C)3C)=N)=N)=C2)=C1)=O	ZINC00008672818
82	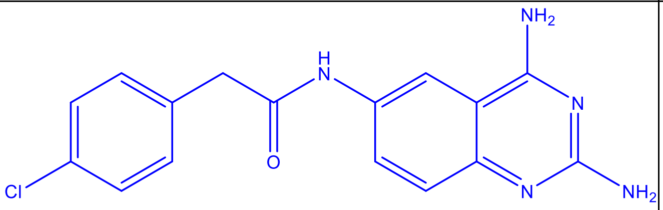	NC1=NC(N)=C2C=C(C=CC2=N1)NC(CC3=CC=C(C=C3)Cl)=O	ZINC00025968019
83	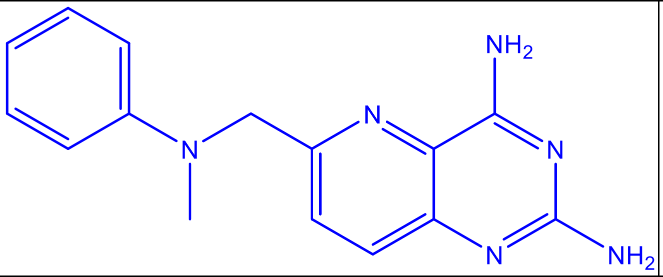	CN(C1=CC=CC=C1)CC2=CC=C3N=C(N=C(C3=N2)N)N	ZINC00003814928
84	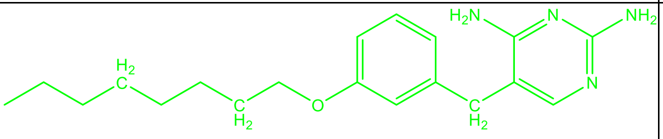	CCCCCCCCOC1=CC=CC(CC2=CN=C(N=C2N)N)=C1	ZINC00034801948
85	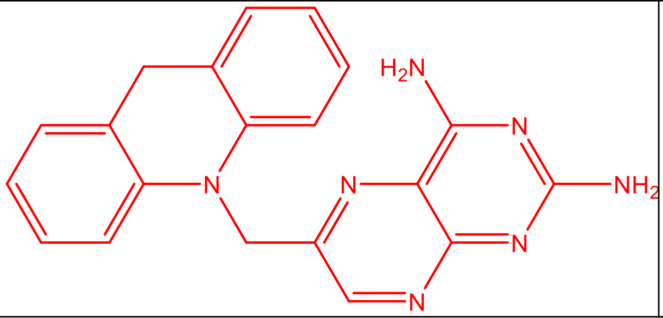	NC1=NC(N)=C2N=C(C=NC2=N1)CN3C4=CC=CC=C4CC5=CC=CC=C35	ZINC00003814848
86	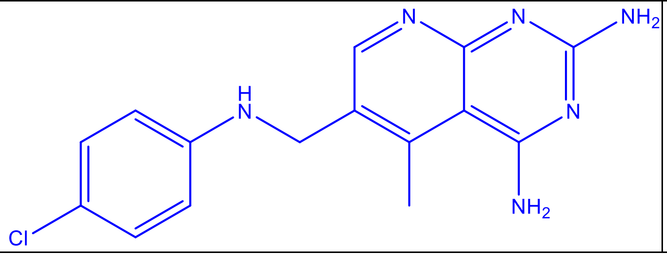	CC1=C(C=NC2=NC(N)=NC(N)=C12)CNC3=CC=C(C=C3)Cl	ZINC00003814973

87	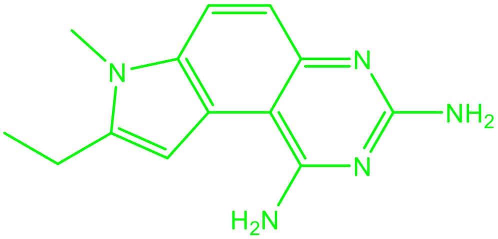	CCC1=CC2=C(N1C)C=CC3=NC(N)=NC(N)=C23	ZINC000005847670
88	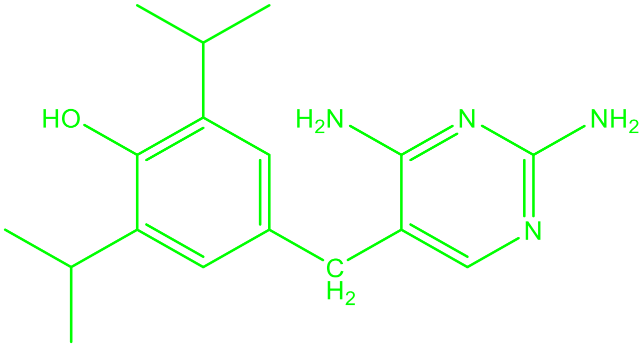	CC(C1=CC(CC2=CN=C(N=C2N)N)=CC(C(C)C)=C1O)C	ZINC000028264875
89	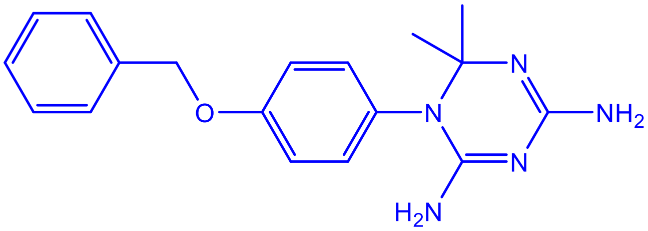	CC1(N=C(N=C(N1C2=CC=C(C=C2)OCC3=CC=CC=C3)N)N)C	ZINC000025969983
90	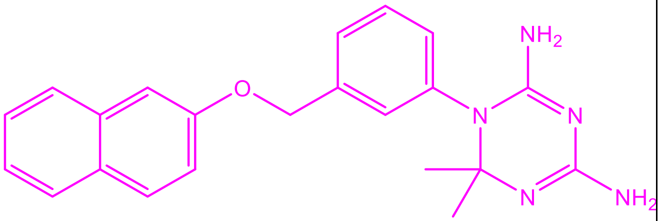	CC1(N=C(N=C(N1C2=CC=CC(COC3=CC=C4C=CC=CC4=C3)=C2)N)N)C	ZINC000029218398
91	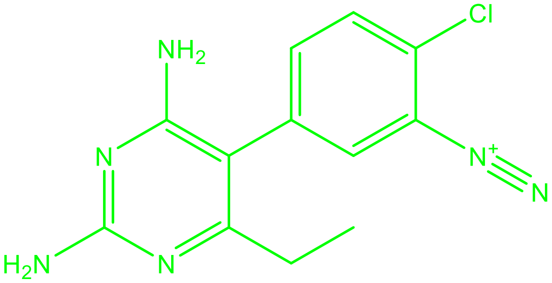	CCC1=NC(N)=NC(N)=C1C2=CC=C(C([N+]#N)=C2)Cl	ZINC000005386873
92	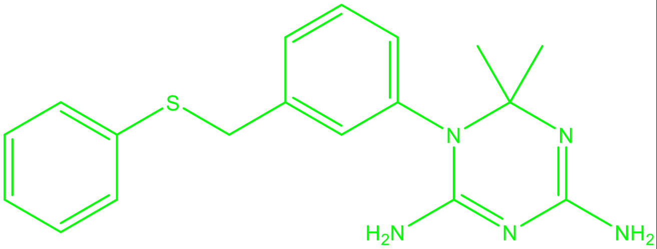	CC1(N=C(N=C(N1C2=CC=CC(CSC3=CC=CC=C3)=C2)N)N)C	ZINC000001587054

93	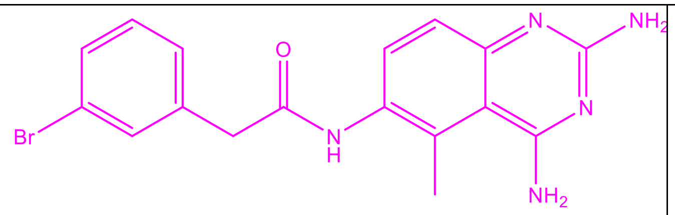	CC1=C(C=CC2=NC(N)=NC(N)=C12)NC(CC3=CC=CC(Br)=C3)=O	ZINC000025966456
94	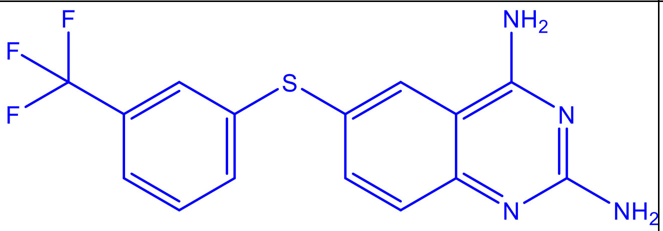	NC1=NC(N)=C2C=C(C=CC2=N1)SC3=CC=CC(C(F)(F)F)=C3	ZINC000013282326
95	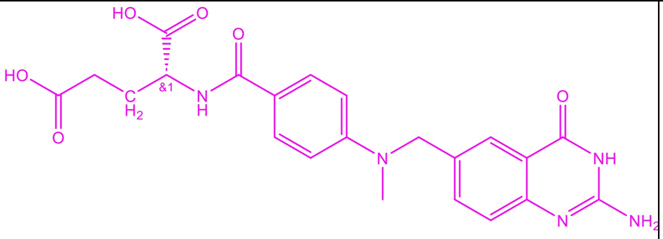	CN(C1=CC=C(C=C1)C(N[C@@H](C(O)=O)CCC(O)=O)=O)CC2=CC=C3N=C(NC(C3=C2)=O)N	ZINC000005011594
96	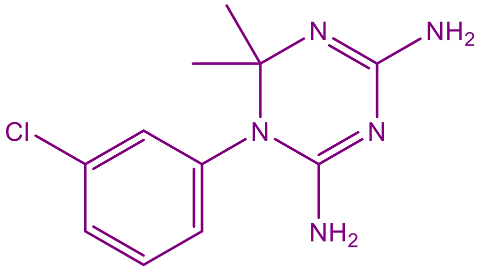	CC1(N=C(N=C(N1C2=CC=CC(Cl)=C2)N)N)C	ZINC000001666576
97	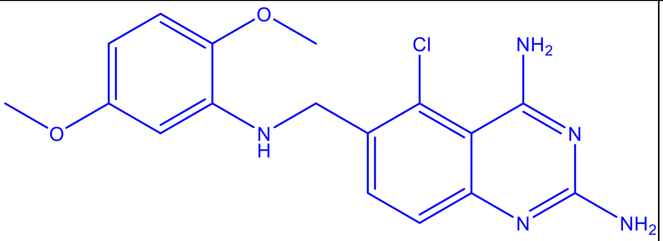	COC1=CC=C(C(NCC2=CC=C3N=C(N=C(C3=C2Cl)N)N)=C1)OC	ZINC000003814856
98	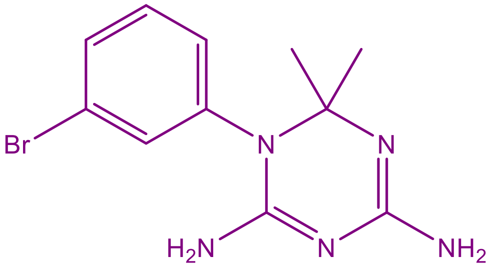	CC1(N=C(N=C(N1C2=CC=CC(Br)=C2)N)N)C	ZINC000001666578

99	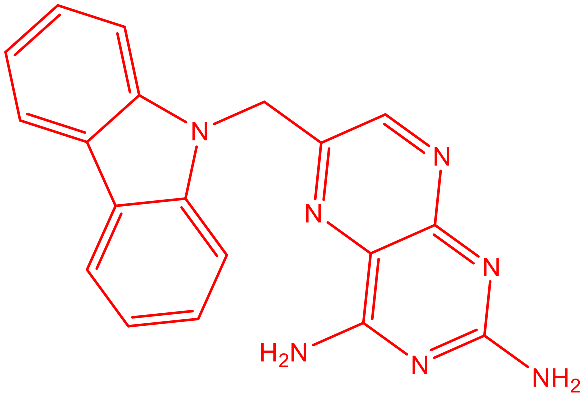	NC1=NC(N)=C2N=C(C=NC2=N1)CN3C4=CC=CC=C4C5=CC=CC=C35	ZINC000005939955
100	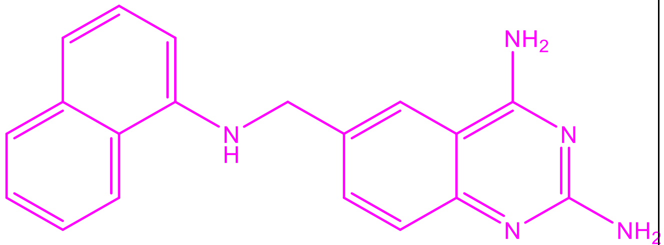	NC1=NC(N)=C2C=C(C=CC2=N1)CNC3=CC=CC4=CC=CC=C34	ZINC000005940023
101	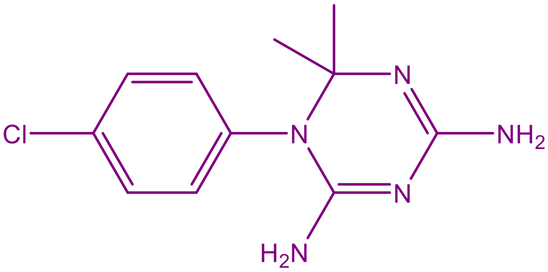	CC1(N=C(N=C(N1C2=CC=C(C=C2)Cl)N)N)C	ZINC000000001233
102	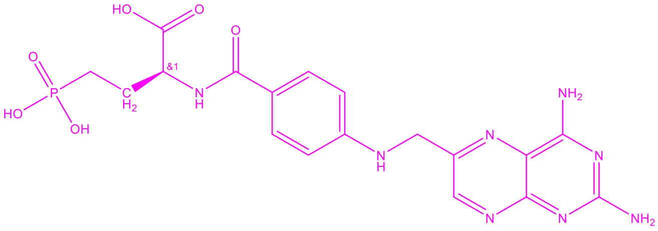	NC1=NC(N)=C2N=C(C=NC2=N1)CNC3=CC=C(C=C3)C(N[C@H](C(O)=O)CCP(O)(O)=O)=O	ZINC000028764057
103	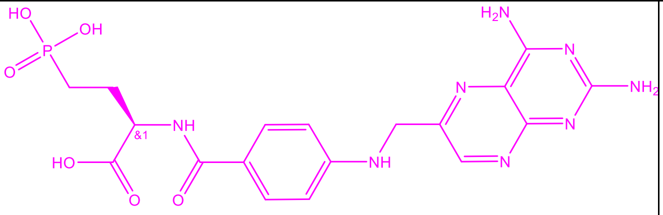	NC1=NC(N)=C2N=C(C=NC2=N1)CNC3=CC=C(C=C3)C(N[C@@H](C(O)=O)CCP(O)(O)=O)=O	ZINC000028764059
104	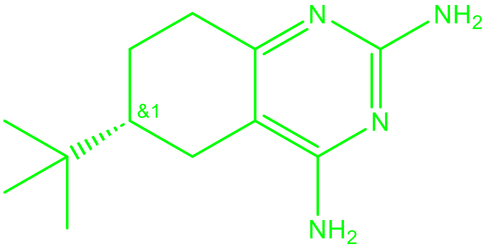	CC(C)([C@@H]1CCC2=NC(N)=NC(N)=C2C1)C	ZINC000003814902

105	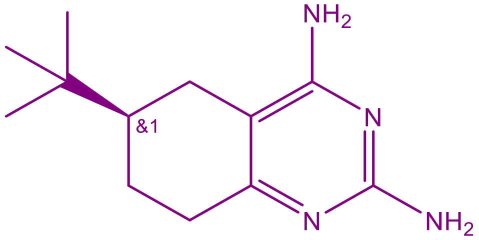	CC(C)([C@@H]1CCC2=NC(N)=NC(N)=C2C1)C	ZINC000005881944
106	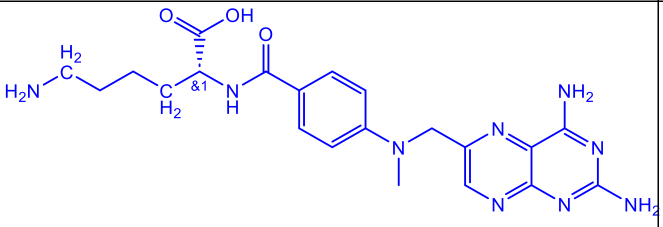	CN(C1=CC=C(C=C1)C(N[C@@H](C(O)=O)CCCCN)=O)CC2=CN=C3N=C(N=C(C3=N2)N)N	ZINC000008627939
107	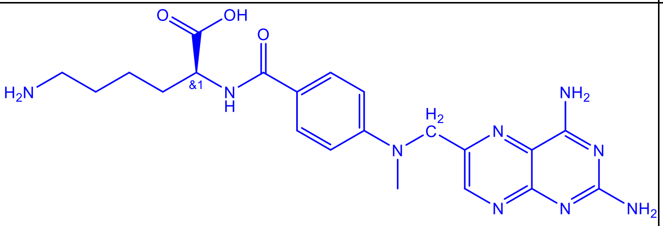	CN(C1=CC=C(C=C1)C(N[C@H](C(O)=O)CCCCN)=O)CC2=CN=C3N=C(N=C(C3=N2)N)N	ZINC000013284373
108	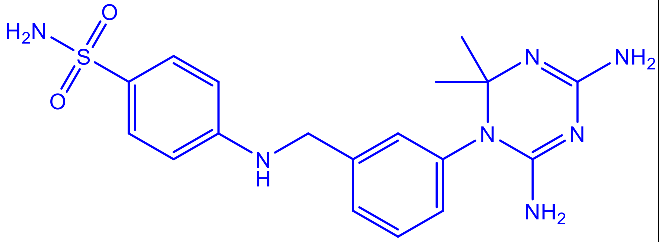	CC1(N=C(N=C(N1C2=CC=CC(CNC3=CC=C(C=C3)S(N)(=O)=O)=C2)N)N)C	ZINC000001587055
109	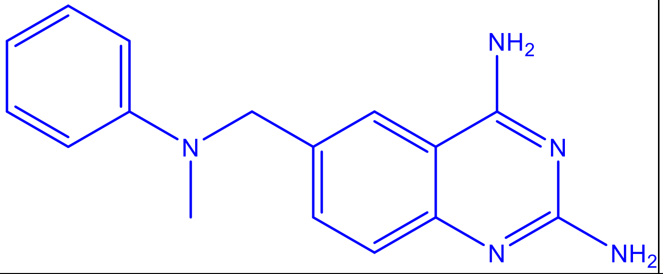	CN(C1=CC=CC=C1)CC2=CC=C3N=C(N=C(C3=C2)N)N	ZINC000003814840
110	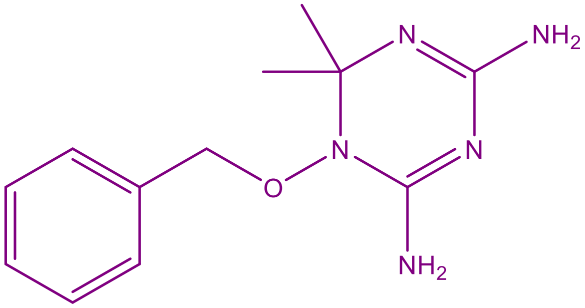	CC1(N=C(N=C(N1OCC2=CC=CC=C2)N)N)C	ZINC000072105910

111	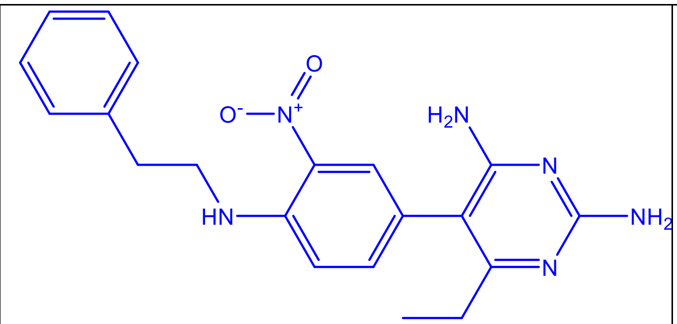	CCC1=NC(N)=NC(N)=C1C2=CC=C(C([N+]([O-])=O)=C2)NCCC3=CC=CC=C3	ZINC000005479500
112	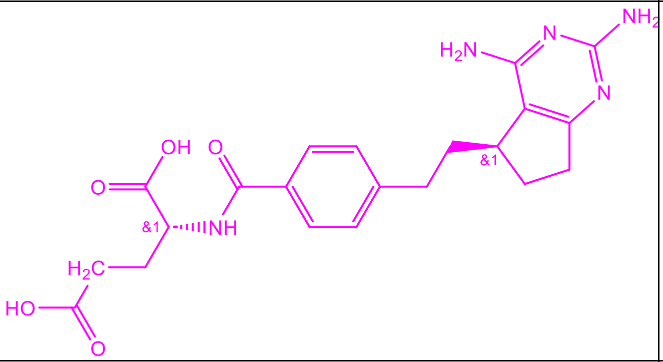	NC1=NC2=C([C@@H](CC2)CCC3=CC=C(C=C3)C(N[C@@H](C(O)=O)CCC(O)=O)=O)C(N)=N1	ZINC000001535479
113	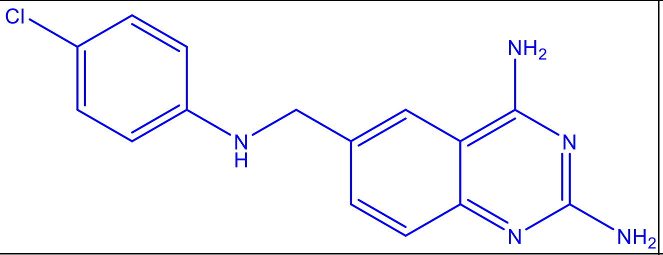	NC1=NC(N)=C2C=C(C=CC2=N1)CNC3=CC=C(C=C3)Cl	ZINC000001768091
114	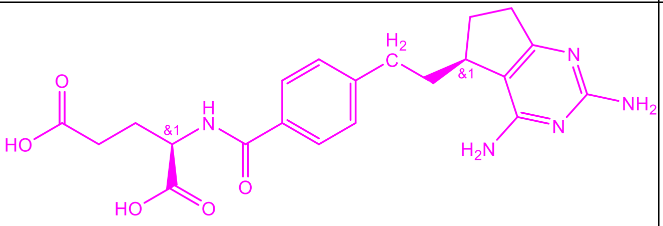	NC1=NC2=C([C@H](CC2)CCC3=CC=C(C=C3)C(N[C@@H](C(O)=O)CCC(O)=O)=O)C(N)=N1	ZINC000003789073
115	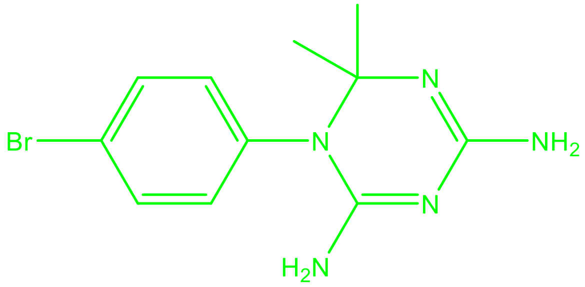	CC1(N=C(N=C(N1C2=CC=C(C=C2)Br)N)N)C	ZINC000000146421
116	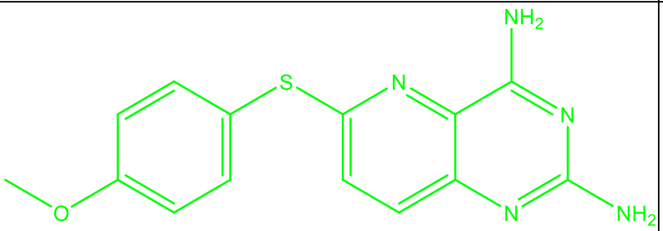	COC1=CC=C(C=C1)SC2=CC=C3N=C(N=C(C3=N2)N)N	ZINC000003814942

117	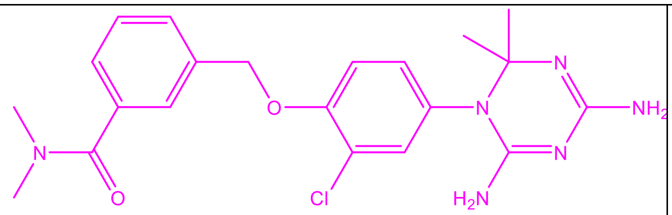	CN(C(C1=CC=CC(COC2=CC=C(C=C2Cl)N3C(N)=NC(N)=NC(C)3C)=C1)=O)C	ZINC000000538490
118	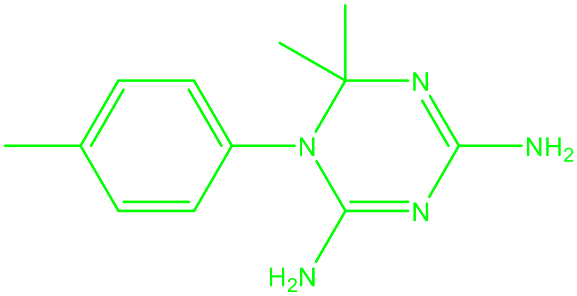	CC1=CC=C(C=C1)N2C(N)=NC(N)=NC(C)2C	ZINC000017040529
119	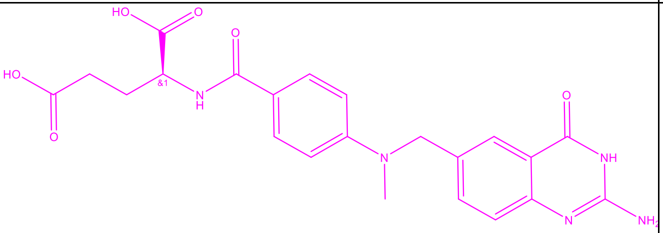	CN(C1=CC=C(C=C1)C(N[C@H](C(O)=O)CCC(O)=O)=O)CC2=CC=C3N=C(NC(C3=C2)=O)N	ZINC000017005705
120	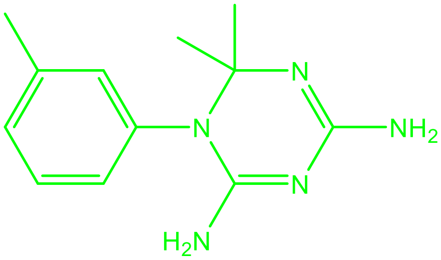	CC1=CC=CC(N2C(N)=NC(N)=NC(C)2C)=C1	ZINC000017025533
121	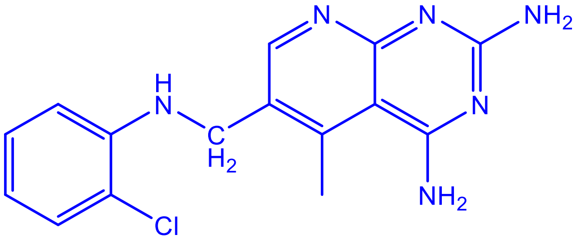	CC1=C(C=NC2=NC(N)=NC(N)=C12)CNC3=CC=CC=C3Cl	ZINC000000007637
122	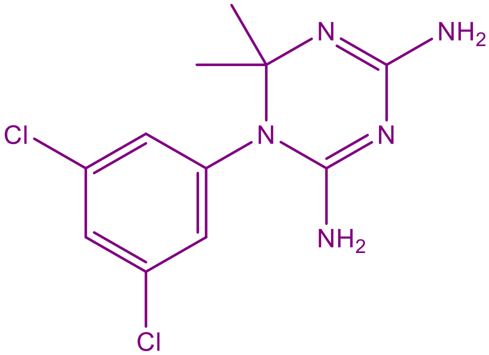	CC1(N=C(N=C(N1C2=CC(Cl)=CC(Cl)=C2)N)N)C	ZINC000029217677

123	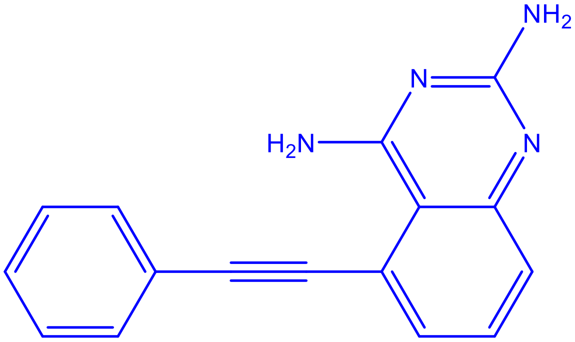	NC1=NC(N)=C2C(C#CC3=CC=CC=C3)=CC=CC2=N1	ZINC000013740533
124	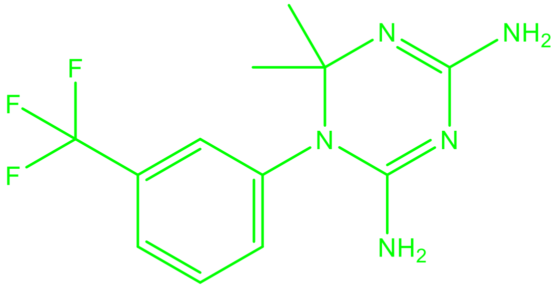	CC1(N=C(N=C(N1C2=CC=CC(C(F)(F)F)=C2)N)N)C	ZINC000000098181
125	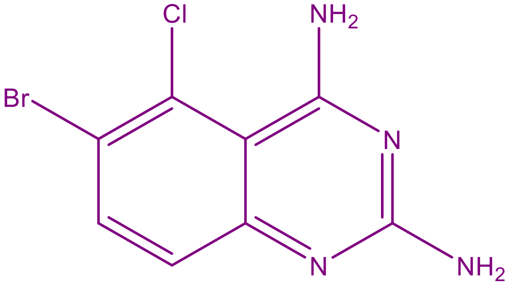	NC1=NC(N)=C2C(Cl)=C(C=CC2=N1)Br	ZINC000005973968
126	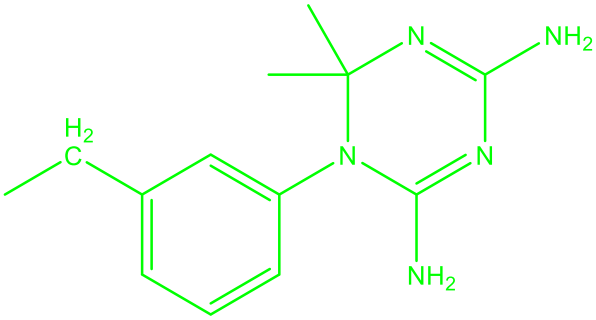	CCC1=CC=CC(N2C(N)=NC(N)=NC(C)2C)=C1	ZINC000013740716
127	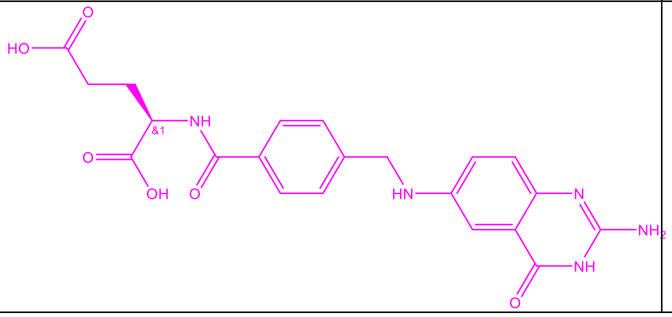	NC1=NC2=CC=C(C=C2C(N1)=O)NCC3=CC=C(C=C3)C(N[C@@H](C(O)=O)CCC(O)=O)=O	ZINC000001995991

128	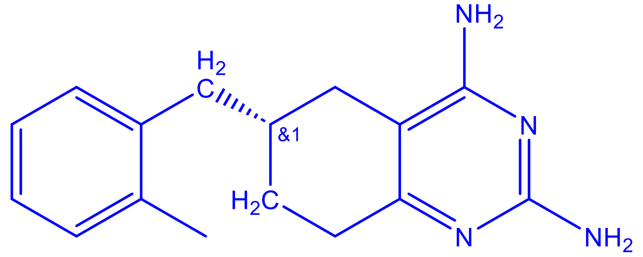	CC1=CC=CC=C1C[C@H]2CCC3=NC(N)=NC(N)=C3C2	ZINC000003814892
129	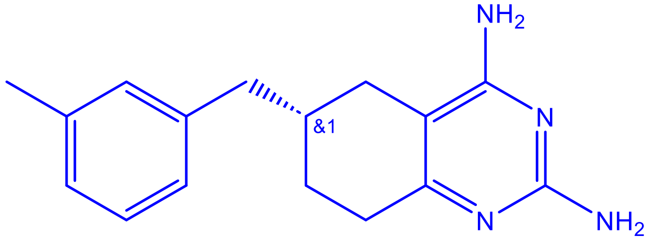	CC1=CC=CC(C[C@H]2CCC3=NC(N)=NC(N)=C3C2)=C1	ZINC000003814893
130	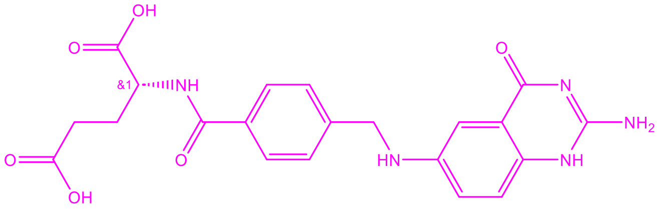	NC1=NC(C2=CC(NCC3=CC=C(C=C3)C(N[C@@H](C(O)=O)CCC(O)=O)=O)=CC=C2N1)=O	ZINC000005385736
131	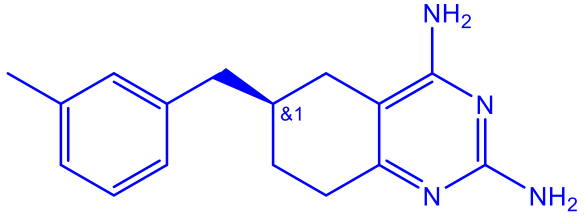	CC1=CC=CC(C[C@@H]2CCC3=NC(N)=NC(N)=C3C2)=C1	ZINC000005892624
132	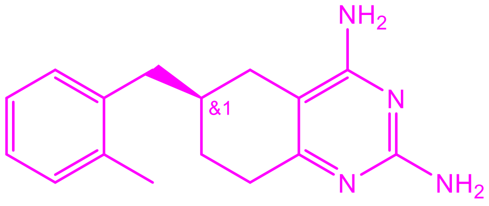	CC1=CC=CC=C1C[C@@H]2CCC3=NC(N)=NC(N)=C3C2	ZINC000005892766
133	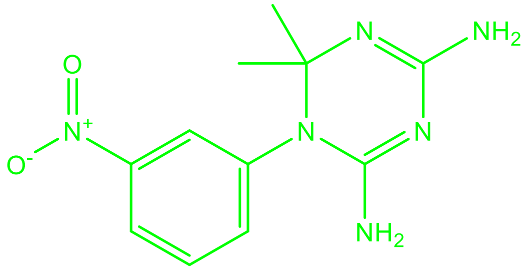	CC1(N=C(N=C(N1C2=CC=CC([N+]([O-])=O)=C2)N)N)C	ZINC000017005686

134	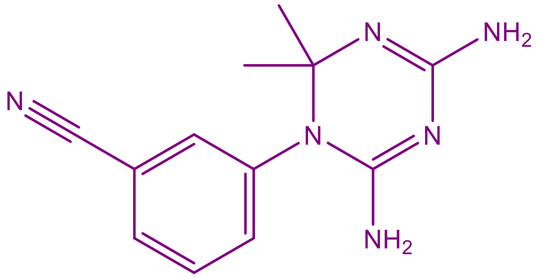	CC1(N=C(N=C(N1C2=CC=CC(C#N)=C2)N)N)C	ZINC000017428337
135	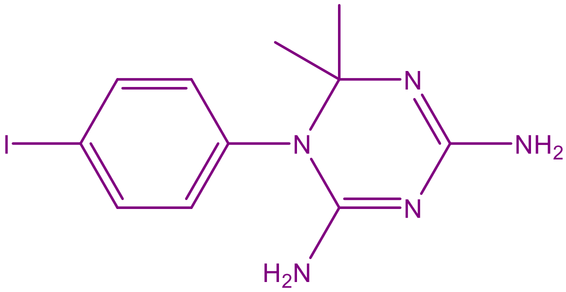	CC1(N=C(N=C(N1C2=CC=C(C=C2)I)N)N)C	ZINC000003074278
136	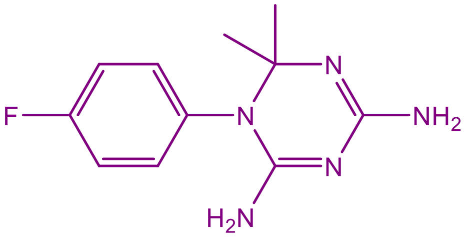	CC1(N=C(N=C(N1C2=CC=C(C=C2)F)N)N)C	ZINC000017126763
137	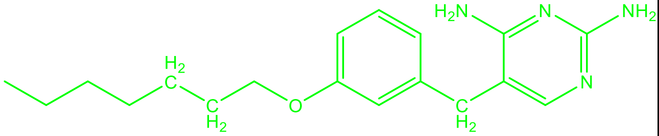	CCCCCCCOC1=CC=CC(CC2=CN=C(N=C2N)N)=C1	ZINC000034801949
138	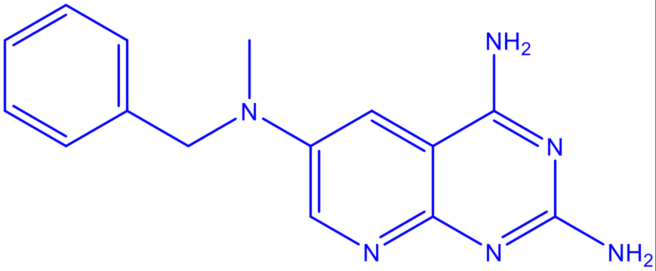	CN(C1=CN=C2N=C(N=C(C2=C1)N)N)CC3=CC=CC=C3	ZINC000003814959
139	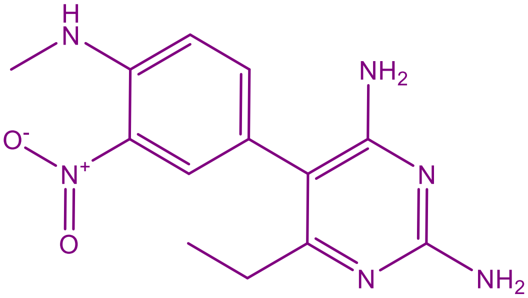	CCC1=NC(N)=NC(N)=C1C2=CC=C(C([N+]([O-])=O)=C2)NC	ZINC000005479493

140	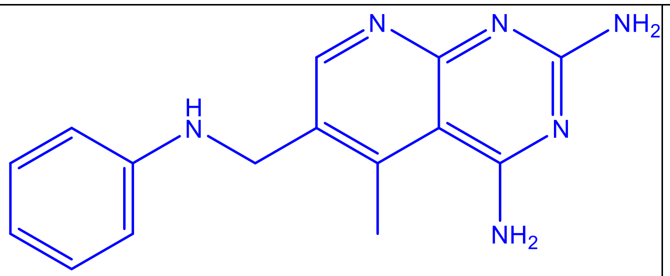	CC1=C(C=NC2=NC(N)=NC(N)=C12)CNC3=CC=CC=C3	ZINC000000023781
141	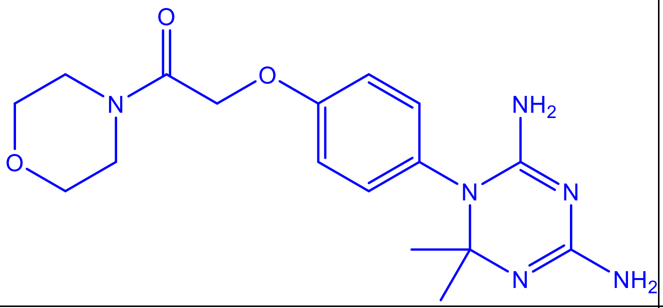	CC1(N=C(N=C(N1C2=CC=C(C=C2)OCC(N3CCOCC3)=O)N)N)C	ZINC000001724842
142	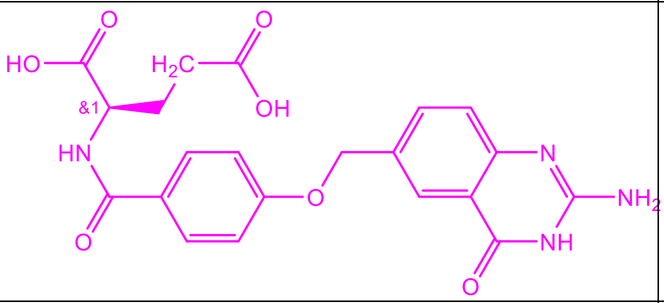	NC1=NC2=CC=C(C=C2C(N1)=O)COC3=CC=C(C=C3)C(N[C@@H](C(O)=O)CCC(O)=O)=O	ZINC000004621661
143	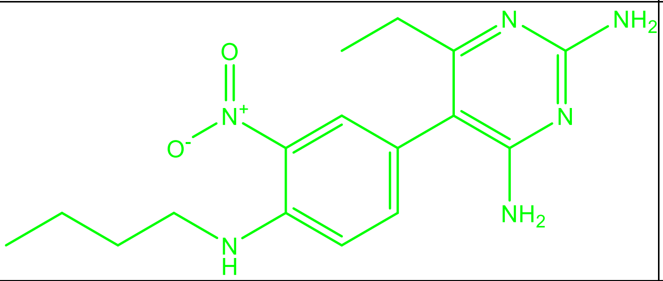	CCCCNC1=CC=C(C=C1[N+]([O-])=O)C2=C(N=C(N=C2CC)N)N	ZINC000005479323
144	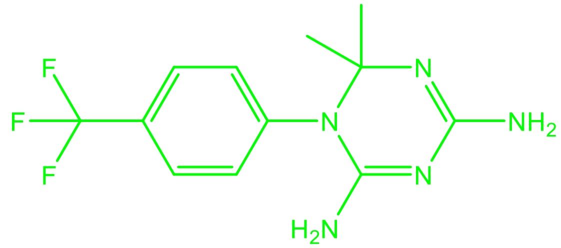	CC1(N=C(N=C(N1C2=CC=C(C=C2)C(F)(F)F)N)N)C	ZINC000017039530
145	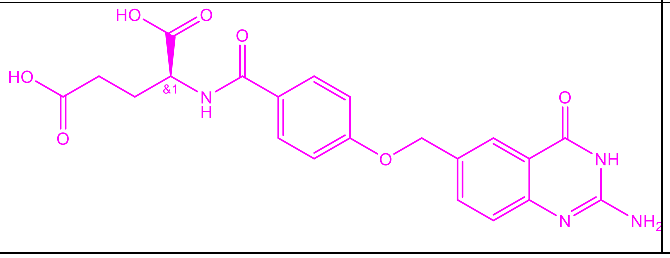	NC1=NC2=CC=C(C=C2C(N1)=O)COC3=CC=C(C=C3)C(N[C@H](C(O)=O)CCC(O)=O)=O	ZINC000017106187

146	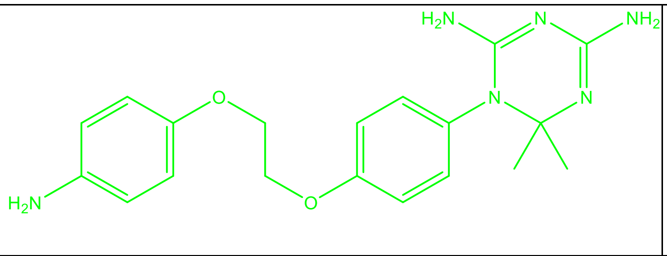	CC1(N=C(N=C(N1C2=CC=C(C=C2)OCCOC3=CC=C(C=C3)N)N)N)C	ZINC000001710094
147	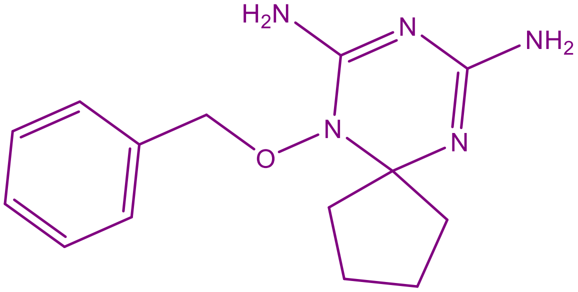	NC1=NC2(N(C(N)=N1)OCC3=CC=CC=C3)CCCC2	ZINC000072182239
148	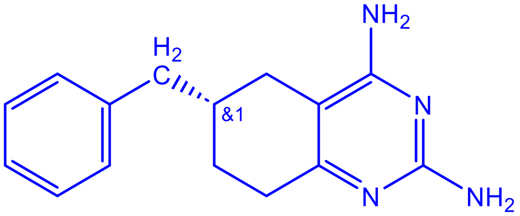	NC1=NC(N)=C2C(CC[C@@H](C2)CC3=CC=CC=C3)=N1	ZINC000003814891
149	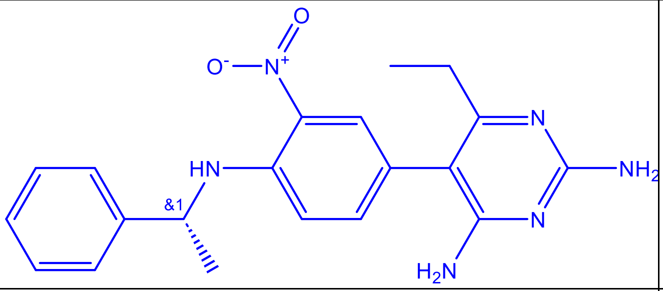	CCC1=NC(N)=NC(N)=C1C2=CC=C(C([N+]([O-])=O)=C2)N[C@@H](C3=CC=CC=C3)C	ZINC000005479497
150	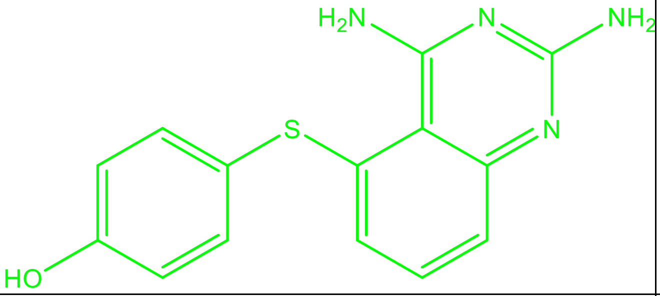	NC1=NC(N)=C2C(C=CC=C2SC3=CC=C(C=C3)O)=N1	ZINC000005974273
151	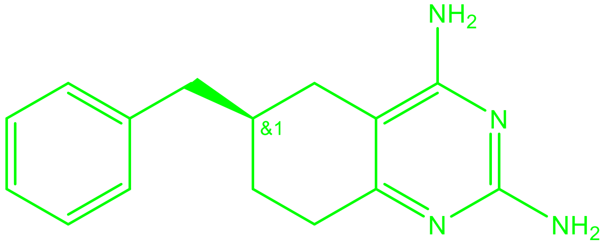	NC1=NC(N)=C2C(CC[C@H](C2)CC3=CC=CC=C3)=N1	ZINC000005974593

152	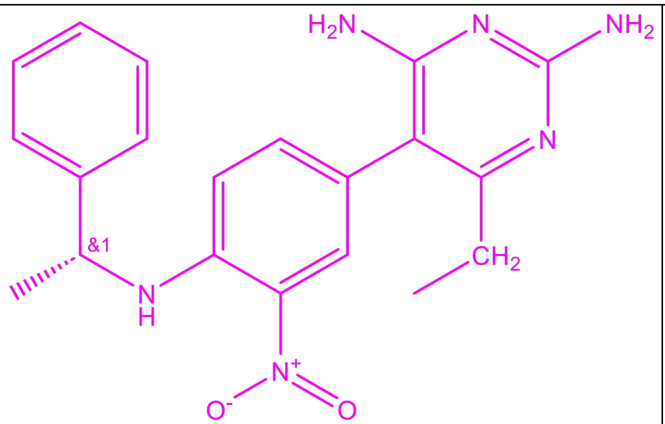	CCC1=NC(N)=NC(N)=C1C2=CC=C(C([N+]([O-])=O)=C2)N[C@@H](C3=CC=CC=C3)C	ZINC000006294619
153	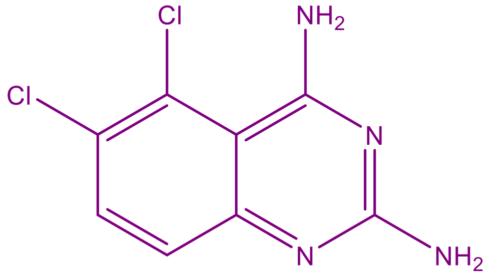	NC1=NC(N)=C2C(Cl)=C(C=CC2=N1)Cl	ZINC000005973969
154	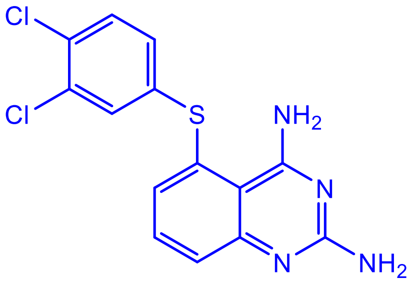	NC1=NC(N)=C2C(C=CC=C2SC3=CC=C(C(Cl)=C3)Cl)=N1	ZINC000025968915
155	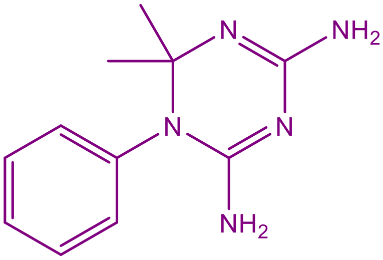	CC1(N=C(N=C(N1C2=CC=CC=C2)N)N)C	ZINC000016981849
156	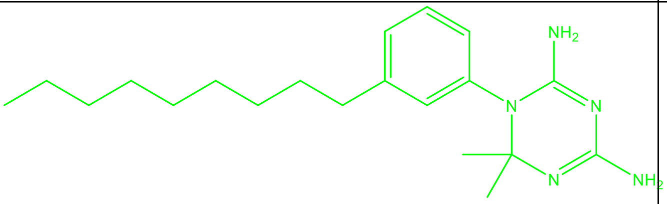	CCCCCCCCCC1=CC=CC(N2C(N)=NC(N)=NC(C)2C)=C1	ZINC000001609235

157	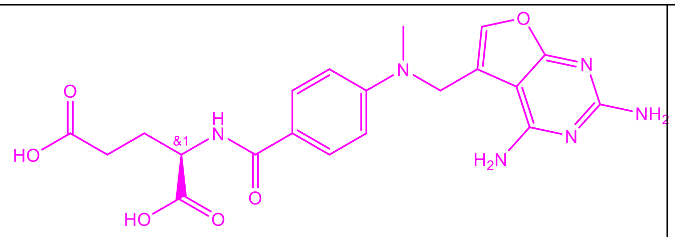	CN(C1=CC=C(C=C1)C(N[C@@H](C(O)=O)CCC(O)=O)=O)CC2=COC3=NC(N)=NC(N)=C23	ZINC000002046975
158	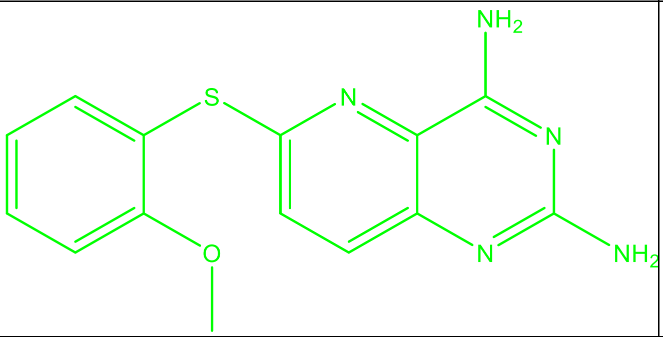	COC1=CC=CC=C1SC2=CC=C3N=C(N=C(C3=N2)N)N	ZINC000003814941
159	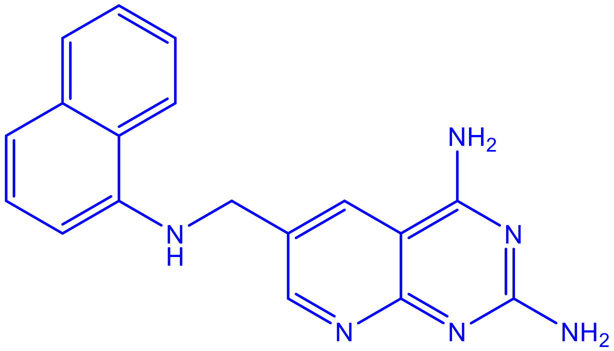	NC1=NC(N)=C2C=C(C=NC2=N1)CNC3=CC=CC4=CC=CC=C34	ZINC000003815019
160	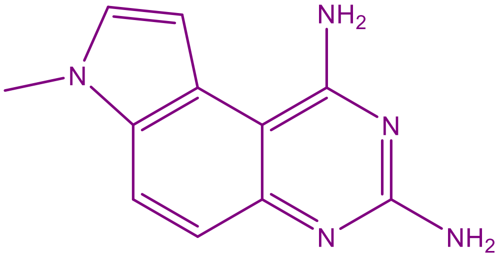	CN1C=CC2=C1C=CC3=NC(N)=NC(N)=C32	ZINC000005893315
161	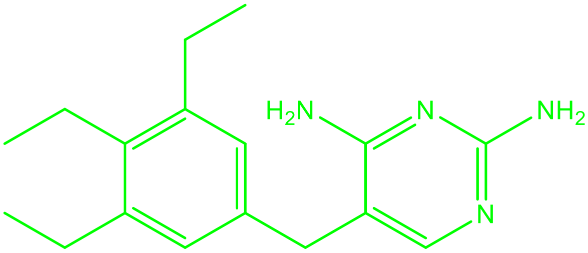	CCC1=CC(CC2=CN=C(N=C2N)N)=CC(CC)=C1CC	ZINC000013726792

162	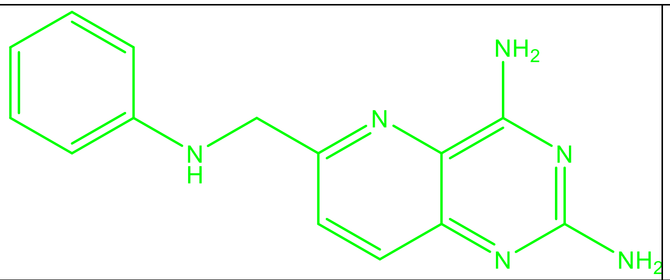	NC1=NC(N)=C2N=C(C=CC2=N1)CNC3=CC=CC=C3	ZINC000003814922
163	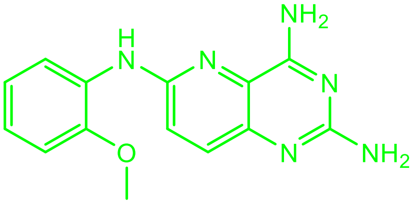	COC1=CC=CC=C1NC2=CC=C3N=C(N=C(C3=N2)N)N	ZINC000003814934
164	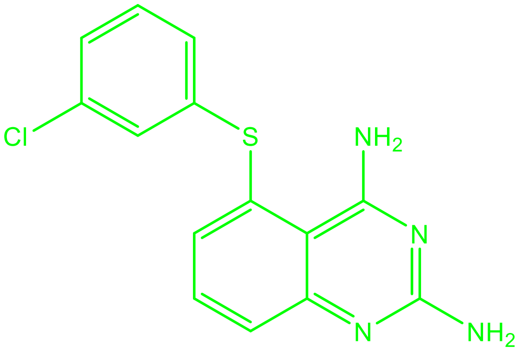	NC1=NC(N)=C2C(C=CC=C2SC3=CC=CC(Cl)=C3)=N1	ZINC000005974038
165	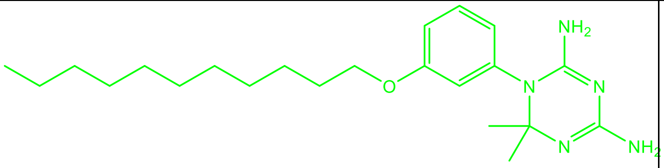	CCCCCCCCCCCOC1=CC=CC(N2C(N)=NC(N)=NC(C)2C)=C1	ZINC000001609236
166	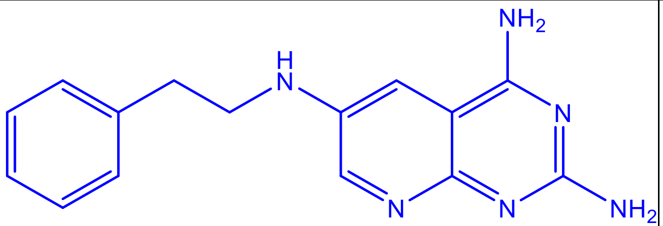	NC1=NC(N)=C2C=C(C=NC2=N1)NCCC3=CC=CC=C3	ZINC000005974513
167	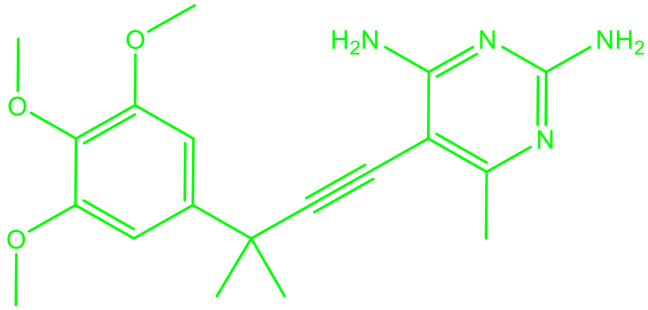	COC1=CC(C(C)(C#CC2=C(N=C(N=C2N)N)C)C)=CC(OC)=C1OC	ZINC000053683841

168	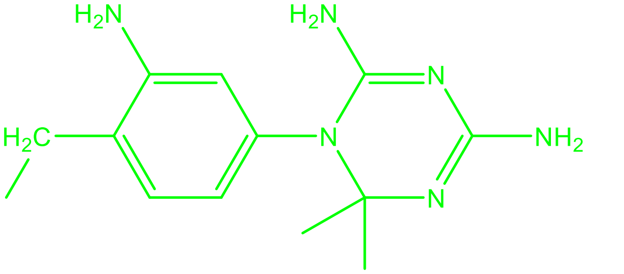	CCC1=CC=C(C=C1N)N2C(N)=NC(N)=NC(C)2C	ZINC000029219414
169	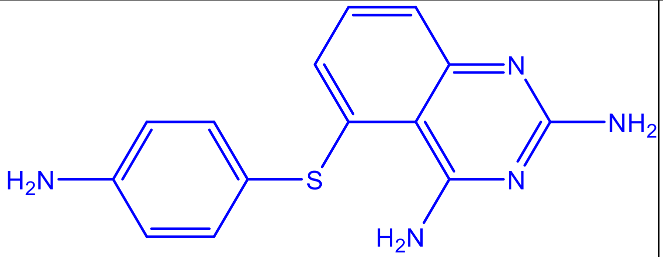	NC1=CC=C(C=C1)SC2=CC=CC3=NC(N)=NC(N)=C23	ZINC000005933621
170	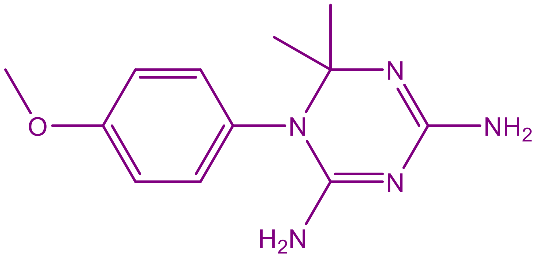	COC1=CC=C(C=C1)N2C(N)=NC(N)=NC(C)2C	ZINC000001842830
171	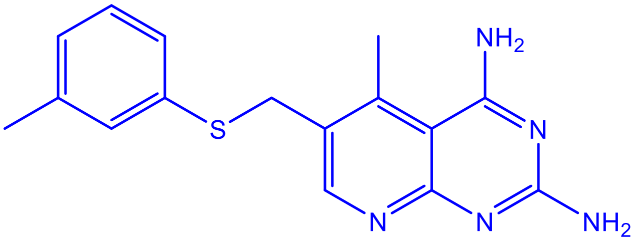	CC1=CC=CC(SCC2=CN=C3N=C(N=C(C3=C2C)N)N)=C1	ZINC000003815000
172	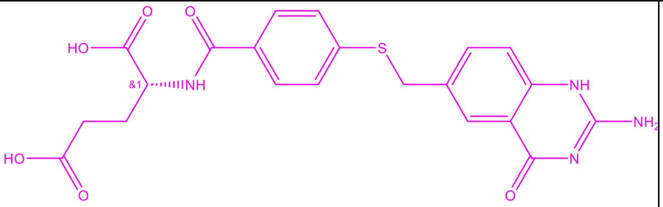	NC1=NC(C2=CC(CSC3=CC=C(C=C3)C(N[C@@H](C(O)=O)CCC(O)=O)=O)=CC=C2N1)=O	ZINC000009633571
173	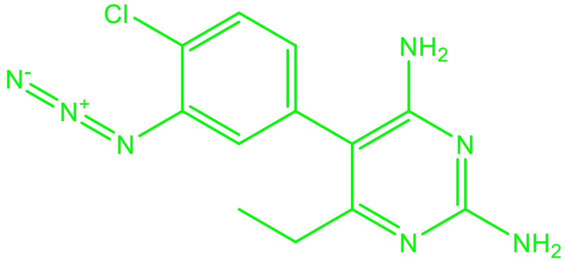	CCC1=NC(N)=NC(N)=C1C2=CC=C(C(N=[N+]=[N-])=C2)Cl	ZINC000013762607

174	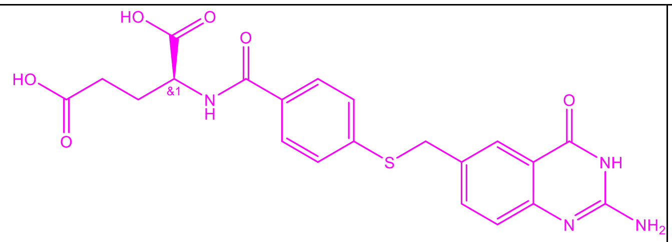	NC1=NC2=CC=C(C=C2C(N1)=O)CSC3=CC=C(C=C3)C(N[C@H](C(O)=O)CCC(O)=O)=O	ZINC000017106166
175	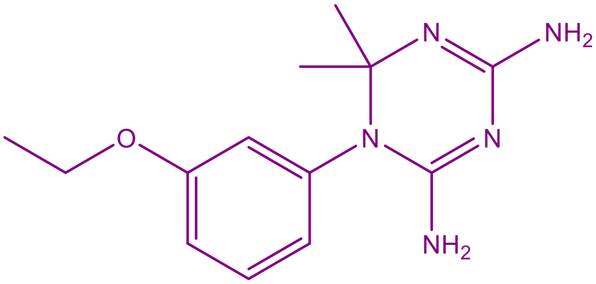	CCOC1=CC=CC(N2C(N)=NC(N)=NC(C)2C)=C1	ZINC000013740720
176	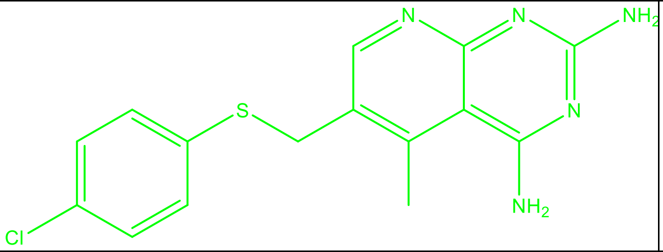	CC1=C(C=NC2=NC(N)=NC(N)=C12)CSC3=CC=C(C=C3)Cl	ZINC000003815006
177	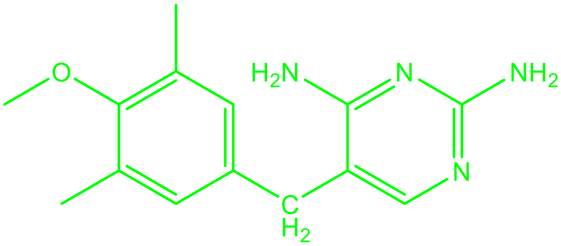	COC1=C(C=C(C=C1C)CC2=CN=C(N=C2N)N)C	ZINC000028263538
178	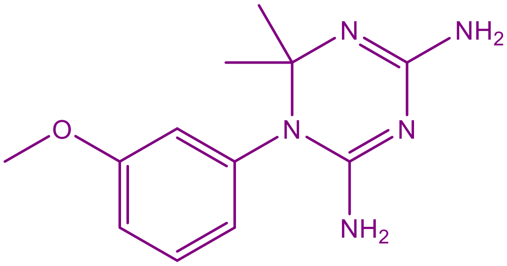	COC1=CC=CC(N2C(N)=NC(N)=NC(C)2C)=C1	ZINC000017039688
179	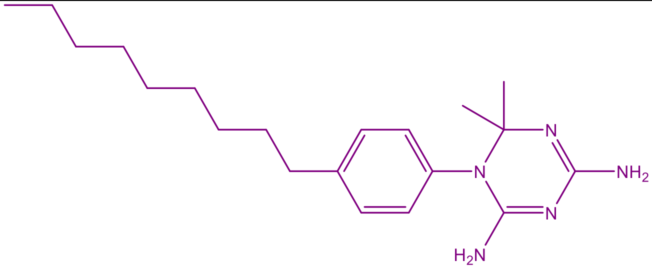	CCCCCCCCCC1=CC=C(C=C1)N2C(N)=NC(N)=NC(C)2C	ZINC000043706317

180	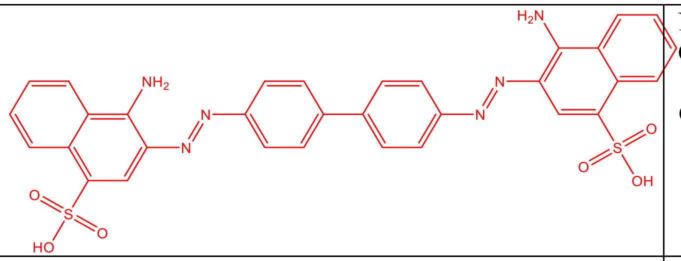	NC1=C(C=C(C2=CC=CC=C12)S(=O)(O)=O)/N=N/C3=CC=C(C=C3)C4=CC=C(C=C4)/N=N/C5=CC(S(=O)(O)=O)=C6C=CC=CC6=C5N	ZINC000003830554
181	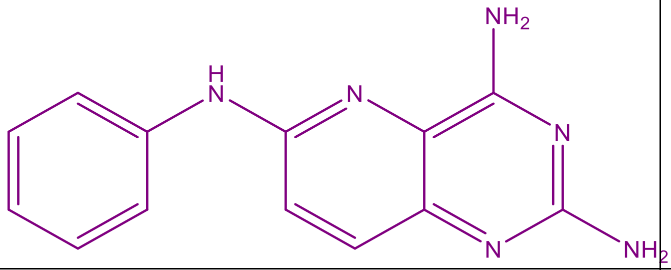	NC1=NC(N)=C2N=C(C=CC2=N1)NC3=CC=CC=C3	ZINC000003814933
182	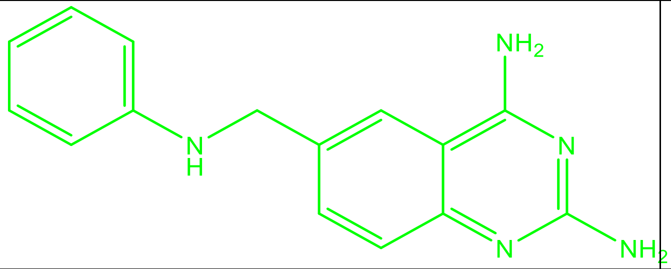	NC1=NC(N)=C2C=C(C=CC2=N1)CNC3=CC=CC=C3	ZINC000003814839
183	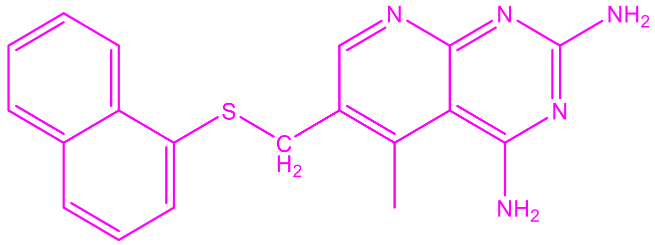	CC1=C(C=NC2=NC(N)=NC(N)=C12)CSC3=CC=CC4=CC=CC=C34	ZINC000005891537
184	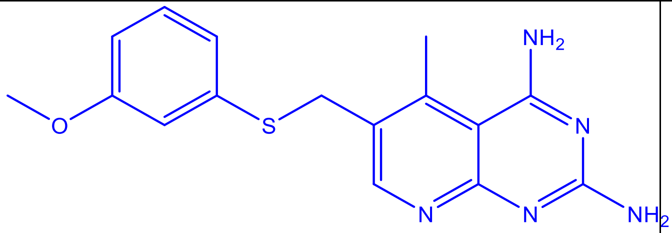	COC1=CC=CC(SCC2=CN=C3N=C(N=C(C3=C2C)N)N)=C1	ZINC000003815002
185	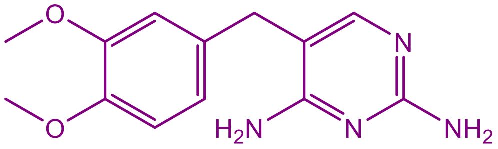	COC1=CC=C(C=C1OC)CC2=CN=C(N=C2N)N	ZINC000000024946

186	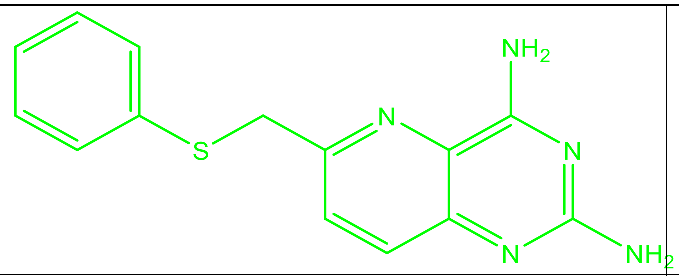	NC1=NC(N)=C2N=C(C=CC2=N1)CSC3=CC=CC=C3	ZINC000003814918
187	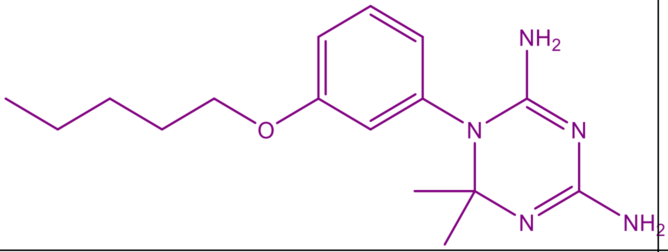	CCCCCOC1=CC=CC(N2C(N)=NC(N)=NC(C)2C)=C1	ZINC000029219121
188	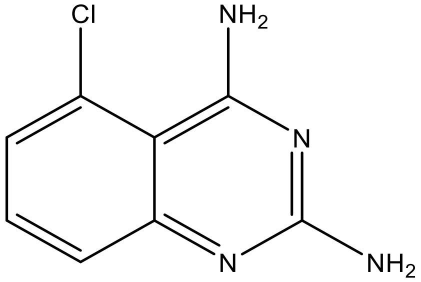	NC1=NC(N)=C2C(Cl)=CC=CC2=N1	ZINC000000344343
189	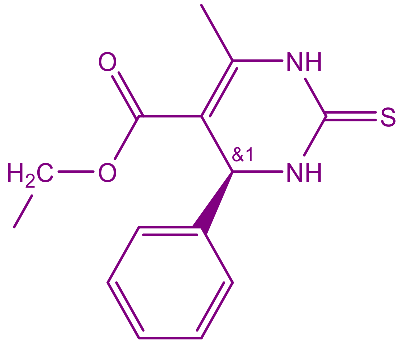	CCOC(C1=C(NC(N[C@H]1C2=CC=CC=C2)=S)C)=O	ZINC000018038509
190	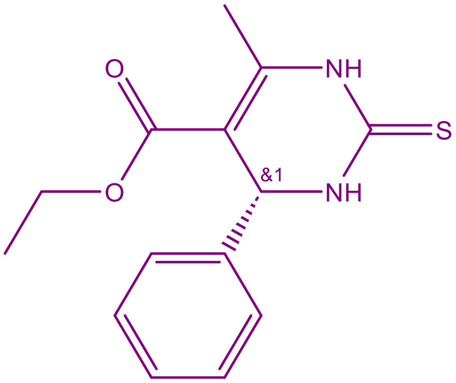	CCOC(C1=C(NC(N[C@@H]1C2=CC=CC=C2)=S)C)=O	ZINC000018068419

191	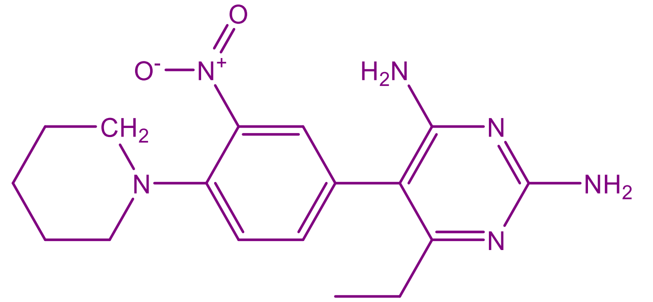	CCC1=NC(N)=NC(N)=C1C2=CC=C(C([N+]([O-])=O)=C2)N3CCCCC3	ZINC000005386671
192	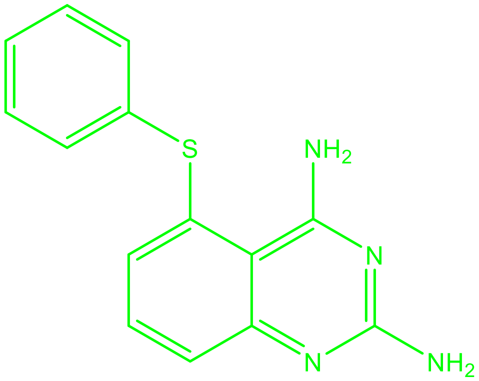	NC1=NC(N)=C2C(C=CC=C2SC3=CC=CC=C3)=N1	ZINC000005974663
193	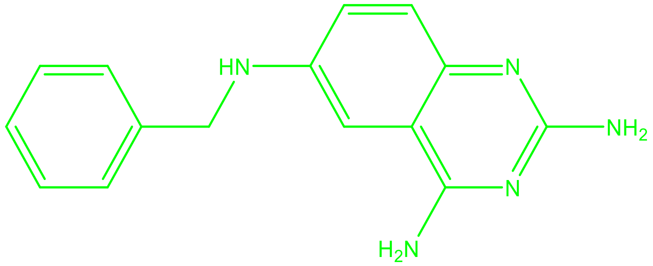	NC1=NC(N)=C2C=C(C=CC2=N1)NCC3=CC=CC=C3	ZINC000005974540
194	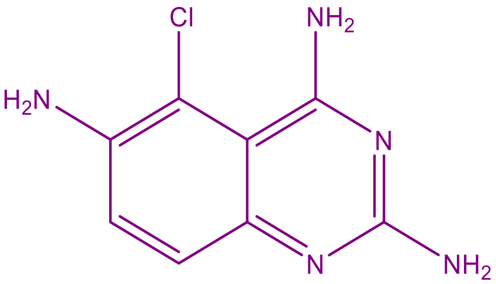	NC1=NC(N)=C2C(Cl)=C(C=CC2=N1)N	ZINC000013282329
195	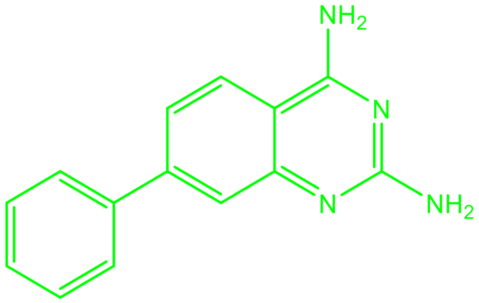	NC1=NC(N)=C2C=CC(C3=CC=CC=C3)=CC2=N1	ZINC000040186681

196	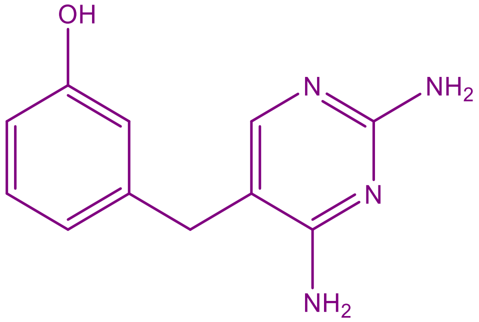	NC1=NC=C(C(N)=N1)CC2=CC=CC(O)=C2	ZINC000013726702
197	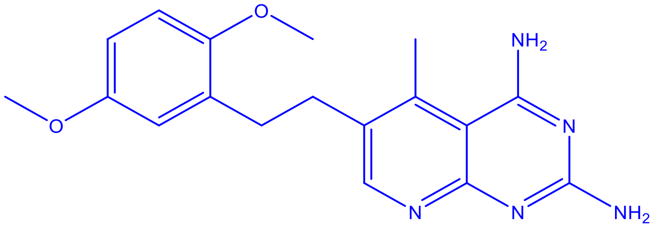	COC1=CC=C(C(CCC2=CN=C3N=C(N=C(C3=C2C)N)N)=C1)OC	ZINC000003815012
198	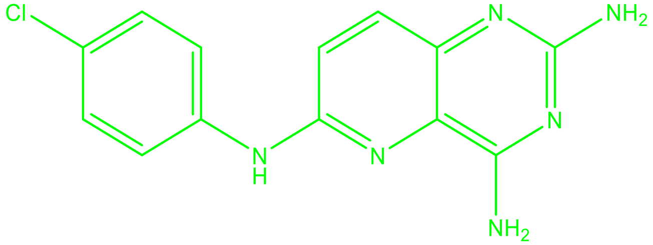	NC1=NC(N)=C2N=C(C=CC2=N1)NC3=CC=C(C=C3)Cl	ZINC000003814935
199	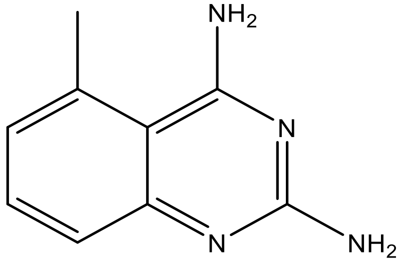	CC1=CC=CC2=NC(N)=NC(N)=C12	ZINC000012950643
200	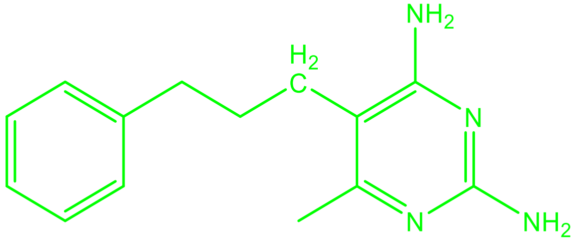	CC1=NC(N)=NC(N)=C1CCCC2=CC=CC=C2	ZINC000013283435
201	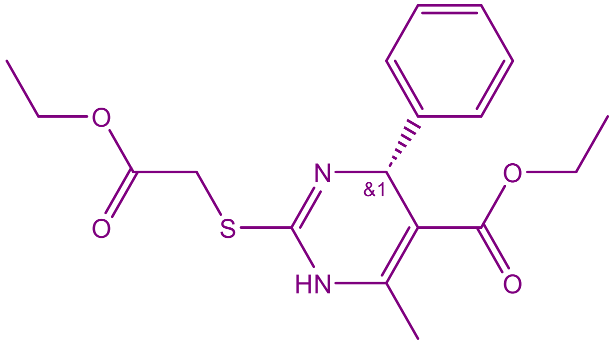	CCOC(CSC1=N[C@@H](C(C(OCC)=O)=C(N1)C)C2=CC=CC=C2)=O	ZINC000033769556

202	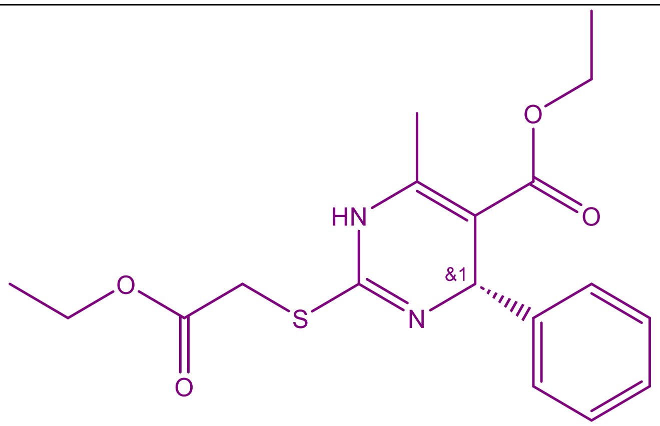	CCOC(CSC1=N[C@H](C(C(OCC)=O)=C(N1)C)C2=CC=CC=C2)=O	ZINC000033769557
203	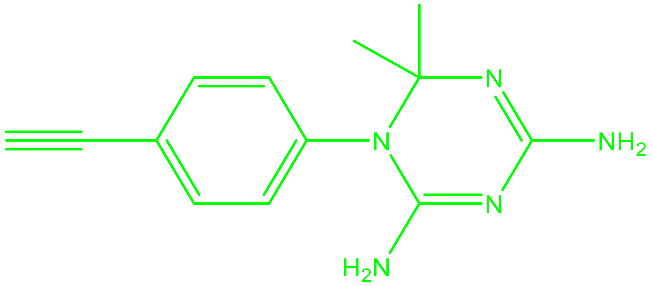	C#CC1=CC=C(C=C1)N2C(N)=NC(N)=NC(C)2C	ZINC000029219695
204	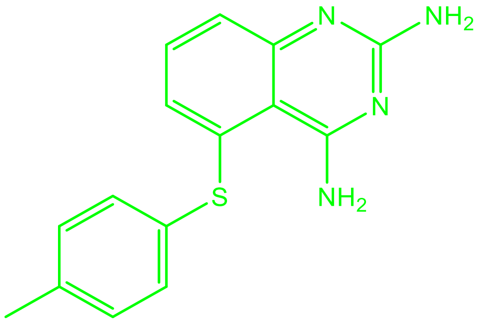	CC1=CC=C(C=C1)SC2=CC=CC3=NC(N)=NC(N)=C23	ZINC000000172633
205	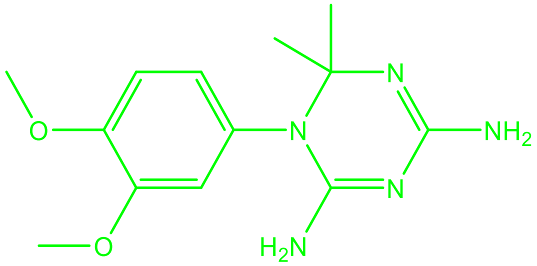	COC1=CC=C(C=C1OC)N2C(N)=NC(N)=NC(C)2C	ZINC000005261011
206	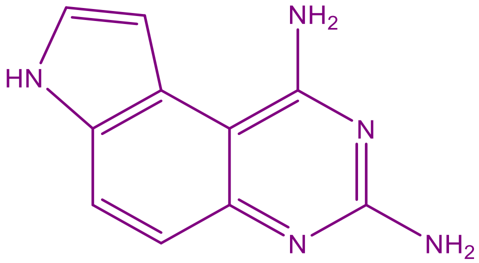	NC1=NC(N)=C2C3=C(C=CC2=N1)NC=C3	ZINC000001578473

207	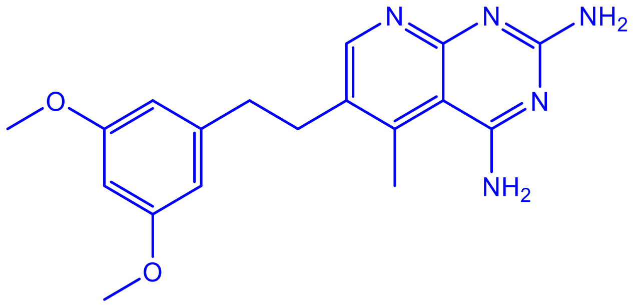	COC1=CC(CCC2=CN=C3N=C(N=C(C3=C2C)N)N)=CC(OC)=C1	ZINC000005891405
208	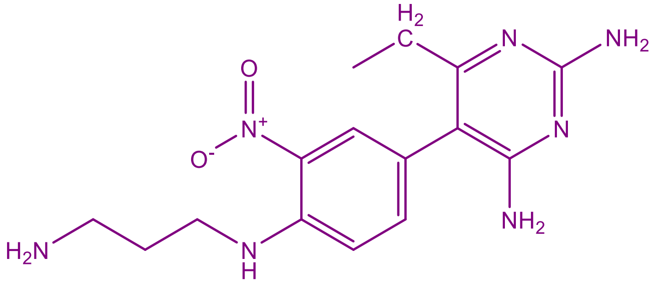	CCC1=NC(N)=NC(N)=C1C2=CC=C(C([N+]([O-])=O)=C2)NCCCN	ZINC000026501191
209	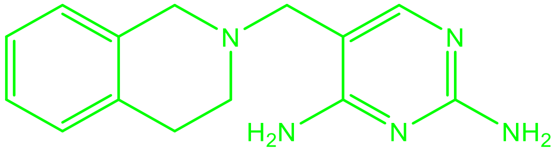	NC1=NC=C(C(N)=N1)CN2CCC3=CC=CC=C3C2	ZINC000013520504
210	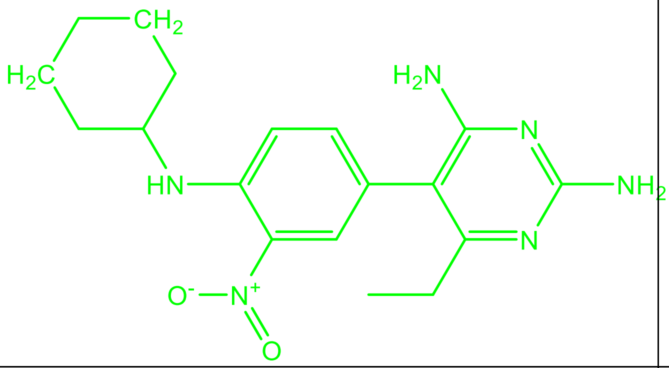	CCC1=NC(N)=NC(N)=C1C2=CC=C(C([N+]([O-])=O)=C2)NC3CCCCC3	ZINC000005479328
211	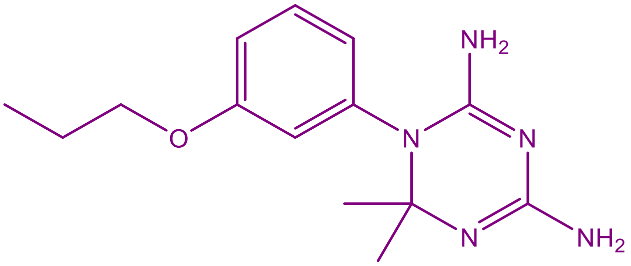	CCCOC1=CC=CC(N2C(N)=NC(N)=NC(C)2C)=C1	ZINC000029218525

212	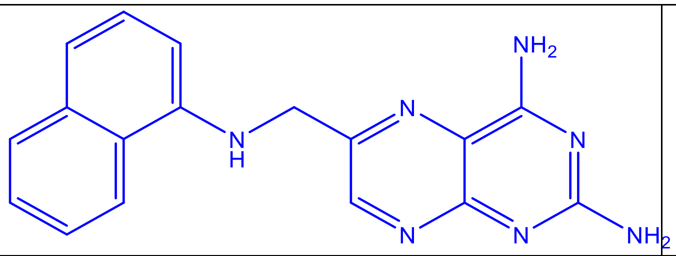	NC1=NC(N)=C2N=C(C=NC2=N1)CNC3=CC=CC4=CC=CC=C34	ZINC000000014672
213	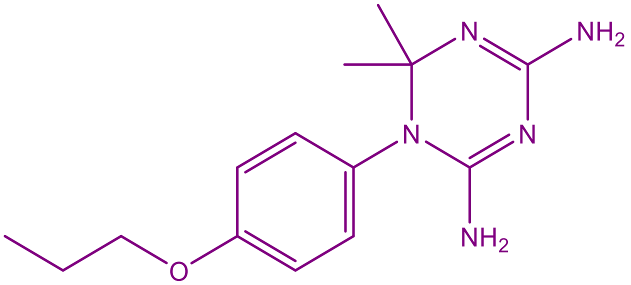	CCCOC1=CC=C(C=C1)N2C(N)=NC(N)=NC(C)2C	ZINC000017039700
214	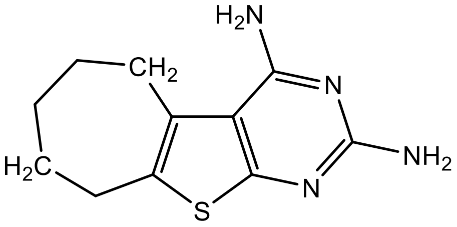	NC1=NC2=C(C(N)=N1)C(CCCCC3)=C3S2	ZINC000005847308
215	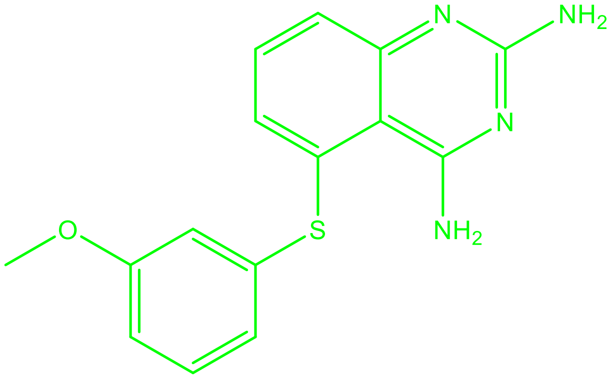	COC1=CC=CC(SC2=CC=CC3=NC(N)=NC(N)=C23)=C1	ZINC000005889719
216	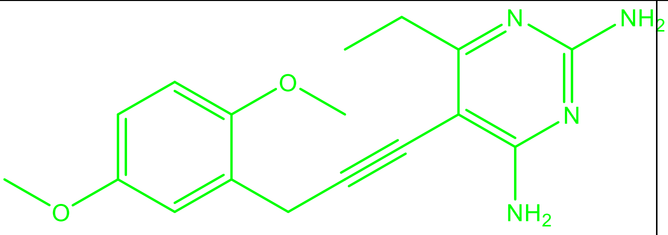	CCC1=NC(N)=NC(N)=C1C#CCC2=CC(OC)=CC=C2OC	ZINC000045484444
217	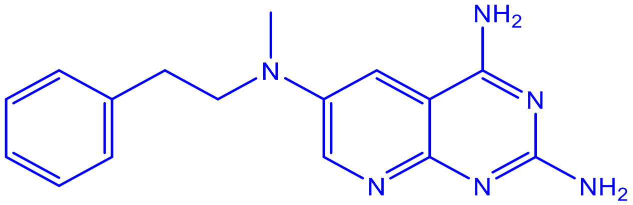	CN(C1=CN=C2N=C(N=C(C2=C1)N)N)CCC3=CC=CC=C3	ZINC000005887816

218	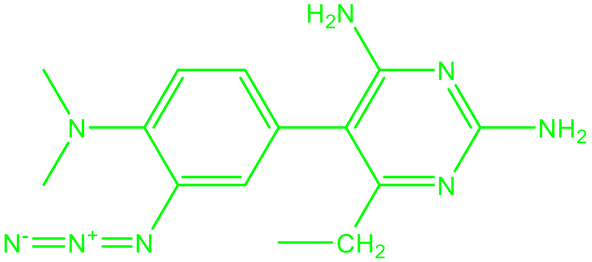	CCC1=NC(N)=NC(N)=C1C2=CC=C(C(N=[N+]=[N-])=C2)N(C)C	ZINC000016942585
219	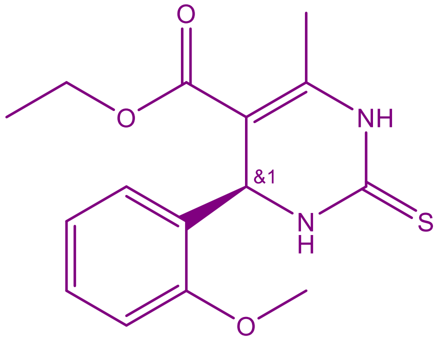	CCOC(C1=C(NC(N[C@H]1C2=CC=CC=C2OC)=S)C)=O	ZINC000000974809
220	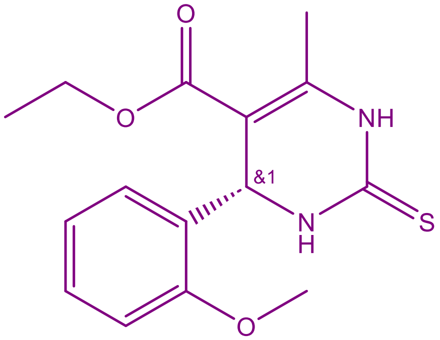	CCOC(C1=C(NC(N[C@@H]1C2=CC=CC=C2OC)=S)C)=O	ZINC000000974810
221	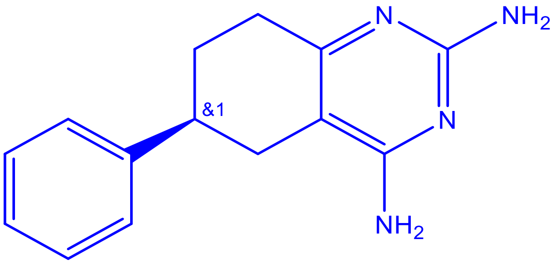	NC1=NC(N)=C2C(CC[C@@H](C2)C3=CC=CC=C3)=N1	ZINC000005974680
222	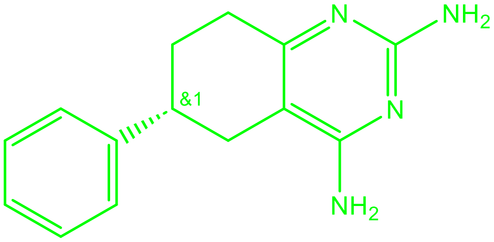	NC1=NC(N)=C2C(CC[C@H](C2)C3=CC=CC=C3)=N1	ZINC000005974703

223	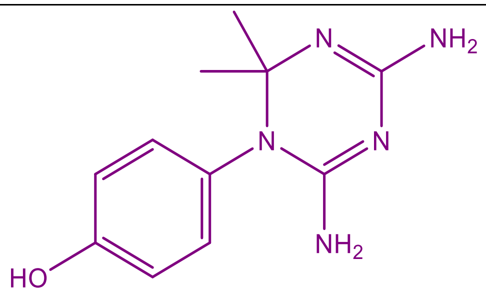	CC1(N=C(N=C(N1C2=CC=C(C=C2)O)N)N)C	ZINC000005835905
224	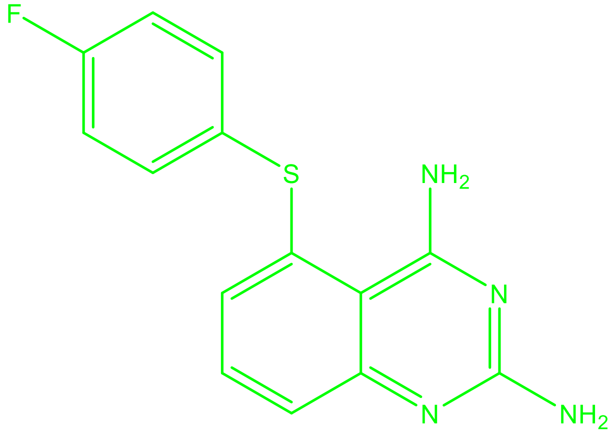	NC1=NC(N)=C2C(C=CC=C2SC3=CC=C(C=C3)F)=N1	ZINC000005974272
225	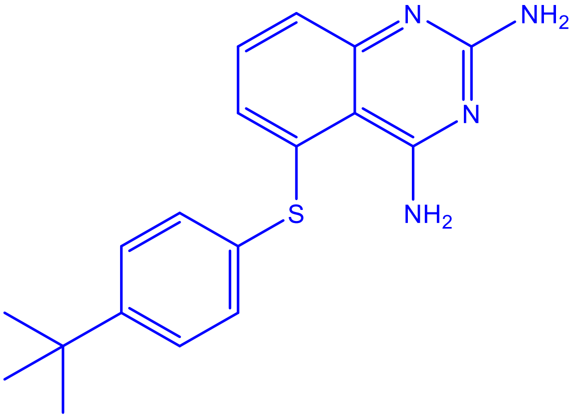	CC(C)(C1=CC=C(C=C1)SC2=CC=CC3=NC(N)=NC(N)=C23)C	ZINC000008657832
226	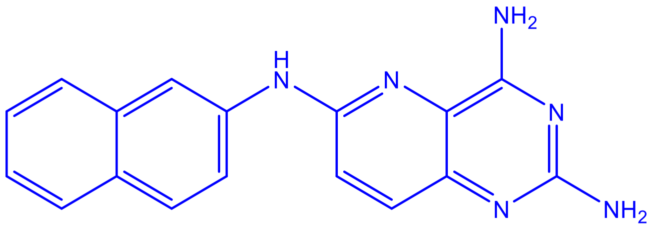	NC1=NC(N)=C2N=C(C=CC2=N1)NC3=CC=C4C=CC=CC4=C3	ZINC000013781975
227	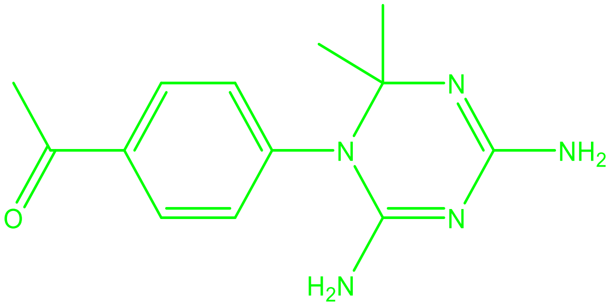	CC(C1=CC=C(C=C1)N2C(N)=NC(N)=NC(C)2C)=O	ZINC000025968981

228	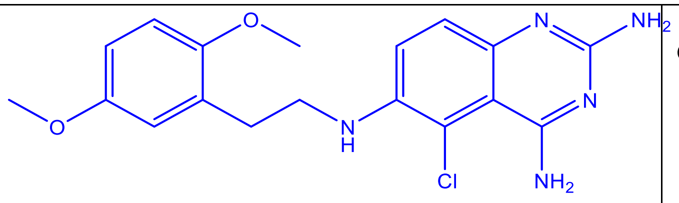	COC1=CC=C(C(CCNC2=CC=C3N=C(N=C(C3=C2Cl)N)N)=C1)OC	ZINC000005888996
229	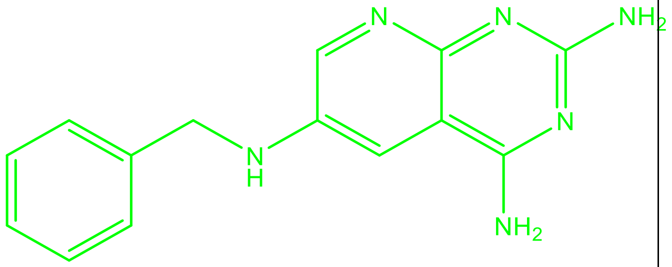	NC1=NC(N)=C2C=C(C=NC2=N1)NCC3=CC=CC=C3	ZINC000005974538
230	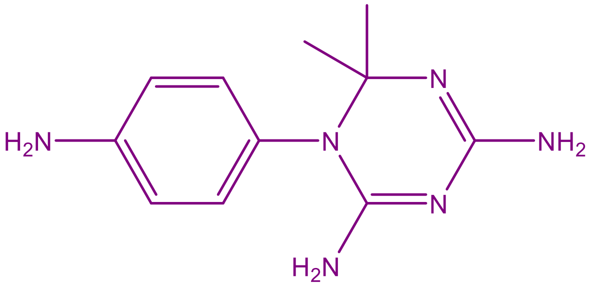	CC1(N=C(N=C(N1C2=CC=C(C=C2)N)N)N)C	ZINC000001716761
231	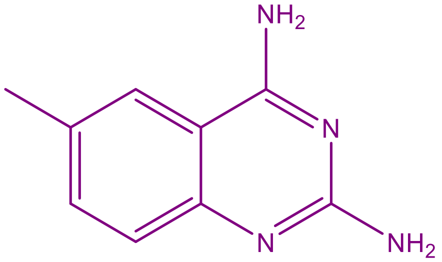	CC1=CC=C2N=C(N=C(C2=C1)N)N	ZINC000012343992
232	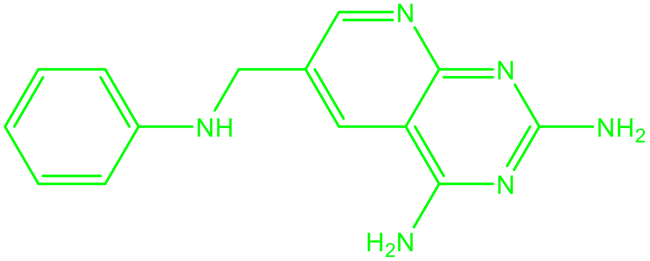	NC1=NC(N)=C2C=C(C=NC2=N1)CNC3=CC=CC=C3	ZINC000013529600
233	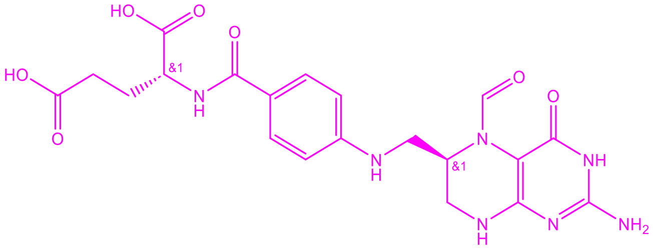	NC1=NC2=C(N([C@@H](CN2)CNC3=CC=C(C=C3)C(N[C@@H](C(O)=O)CCC(O)=O)=O)C=O)C(N1)=O	ZINC000009212427

234	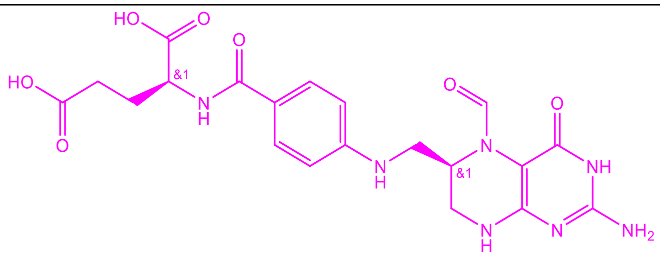	NC1=NC2=C(N([C@@H](CN2)CNC3=CC=C(C=C3)C(N[C@H](C(O)=O)CCC(O)=O)=O)C=O)C(N1)=O	ZINC000009212428
235	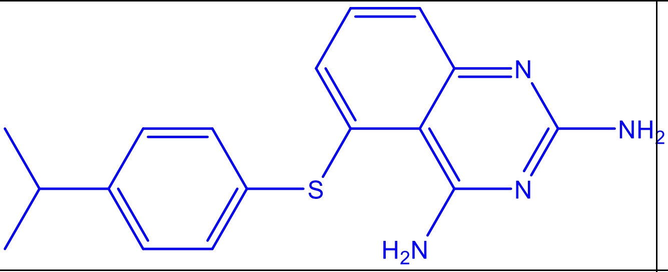	CC(C1=CC=C(C=C1)SC2=CC=CC3=NC(N)=NC(N)=C23)C	ZINC000005882454
236	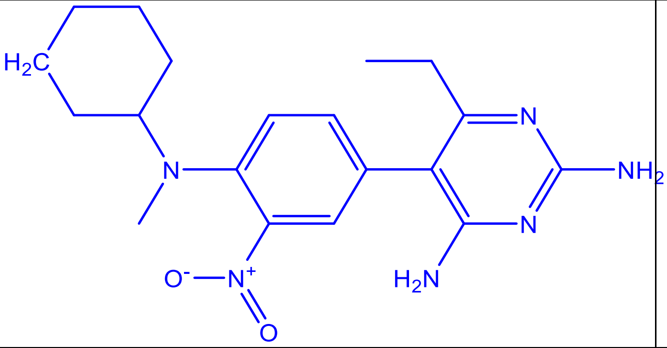	CCC1=NC(N)=NC(N)=C1C2=CC=C(C([N+]([O-])=O)=C2)N(C3CCCCC3)C	ZINC000005479327
237	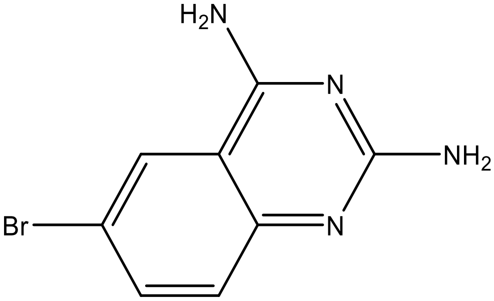	NC1=NC(N)=C2C=C(C=CC2=N1)Br	ZINC000005974291
238	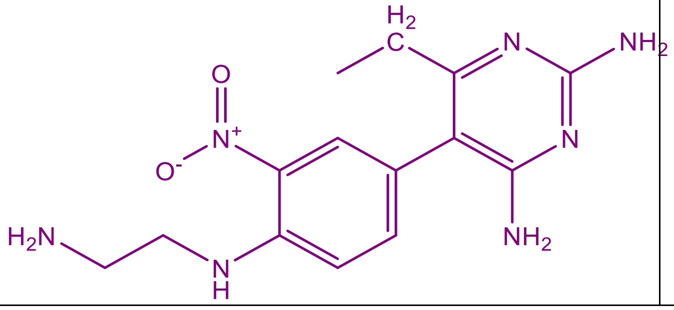	CCC1=NC(N)=NC(N)=C1C2=CC=C(C([N+]([O-])=O)=C2)NCCN	ZINC000026501194

239	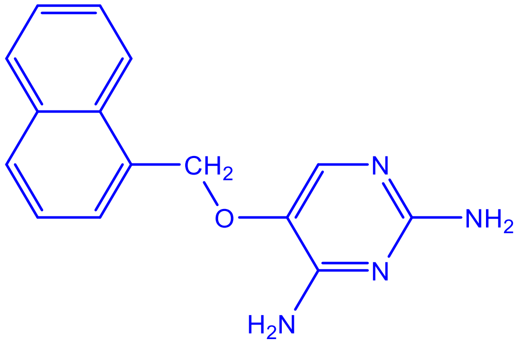	NC1=NC=C(C(N)=N1)OCC2=CC=CC3=CC=CC=C23	ZINC000013532422
240	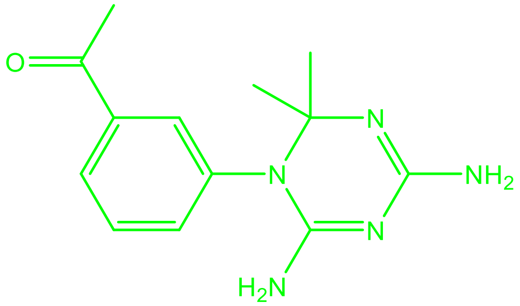	CC(C1=CC=CC(N2C(N)=NC(N)=NC(C)2C)=C1)=O	ZINC000025968625
241	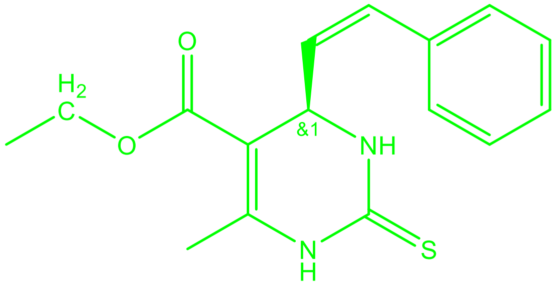	CCOC(C1=C(NC(N[C@@H]1/C=C\C2=CC=CC=C2)=S)C)=O	ZINC000015220490
242	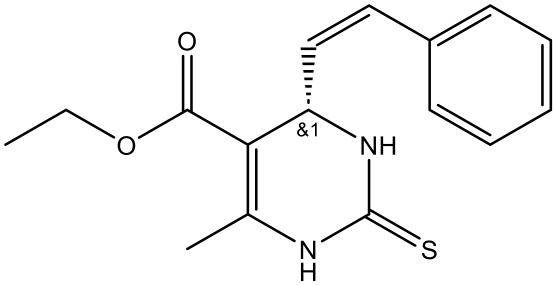	CCOC(C1=C(NC(N[C@H]1/C=C\C2=CC=CC=C2)=S)C)=O	ZINC000015220492
243	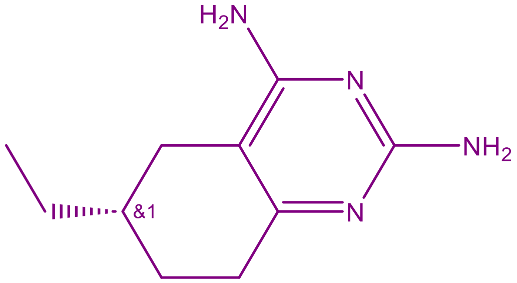	CC[C@H]1CCC2=NC(N)=NC(N)=C2C1	ZINC000005885245

244	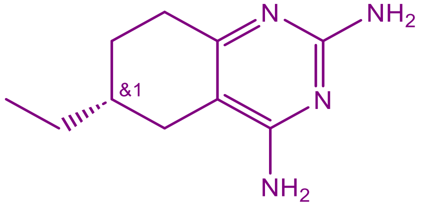	CC[C@@H]1CCC2=NC(N)=NC(N)=C2C1	ZINC000005885888
245	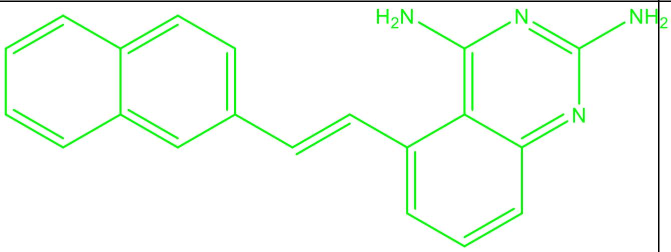	NC1=NC(N)=C2C(/C=C/C3=CC=C4C=CC=CC4=C3)=CC=CC2=N1	ZINC000025966783
246	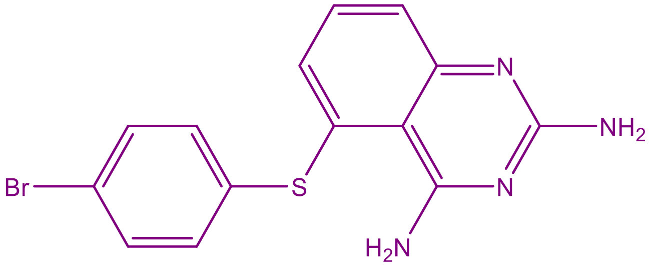	NC1=NC(N)=C2C(C=CC=C2SC3=CC=C(C=C3)Br)=N1	ZINC000005974267
247	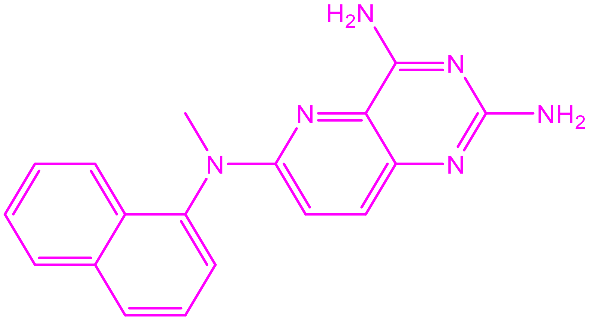	CN(C1=CC=CC2=CC=CC=C12)C3=CC=C4N=C(N=C(C4=N3)N)N	ZINC000013781977
248	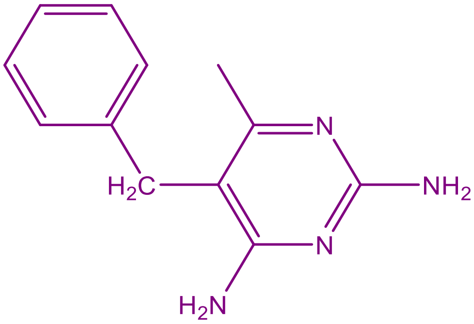	CC1=NC(N)=NC(N)=C1CC2=CC=CC=C2	ZINC000013532464
249	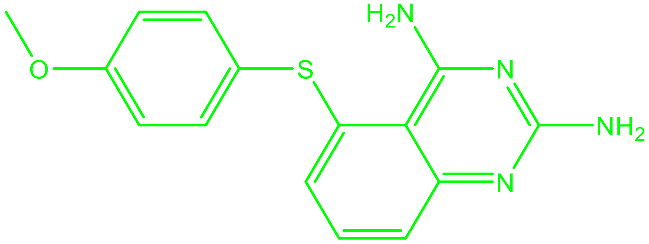	COC1=CC=C(C=C1)SC2=CC=CC3=NC(N)=NC(N)=C23	ZINC000005889226

250	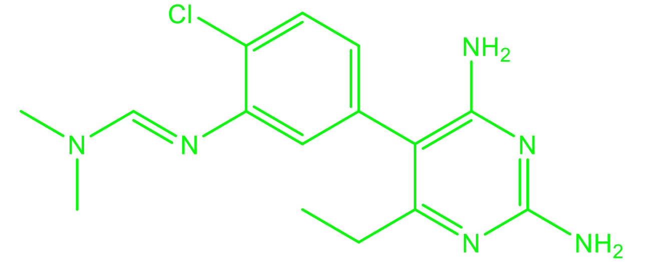	CCC1=NC(N)=NC(N)=C1C2=CC=C(C(/N=C/N(C)C)=C2)Cl	ZINC000018102337
251	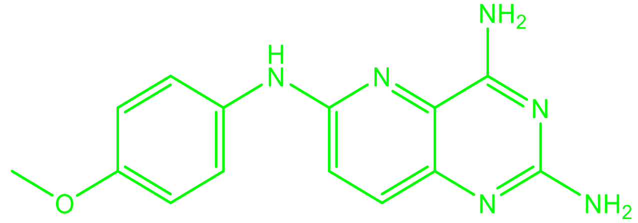	COC1=CC=C(C(C=C1)NC2=CC=C3N=C(N=C(C3=N2)N)N)N	ZINC000005854014
252	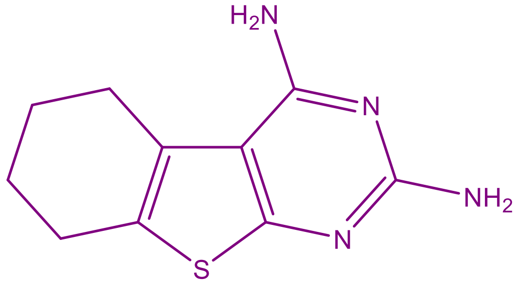	NC1=NC2=C(C(N)=N1)C(CCCC3)=C3S2	ZINC000005847275
253	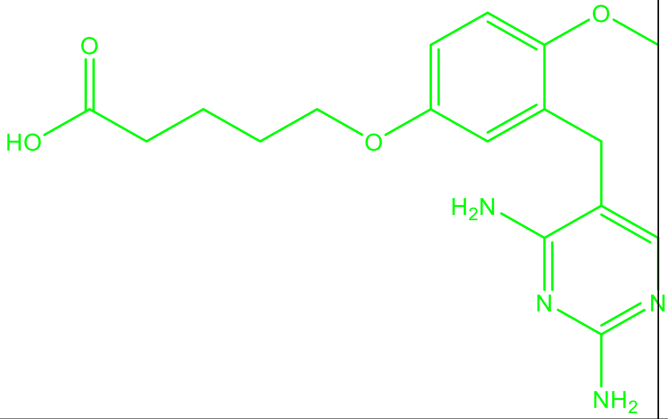	COC1=CC=C(C=C1CC2=CN=C(N=C2N)N)OCCCCC(O)=O	ZINC000024799342
254	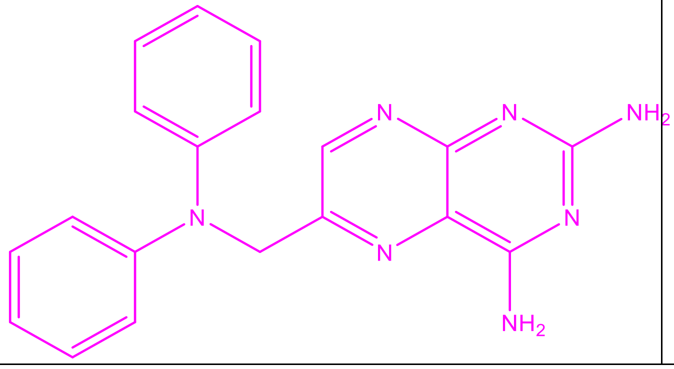	NC1=NC(N)=C2N=C(C=NC2=N1)CN(C3=CC=CC=C3)C4=CC=CC=C4	ZINC000005934340

255	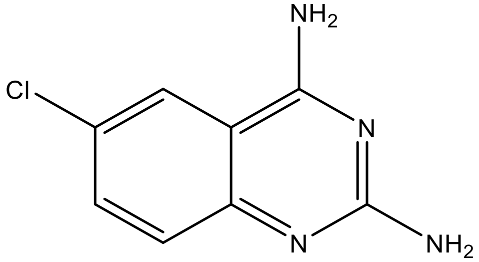	NC1=NC(N)=C2C=C(C=CC2=N1)Cl	ZINC000013284775
256	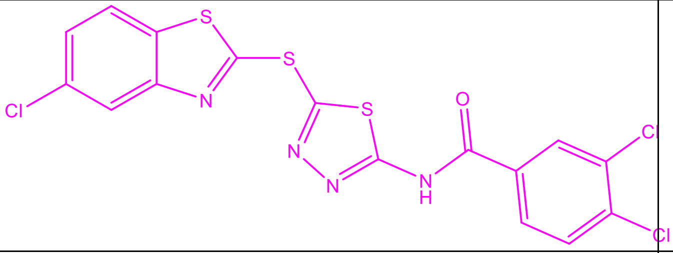	O=C(C1=CC=C(C(Cl)=C1)Cl)NC2=NN=C(S2)SC3=NC4=CC(Cl)=CC=C4S3	ZINC000013284775
257	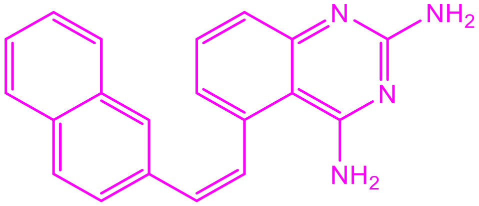	NC1=NC(N)=C2C(/C=C\C3=CC=C4C=CC=CC4=C3)=CC=CC2=N1	ZINC000025971101
258	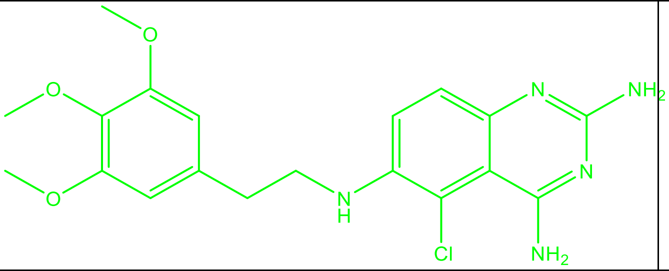	COC1=CC(CCNC2=CC=C3N=C(N=C(C3=C2Cl)N)N)=CC(OC)=C1OC	ZINC000005888671
259	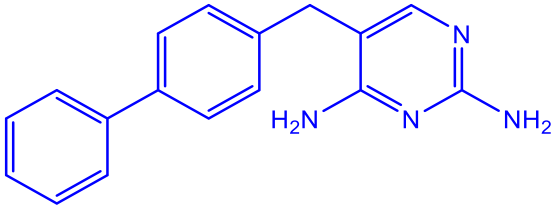	NC1=NC=C(C(N)=N1)CC2=CC=C(C=C2)C3=CC=CC=C3	ZINC000013726753
260	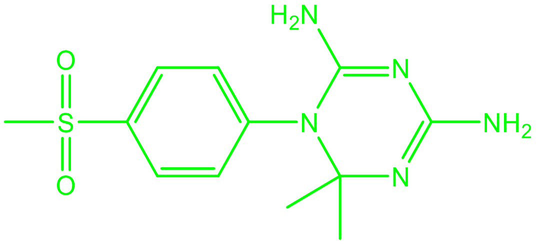	CC1(N=C(N=C(N1C2=CC=C(C=C2)S(C)(=O)=O)N)N)C	ZINC000001842836

261	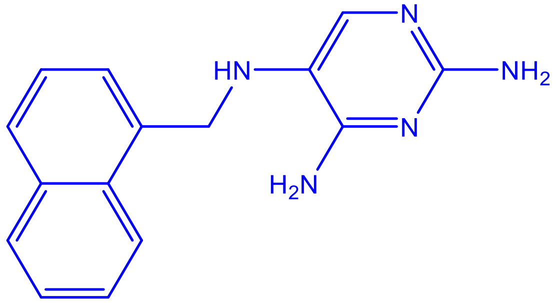	NC1=NC=C(C(N)=N1)NC<br>C2=CC=CC3=CC=CC=C2<br>3	ZINC000<br>01353245<br>2
262	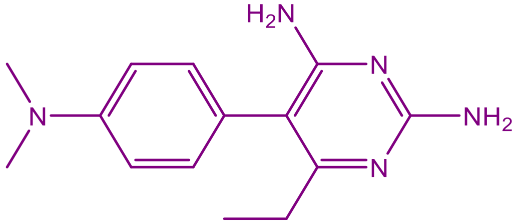	CCC1=NC(N)=NC(N)=C1<br>C2=CC=C(C=C2)N(C)C	ZINC000<br>01321247<br>6
263	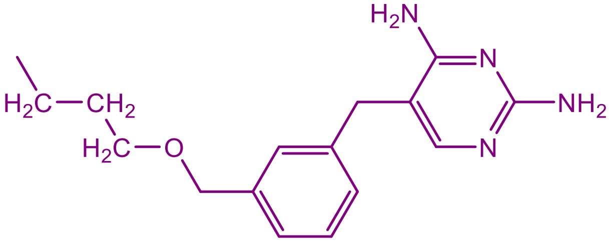	CCCCOCC1=CC=CC(CC2<br>=CN=C(N=C2N)N)=C1	ZINC000<br>01372671<br>1
264	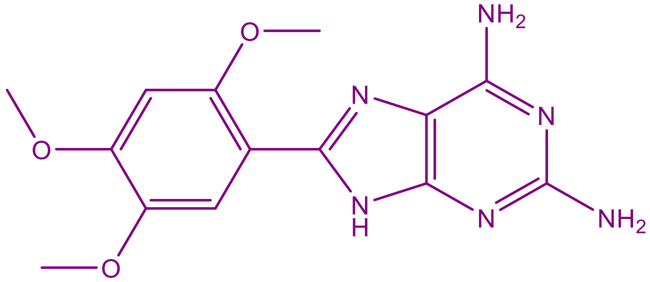	COC1=CC(OC)=C(C=C1O<br>C)C2=NC3=C(N2)N=C(N<br>=C3N)N	ZINC000<br>00588854<br>6
265	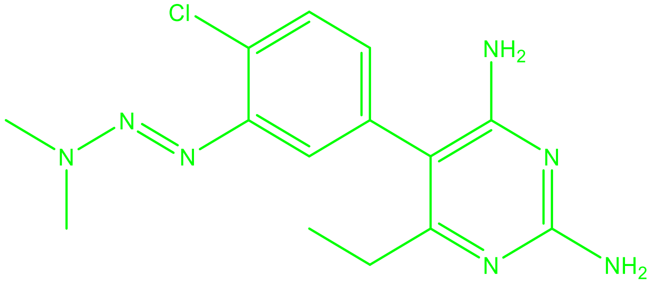	CCC1=NC(N)=NC(N)=C1<br>C2=CC=C(C(/N=N/N(C)C)<br>=C2)Cl	ZINC000<br>00157481<br>9
266	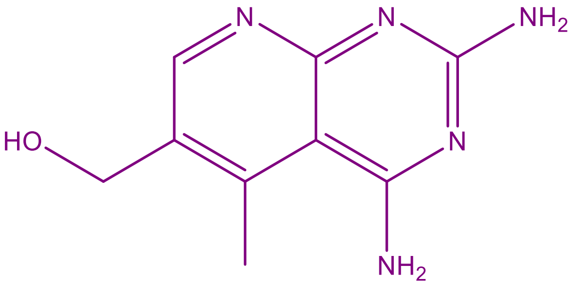	CC1=C(C=NC2=NC(N)=N<br>C(N)=C12)CO	ZINC000<br>00589152<br>8

267	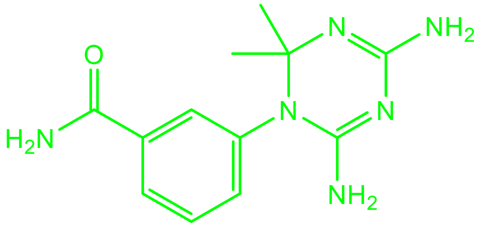	CC1(N=C(N=C(N1C2=CC=CC(C(N)=O)=C2)N)N)C	ZINC000013740714
268	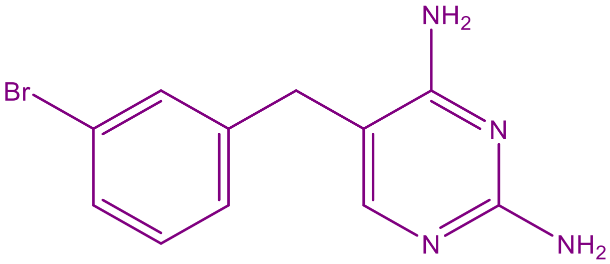	NC1=NC=C(C(N)=N1)CC2=CC=CC(Br)=C2	ZINC000013726756
269	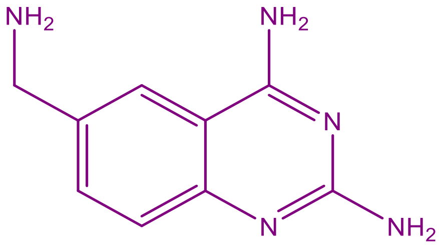	NCC1=CC=C2N=C(N=C(C2=C1)N)N	ZINC000025968986
270	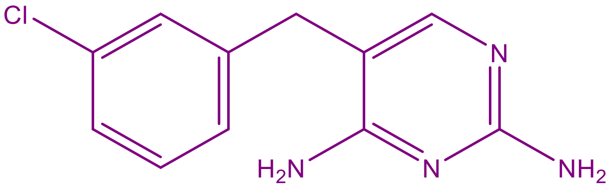	NC1=NC=C(C(N)=N1)CC2=CC=CC(Cl)=C2	ZINC000013726726
271	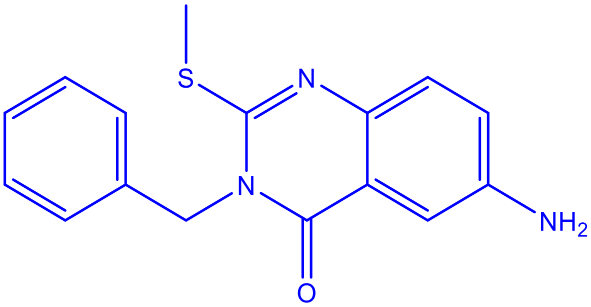	CSC1=NC2=CC=C(C=C2C(N1CC3=CC=CC=C3)=O)N	ZINC000049111297

Table S2 Ligands used for screening of top hit compounds using AD4 and Autodock vina.

Ligands	AD4 score	Vina score	Ligands	AD4 score	Vina score
**1**	-10.45	-10.40	**139**	-7.34	-7.50
**2**	-10.41	-10.40	**140**	-9.23	-9.30
**3**	-10.27	-10.30	**141**	-9.23	-9.30
**4**	-9.65	-9.90	**142**	-10.45	-10.40
**5**	-10.21	-10.50	**143**	-8.34	-8.50

**6**	-10.03	-10.40	**144**	-8.43	-8.60
**7**	-9.07	-9.40	**145**	-10.45	-10.10
**8**	-10.41	-10.40	**146**	-8.43	-8.60
**9**	-11.36	-11.20	**147**	-7.23	-7.70
**10**	-10.89	-10.80	**148**	-9.87	-9.10
**11**	-8.75	-8.70	**149**	-9.67	-9.80
**12**	-9.87	-9.80	**150**	-9.45	-8.90
**13**	-10.56	-10.40	**151**	-9.34	-8.60
**14**	-10.12	-10.20	**152**	-10.45	-10.00
**15**	-6.98	-7.60	**153**	-7.32	-7.00
**16**	-9.12	-10.20	**154**	-9.32	-9.30
**17**	-7.89	-8.90	**155**	-7.87	-7.50
**18**	-10.52	-10.50	**156**	-8.90	-8.50
**19**	-9.23	-9.20	**157**	-11.01	-10.20
**20**	-8.81	-8.80	**158**	-9.94	-8.90
**21**	-10.23	-10.00	**159**	-9.95	-9.70
**22**	-8.87	-8.90	**160**	-7.43	-7.60
**23**	-6.98	-6.90	**161**	-8.43	-8.60
**24**	-9.95	-9.90	**162**	-8.32	-8.40
**25**	-8.23	-8.00	**163**	-8.83	-8.80
**26**	-10.23	-10.70	**164**	-8.23	-8.80
**27**	-11.78	-11.60	**165**	-8.03	-8.00
**28**	-9.56	-9.70	**166**	-9.13	-9.20
**29**	-10.12	-10.40	**167**	-8.32	-8.60
**30**	-10.02	-10.00	**168**	-8.17	-8.10
**31**	-7.98	-8.50	**169**	-9.12	-9.00
**32**	-8.45	-8.50	**170**	-8.03	-7.90
**33**	-8.62	-8.60	**171**	-9.65	-9.50
**34**	-7.60	-7.60	**172**	-10.21	-10.10
**35**	-8.16	-8.70	**173**	-8.32	-8.40
**36**	-9.03	-9.00	**174**	-10.21	-10.20
**37**	-10.51	-10.50	**175**	-7.32	-7.60
**38**	-10.74	-10.80	**176**	-8.21	-8.30
**39**	-10.41	-10.00	**177**	-8.34	-8.20
**40**	-9.31	-9.30	**178**	-7.43	-7.60
**41**	-11.58	-11.00	**179**	-7.67	-7.50
**42**	-10.36	-10.50	**180**	-11.23	-11.10
**43**	-9.78	-9.90	**181**	-7.32	-7.30
**44**	-9.45	-9.60	**182**	-8.60	-8.40
**45**	-10.15	-10.20	**183**	-10.76	-10.70
**46**	-7.47	-7.80	**184**	-9.50	-9.50
**47**	-7.56	-7.70	**185**	-7.43	-7.50
**48**	-9.75	-9.80	**186**	-8.23	-8.20
**49**	-8.19	-8.20	**187**	-7.70	-7.70
**50**	-7.62	-7.60	**188**	-6.93	-6.90
**51**	-10.56	-10.60	**189**	-7.93	-7.90
**52**	-10.15	-10.40	**190**	-7.32	-7.40
**53**	-7.56	-7.70	**191**	-7.89	-7.80
**54**	-6.43	-7.70	**192**	-8.50	-8.50

**55**	-7.23	-8.10	**193**	-8.56	-8.90
**56**	-7.98	-8.60	**194**	-7.21	-7.20
**57**	-9.89	-10.90	**195**	-8.70	-8.70
**58**	-7.56	-7.50	**196**	-7.45	-7.40
**59**	-8.78	-8.60	**197**	-9.87	-9.40
**60**	-10.44	-10.20	**198**	-8.98	-8.00
**61**	-10.34	-10.70	**199**	-6.54	-6.90
**62**	-9.87	-9.80	**200**	-8.45	-8.20
**63**	-9.54	-9.60	**201**	-7.93	-7.90
**64**	-10.45	-10.50	**202**	-7.80	-7.80
**65**	-10.67	-10.50	**203**	-8.65	-8.40
**66**	-9.67	-9.50	**204**	-8.93	-8.90
**67**	-9.54	-9.40	**205**	-8.02	-8.10
**68**	-11.34	-11.00	**206**	-7.32	-7.70
**69**	-10.23	-10.20	**207**	-9.21	-9.70
**70**	-10.23	-10.50	**208**	-7.23	-7.10
**71**	-7.34	-7.40	**209**	-8.98	-8.70
**72**	-8.80	-8.10	**210**	-8.32	-8.20
**73**	-9.56	-9.40	**211**	-7.93	-7.90
**74**	-11.56	-11.00	**212**	-9.43	-9.60
**75**	-8.32	-8.30	**213**	-7.67	-7.90
**76**	-8.54	-8.50	**214**	-6.32	-6.60
**77**	-10.56	-10.40	**215**	-8.93	-8.90
**78**	-8.65	-8.60	**216**	-8.49	-8.60
**79**	-8.32	-8.30	**217**	-9.32	-9.30
**80**	-10.56	-10.50	**218**	-5.20	-8.20
**81**	-10.56	-10.10	**219**	-7.40	-7.40
**82**	-9.69	-9.50	**220**	-7.73	-7.80
**83**	-9.45	-9.10	**221**	-9.32	-9.00
**84**	-8.23	-8.10	**222**	-8.76	-8.70
**85**	-11.75	-11.60	**223**	-7.43	-7.70
**86**	-9.56	-9.70	**224**	-8.60	-8.60
**87**	-7.45	-8.60	**225**	-9.21	-9.20
**88**	-7.98	-8.80	**226**	-9.40	-9.40
**89**	-9.87	-9.60	**227**	-8.30	-8.30
**90**	-10.34	-10.80	**228**	-9.50	-9.50
**91**	-8.45	-8.10	**229**	-8.61	-8.40
**92**	-8.47	-8.30	**230**	-7.70	-7.70
**93**	-10.43	-10.60	**231**	-7.21	-7.20
**94**	-9.98	-9.90	**232**	-8.40	-8.80
**95**	-10.32	-10.30	**233**	-10.30	-10.30
**96**	-7.34	-7.70	**234**	-10.39	-10.00
**97**	-9.56	-9.50	**235**	-9.32	-9.70
**98**	-8.45	-7.70	**236**	-10.10	-9.90
**99**	-11.76	-11.60	**237**	-6.80	-6.80
**100**	-10.43	-10.00	**238**	-7.20	-7.10
**101**	-7.76	-7.90	**239**	-8.32	-9.30
**102**	-10.45	-10.20	**240**	-7.89	-8.20
**103**	-10.32	-10.30	**241**	-8.32	-8.40

**104**	-8.30	-8.30	**242**	-6.32	-6.80
**105**	-7.45	-7.80	**243**	-7.01	-7.00
**106**	-9.45	-9.50	**244**	-7.90	-7.00
**107**	-9.87	-9.80	**245**	-8.61	-8.60
**108**	-9.45	-9.90	**246**	-7.60	-7.70
**109**	-9.56	-9.30	**247**	-10.81	-10.40
**110**	-7.78	-7.70	**248**	-7.21	-7.00
**111**	-9.98	-9.90	**249**	-8.60	-8.60
**112**	-10.02	-10.00	**250**	-8.31	-8.30
**113**	-9.17	-9.00	**251**	-8.80	-8.80
**114**	-10.06	-10.10	**252**	-7.82	-7.50
**115**	-8.09	-8.00	**253**	-8.14	-8.10
**116**	-8.56	-8.80	**254**	-10.81	-10.80
**117**	-10.03	-10.10	**255**	-6.43	-6.60
**118**	-9.70	-8.30	**256**	-10.22	-10.20
**119**	-10.22	-10.40	**257**	-10.03	-10.00
**120**	-8.23	-8.00	**258**	-8.80	-8.80
**121**	-9.65	-9.60	**259**	-9.40	-9.40
**122**	-7.98	-7.90	**260**	-8.90	-8.70
**123**	-9.02	-9.00	**261**	-9.92	-9.50
**124**	-8.43	-8.50	**262**	-7.81	-7.70
**125**	-6.98	-7.00	**263**	-8.60	-7.60
**126**	-8.32	-8.00	**264**	-7.70	-7.90
**127**	-10.21	-10.30	**265**	-8.10	-8.50
**128**	-9.23	-9.70	**266**	-7.10	-7.10
**129**	-9.91	-9.90	**267**	-7.09	-8.20
**130**	-10.89	-10.60	**268**	-7.07	-7.80
**131**	-9.32	-9.80	**269**	-5.66	-7.10
**132**	-10.32	-10.00	**270**	-6.10	-7.60
**133**	-8.12	-8.10	**271**	-6.32	-9.00
**134**	-7.12	-7.60			
**135**	-7.34	-7.60			
**136**	-7.32	-7.80			
**137**	-8.21	-8.40			
**138**	-9.12	-9.20			

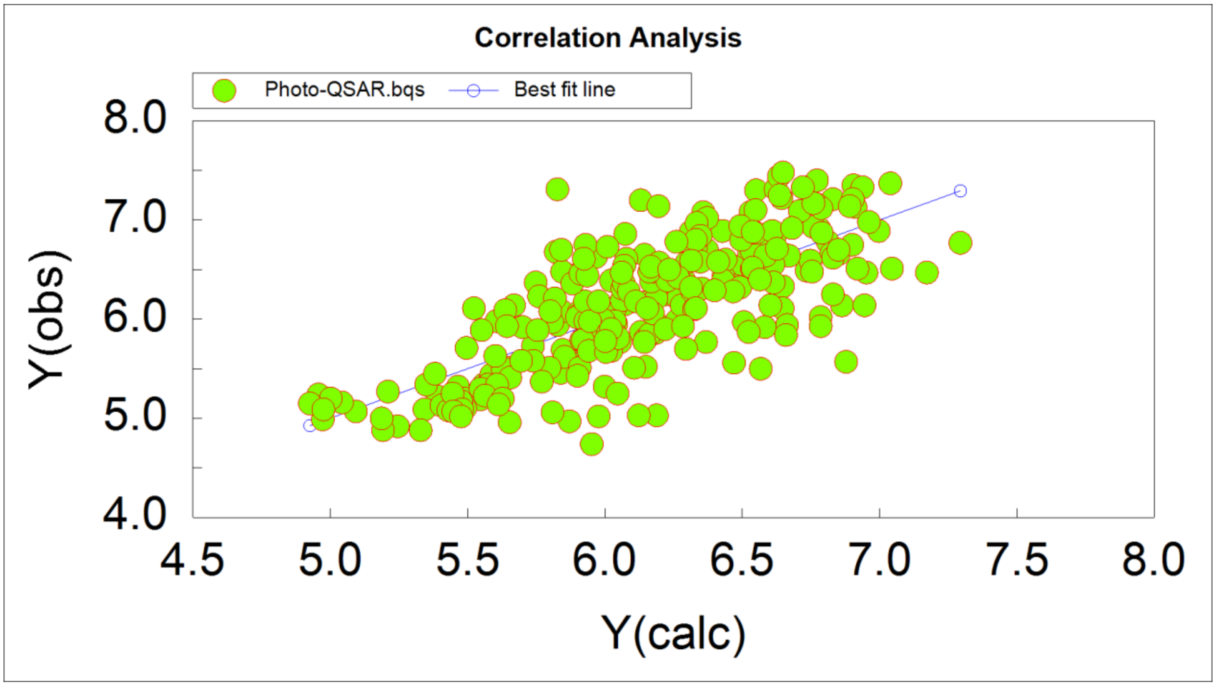

***Figure S*1:** Deep learning based QSAR model for method validation and correlation analysis.

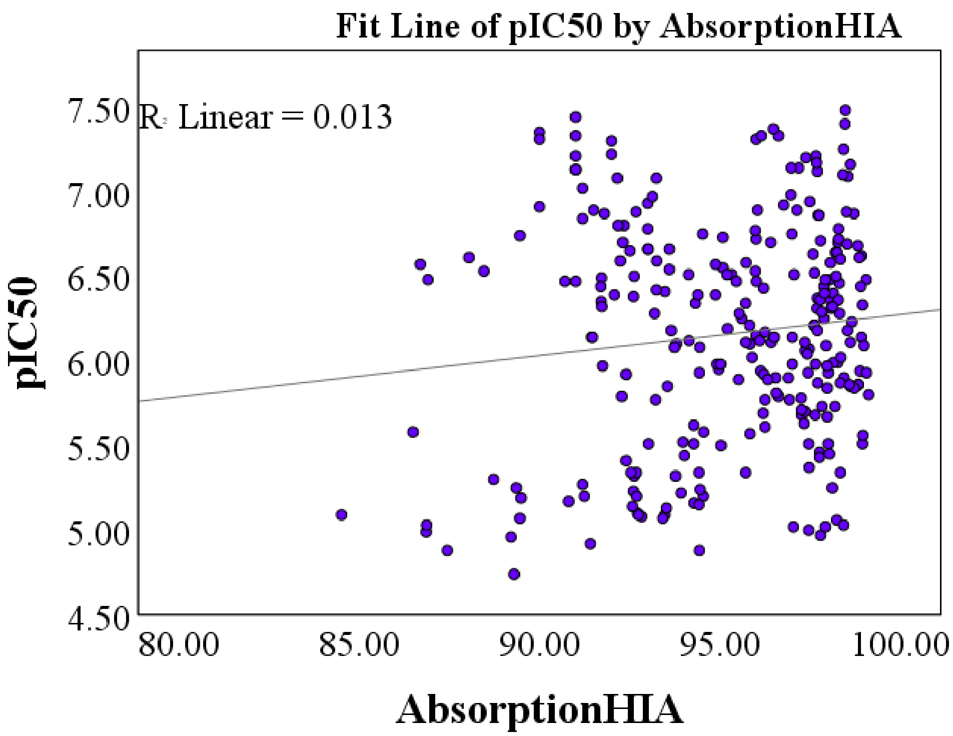

***Figure S*2**: Deep learning based QSAR *model* for absorption HIA prediction.

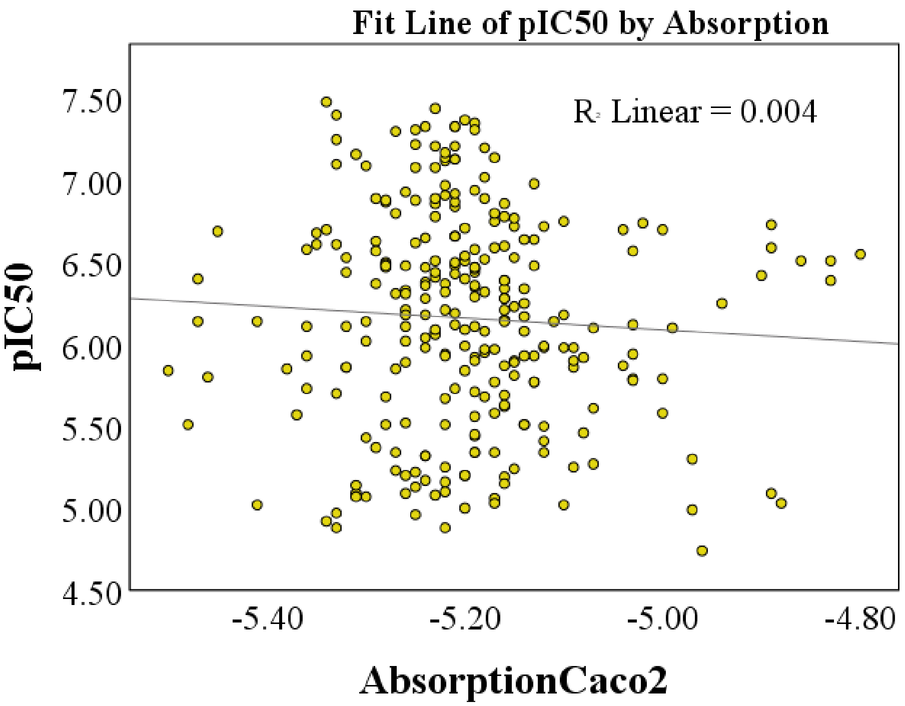

***Figure S*3:** Deep learning based QSAR *model* for absorption Caco2 prediction.

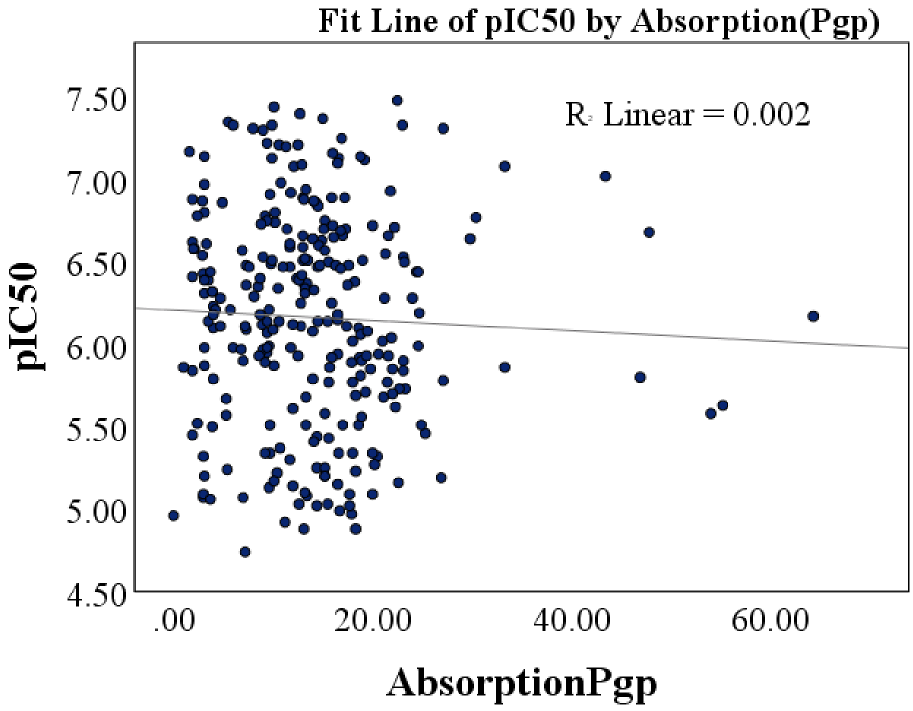

Figure S4: Deep learning based QSAR *model* for absorption Pgp prediction.

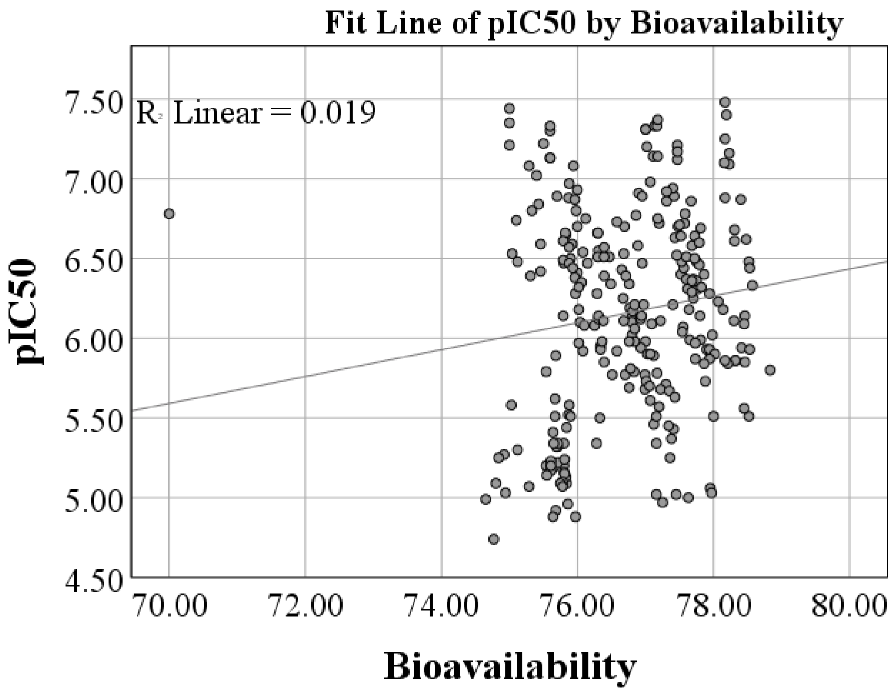

***Figure S*5:** Deep learning based QSAR *model* for bioavailability prediction.

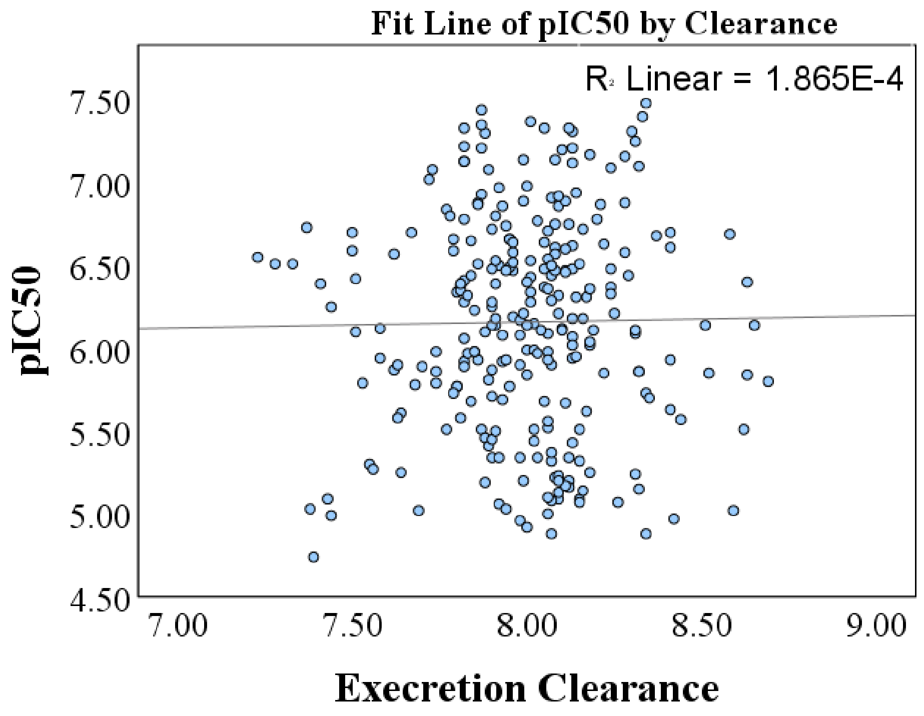

Figure S6: Deep learning based QSAR *model* for excretion clearance prediction.

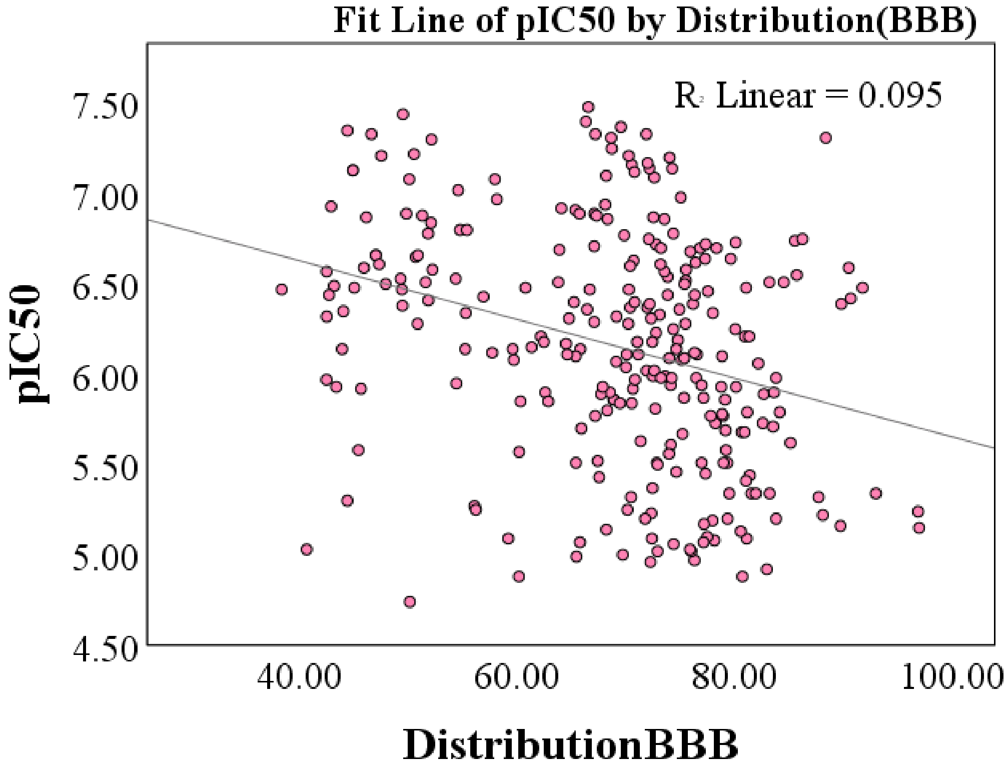

***Figure S*7:** Deep learning based QSAR *model* for distribution prediction.

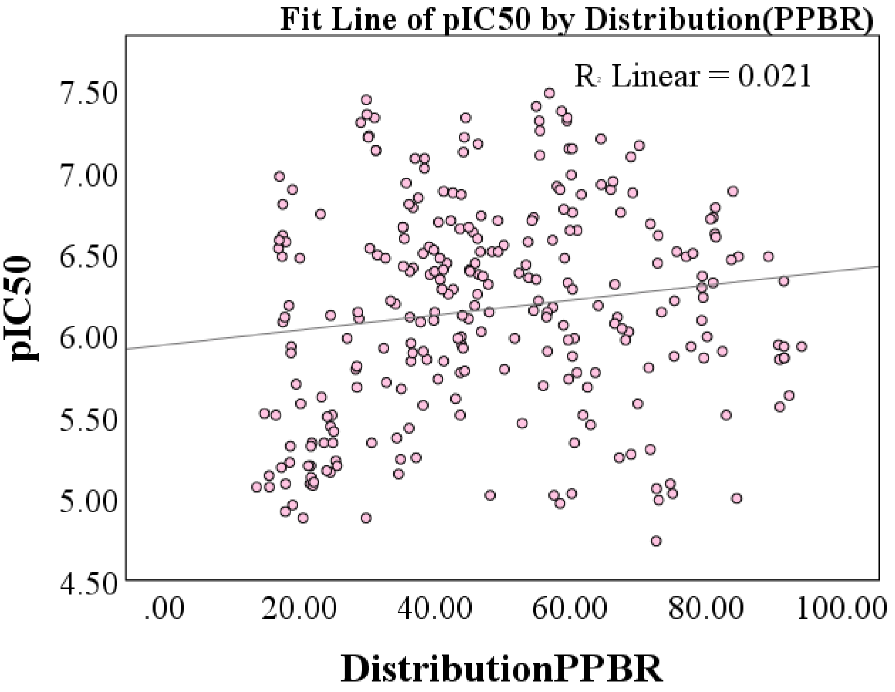

Figure S8: Deep learning based QSAR *model* for distribution PPBR prediction.

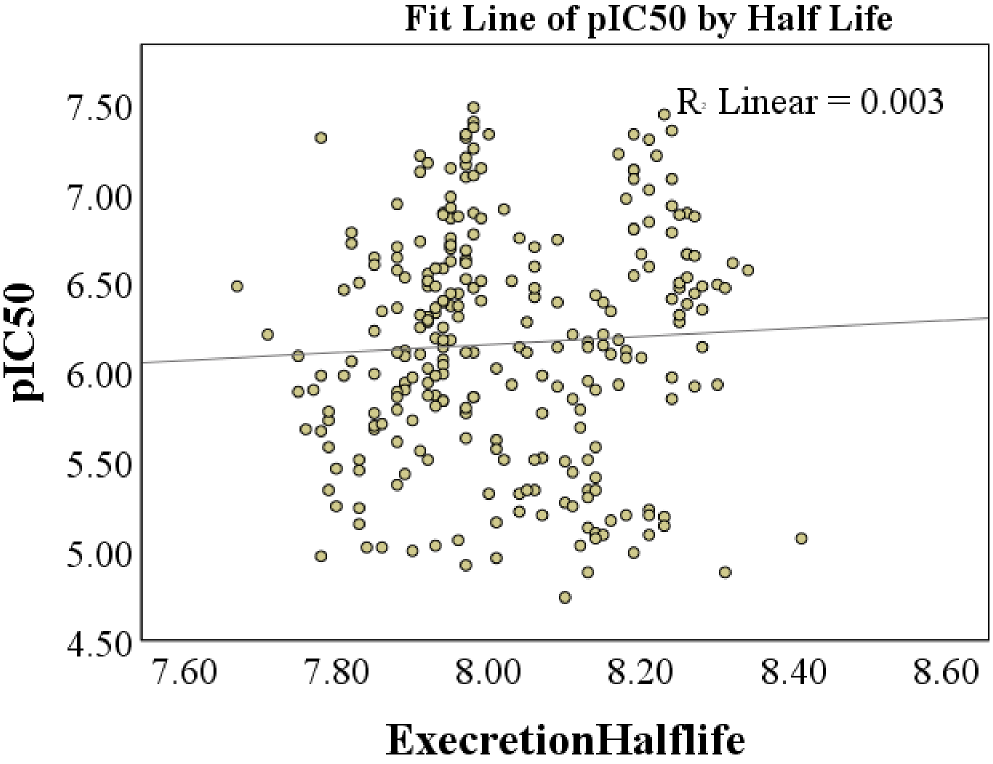

Figure S9: Deep learning based QSAR *model* for excretion Half-life prediction.

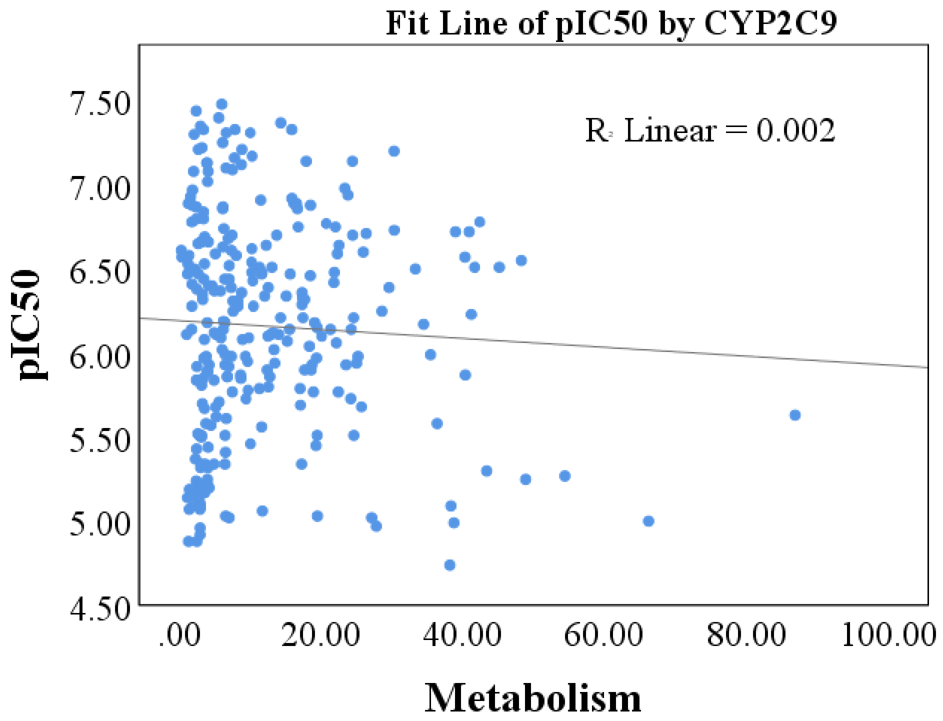

Figure *S*10: Deep learning based QSAR *model* for metabolism by CYP2C9 prediction.

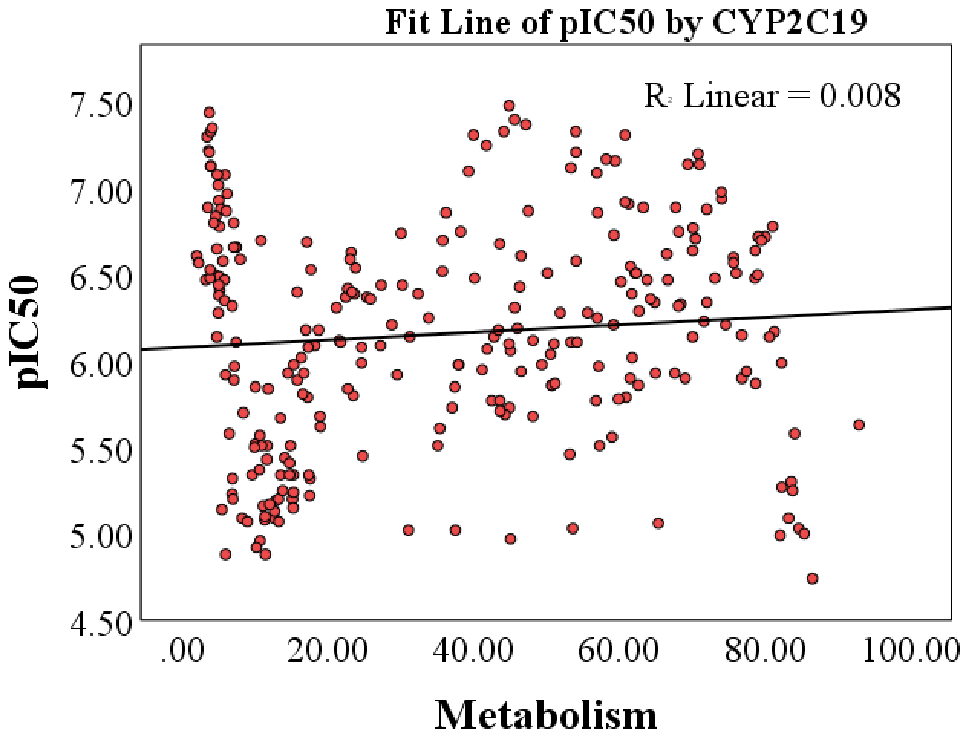

***Figure S*11:** Deep learning based QSAR *model* for metabolism by CYP2C19 prediction.

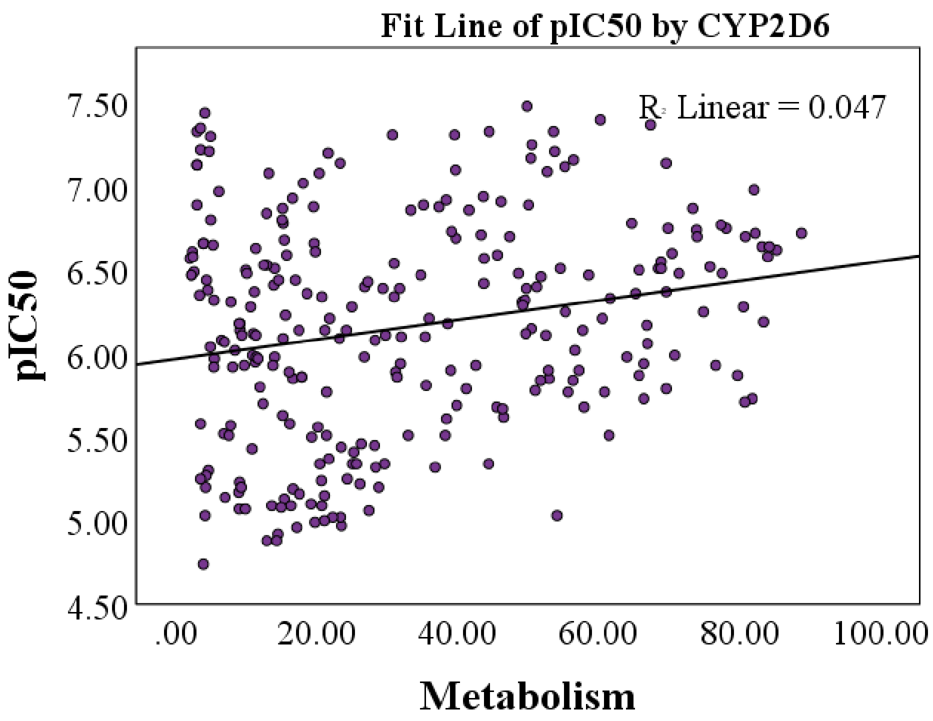

***Figure S*12:** Deep learning based QSAR *model* for metabolism by CYP2D6 prediction.

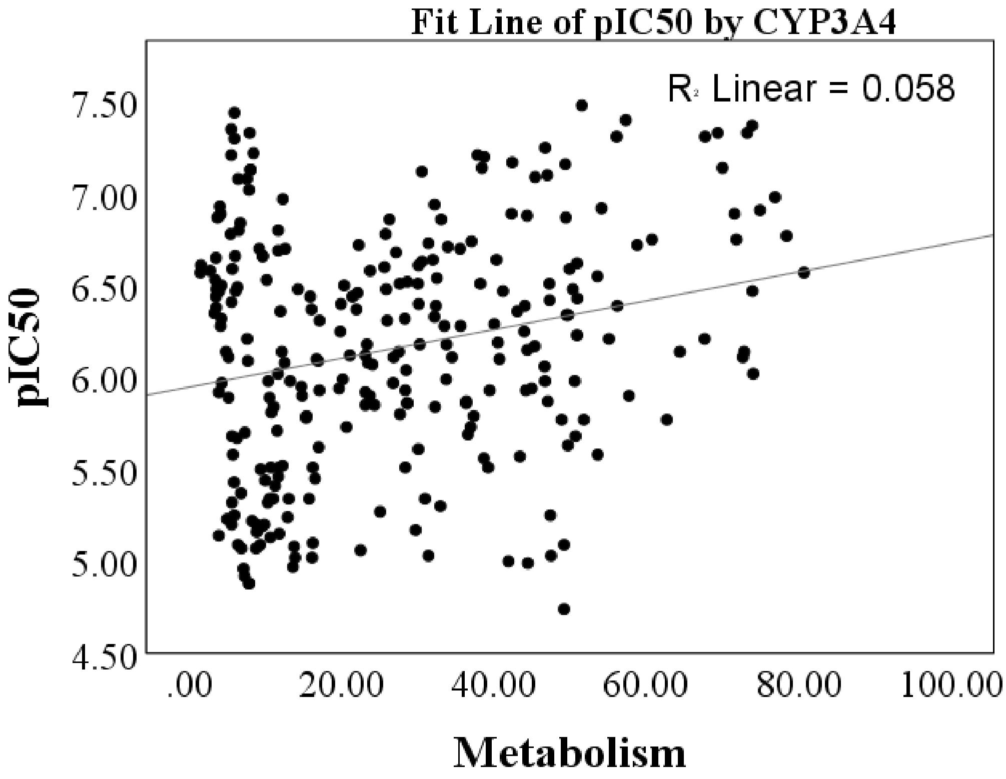

***Figure S*13:** Deep learning based QSAR *model* for metabolism by CYP3A4 prediction.

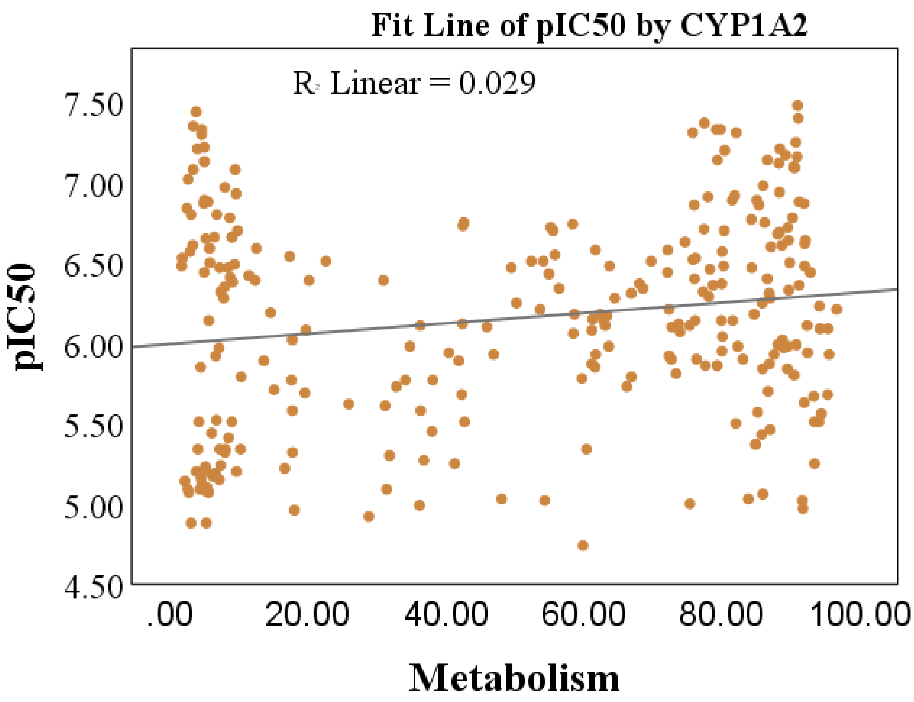

*Figure S*14: Deep learning based QSAR *model* for metabolism by CYP1A2 prediction.

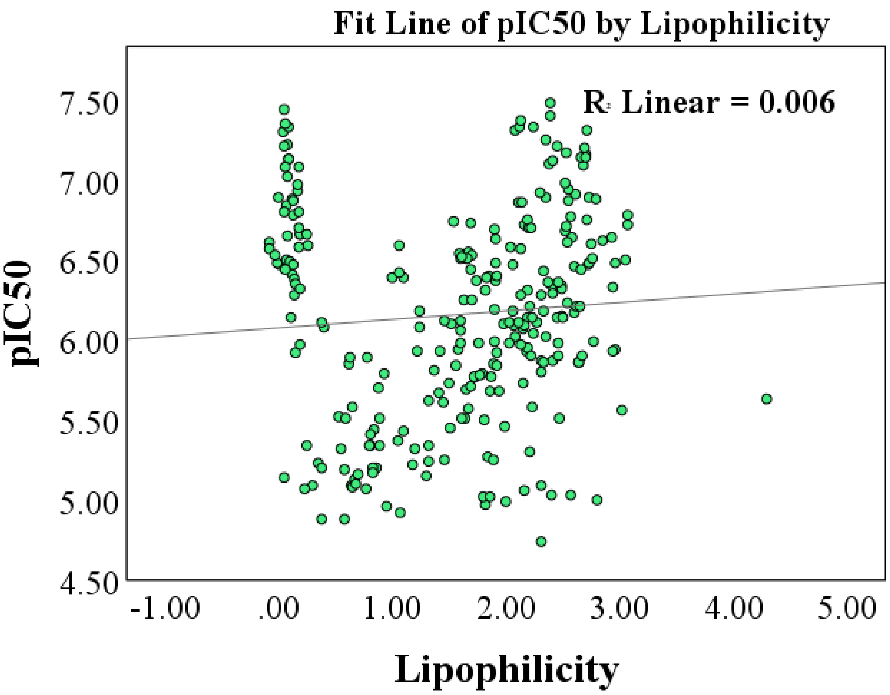

*Figure S*15: Deep learning based QSAR *model* for lipophilicity prediction.

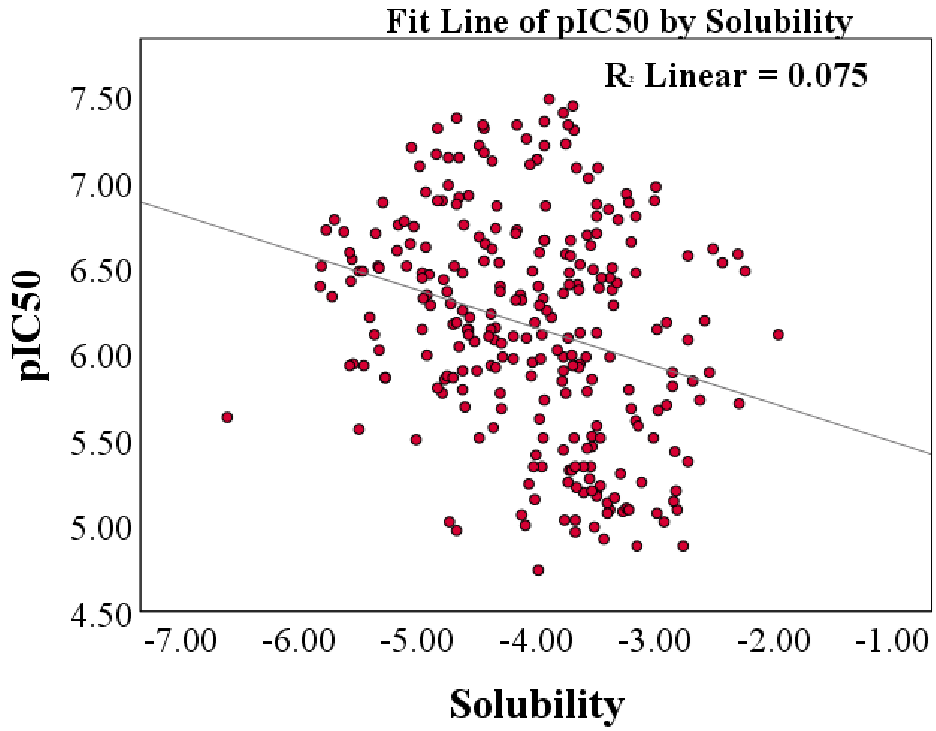

Figure S16: Deep learning based QSAR model for solubility prediction.

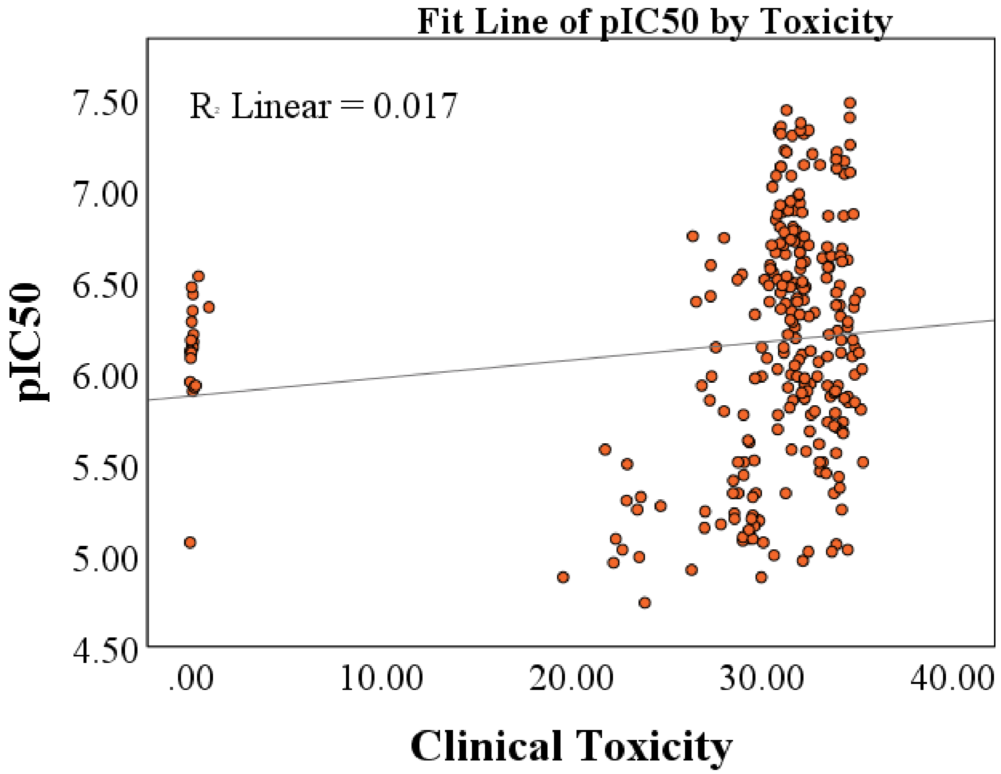

Figure S17*:* Deep learning based QSAR *model* for clinical toxicity prediction.

Table S3 Optimized XYZ cartesian data for all the top-hit lead compounds used in the study.

Cartesian Coordinates for top-hit compound **9** optimized in the gas phase using DFT/B3LYP/6-311+g(d,p)/GD3.

N	11.94998900	-2.47432300	0.56633800
C	10.72646000	-1.91126500	0.39918300
N	9.70300000	-2.57140800	1.01214300
C	8.49273700	-2.07205500	0.86032700
N	7.44319800	-2.68186600	1.44962100
C	8.27517800	-0.87856500	0.07659300
N	7.03656200	-0.37053100	-0.07856200
C	6.92335500	0.72142200	-0.80920800
C	5.56575800	1.35487100	-1.01231300
N	4.52014800	0.53802700	-0.45653100
C	3.20375800	0.92010800	-0.37911300
C	2.27976200	0.10981100	0.31479400
C	0.94624000	0.46832400	0.39866100
C	0.47139400	1.64368300	-0.20513300
C	-0.94914800	2.08615300	-0.15979100
O	-1.41444100	2.89517700	-0.95426000
N	-1.73896000	1.53517000	0.82570800
C	-3.11927900	1.98160500	0.98979800
C	-4.04678300	1.47641300	-0.12718400
C	-5.52177600	1.44327200	0.28260800
C	-6.41042300	0.97152500	-0.87222600
N	-7.81484600	0.85638100	-0.48563100
C	-8.48594800	-0.28840000	-0.18520200
O	-9.55849900	-0.30294900	0.39840400
C	-7.92478500	-1.58134100	-0.74259200
C	-8.12109700	-1.84009800	-2.09950400
C	-7.76850400	-3.07125600	-2.64755400
C	-7.22158400	-4.06509900	-1.83797700
C	-7.02561100	-3.82045400	-0.48318100
C	-7.36312300	-2.57924000	0.07011000
C	-7.06859400	-2.28945500	1.49854700
O	-6.90398200	-1.18536400	1.96330600
O	-6.96419800	-3.41174100	2.25356100
C	-3.21668000	3.50476600	1.16494600
O	-4.09856900	4.20220800	0.73757000
O	-2.21535100	3.97764200	1.94552400
C	1.38380100	2.43541400	-0.90984900
C	2.72547500	2.09435300	-0.99481200
C	8.07551200	1.31262300	-1.39361100

N	9.29117400	0.83888600	−1.25552200
C	9.42900800	−0.29078700	−0.50363400
N	10.65996200	−0.80840900	−0.33957600
H	12.05440300	−3.24749400	1.20003900
H	12.75809200	−1.98842200	0.21629200
H	6.51516700	−2.31890900	1.31439000
H	7.59369200	−3.52866200	1.97197000
H	5.57601800	2.35738500	−0.55453000
H	5.40821100	1.51155700	−2.08885500
H	4.83810000	−0.24053900	0.10079100
H	2.62202100	−0.81184000	0.77451800
H	0.26342700	−0.19887600	0.91311300
H	−1.27632800	1.17969700	1.64700700
H	−3.45941300	1.56032800	1.94279500
H	−3.89748200	2.10021600	−1.01047400
H	−3.71599300	0.46502400	−0.38331100
H	−5.66983600	0.76655900	1.12896000
H	−5.84008900	2.44174800	0.59383600
H	−6.33100700	1.66508200	−1.71611500
H	−6.07799700	−0.00412200	−1.23104200
H	−8.24983500	1.68369800	−0.09579500
H	−8.56473900	−1.07308500	−2.72391400
H	−7.92758700	−3.25539700	−3.70402500
H	−6.94951100	−5.02571700	−2.25915600
H	−6.59950400	−4.58383400	0.15458200
H	−6.75785700	−3.12517500	3.15526500
H	−2.33584600	4.93630300	2.01329900
H	1.01721200	3.33274500	−1.39353800
H	3.40020500	2.73766700	−1.54496600
H	7.96459400	2.21281500	−1.99599600

Cartesian Coordinates for top-hit compound **27** optimized in the gas phase using DFT/B3LYP/6-311+g(d,p)/GD3.

N	5.98670700	−2.45539800	−0.28846600
C	4.96171700	−1.56723100	−0.17044700
N	4.99837900	−0.79234000	0.94725800
C	3.99942000	0.04760400	1.13327900
N	4.03050900	0.80915500	2.26141200
C	2.90357700	0.13770500	0.19865700
C	1.78847300	0.97878800	0.35697300
C	0.81614200	1.00800400	−0.61832000
S	−0.61089900	2.07285600	−0.39339700
O	−0.30478300	2.97936700	0.72284100
O	−1.00743000	2.58160000	−1.71214200

C	-1.90192900	0.94341900	0.16053600
C	-1.92008100	0.53825400	1.51573800
C	-2.88292400	-0.34377700	1.93469300
C	-3.86029600	-0.84599000	1.03281500
C	-4.86778800	-1.75652200	1.44191900
C	-5.80333300	-2.21794500	0.54609200
C	-5.77846600	-1.78896100	-0.80205700
C	-4.81632100	-0.90620100	-1.23076800
C	-3.83432000	-0.41495000	-0.33182000
C	-2.82703300	0.49309800	-0.74689800
C	0.91726900	0.21284500	-1.78019000
C	1.99087500	-0.62797800	-1.93760800
C	3.01115800	-0.70002100	-0.95026700
N	4.04720600	-1.55343600	-1.13009000
H	6.77408200	-2.36512200	0.33078200
H	6.09648400	-2.93855200	-1.16424200
H	3.46542100	1.63734000	2.33898700
H	4.88349800	0.78121900	2.79822000
H	1.66726800	1.61437100	1.22573100
H	-1.18841100	0.94104000	2.20454800
H	-2.91410300	-0.66189800	2.97121200
H	-4.88964200	-2.08402600	2.47586700
H	-6.56824300	-2.91391700	0.87126900
H	-6.52407100	-2.15986700	-1.49587300
H	-4.79439700	-0.57300300	-2.26276900
H	-2.79515400	0.84270700	-1.77227800
H	0.14972200	0.28244600	-2.54138500
H	2.09317400	-1.25288000	-2.81607400

Cartesian Coordinates for top-hit compound **41** optimized in the gas phase using DFT/B3LYP/6-311+g(d,p)/GD3.

N	-5.80066000	-2.51473900	0.33864400
C	-4.80955600	-1.58854000	0.18438100
N	-4.97313500	-0.44797100	0.90682700
C	-4.01662400	0.45777900	0.83189600
N	-4.17178200	1.58676500	1.57483900
C	-2.84296900	0.25311700	0.01897200
C	-1.77869500	1.16971600	-0.09108900
C	-0.71430000	0.87720000	-0.90820100
S	0.61926100	2.12101900	-1.04451300
O	0.23140000	3.23589500	-0.09077900

C	1.90810200	1.12741400	-0.21870800
C	1.92949100	1.08063700	1.19284700
C	2.88514200	0.32336700	1.82004100
C	3.85049400	-0.40796600	1.07387600
C	4.84568300	-1.20104200	1.69994200
C	5.77154000	-1.88936000	0.95142600
C	5.74799200	-1.81417200	-0.46087500
C	4.79780000	-1.05251500	-1.09906400
C	3.82649800	-0.33484700	-0.35506800
C	2.83104800	0.46083800	-0.98251400
C	-0.67125100	-0.31871800	-1.65401600
C	-1.69700200	-1.22698500	-1.54985400
C	-2.81199000	-0.97133500	-0.70633500
N	-3.80391800	-1.89333700	-0.62111000
H	-6.65674800	-2.22149900	0.77821300
H	-5.82267800	-3.28591200	-0.30764200
H	-3.60439000	2.40015400	1.41132500
H	-5.06291000	1.71742600	2.02661500
H	-1.75519000	2.10673900	0.45525100
H	1.19975300	1.65712000	1.74914900
H	2.91652400	0.27500100	2.90350700
H	4.86521500	-1.25677900	2.78320000
H	6.52612900	-2.49311900	1.44255200
H	6.48448000	-2.36065900	-1.03881600
H	4.77841600	-0.99249000	-2.18221900
H	2.81562700	0.53575400	-2.06559800
H	0.17812200	-0.52919100	-2.29531300
H	-1.68369200	-2.15856800	-2.10212800

Cartesian Coordinates for top-hit compound **68** optimized in the gas phase using DFT/B3LYP/6-311+g(d,p)/GD3.

C	-1.24404900	1.49955200	-1.05188400
C	-1.91128300	0.27297300	-0.47169100
C	-1.15371600	-0.87167500	-0.22394700
C	0.33770100	-0.93874300	-0.44273900
N	1.04190300	-0.07555000	0.49925500
C	2.42592600	0.01605300	0.50141300
C	3.05803300	0.70070000	1.59058900
N	4.34300600	0.89970500	1.70172700
C	5.16200400	0.43253700	0.71079000
C	6.55607400	0.64910200	0.81783900

C	7.41015900	0.18899100	-0.15843800
C	6.89980000	-0.50674200	-1.27902800
C	5.54818200	-0.73075900	-1.40827700
C	4.64249000	-0.26705200	-0.42002500
C	3.24260000	-0.47341800	-0.49920300
C	-1.81292900	-2.01725300	0.26851900
N	-3.10446800	-2.12957500	0.47450000
C	-3.87706200	-1.04231800	0.22519500
N	-5.20474000	-1.21836000	0.39203000
C	-5.97175700	-0.16940500	0.15681000
N	-7.32184700	-0.34453500	0.21141600
N	-5.56143900	1.09084400	-0.13330600
C	-4.26757700	1.29194200	-0.28990700
N	-3.91649800	2.58928900	-0.55409400
C	-3.30663300	0.20890600	-0.19809600
H	-0.25944500	1.26564300	-1.44925400
H	-1.84240200	1.93264800	-1.85438100
H	-1.07773100	2.26556600	-0.28569200
H	0.67319200	-1.98241700	-0.35626000
H	0.59274700	-0.62030000	-1.45986800
H	0.60517300	-0.01585700	1.40845500
H	2.43041900	1.09015800	2.39264100
H	6.91927000	1.18348100	1.68775400
H	8.47747000	0.35711000	-0.07188200
H	7.58196300	-0.86602500	-2.04150400
H	5.15917700	-1.26538800	-2.26897500
H	2.83950200	-1.01553800	-1.34607700
H	-1.21931600	-2.90436800	0.48740300
H	-7.66551600	-1.21116100	0.59105100
H	-7.90989800	0.47124000	0.23401700
H	-2.99400100	2.92103400	-0.33839500
H	-4.66980300	3.25109100	-0.43724600

Cartesian Coordinates for top-hit compound **74** optimized in the gas phase using DFT/B3LYP/6-311+g(d,p)/GD3.

C	-0.42144700	-0.97360700	-0.40581500
C	-1.53323000	-0.01504300	-0.02537000
C	-1.22277200	1.33012700	0.16411400
N	0.14221500	1.72226100	0.17757700
C	0.68590600	2.87393400	-0.32811600
O	0.04692800	3.78390400	-0.82102200

C	2.22230800	2.97256700	-0.21738200
C	2.94853400	1.68874800	0.09463600
C	3.37072500	1.38174100	1.39188300
C	3.95512600	0.15281200	1.67912800
C	4.12351300	-0.79973500	0.67600500
Cl	4.84355100	-2.33812100	1.08582200
C	3.71236900	-0.50213800	-0.62905800
Cl	3.88424500	-1.65603500	-1.93020200
C	3.13672800	0.73442600	-0.90901400
C	-2.23258600	2.29923900	0.35882800
C	-3.55255400	1.93642200	0.34023900
C	-3.92835700	0.58105600	0.18313200
N	-5.25489000	0.28624700	0.14555200
C	-5.56420300	-0.98729100	0.00189400
N	-6.88035600	-1.31658000	-0.18404900
N	-4.70129800	-2.03304300	0.02831100
C	-3.41095300	-1.76518600	0.09012300
N	-2.59467100	-2.86753100	0.18770600
C	-2.90388400	-0.41028900	0.06633600
H	-0.79055000	-1.78674100	-1.02732200
H	0.34683200	-0.45563000	-0.98244500
H	0.08426200	-1.41477500	0.46434200
H	0.79993500	1.04482100	0.53070700
H	2.55140900	3.39177000	-1.17055600
H	2.42824600	3.73095200	0.54307000
H	3.23698700	2.10550200	2.18809100
H	4.28231300	-0.08083200	2.68422900
H	2.81819200	0.94286900	-1.92356500
H	-1.94665100	3.33370500	0.48315500
H	-4.34108300	2.67067700	0.44616700
H	-7.14256600	-2.27616100	-0.02984800
H	-7.56015200	-0.60100900	0.01617400
H	-1.70649400	-2.77831800	0.65060800
H	-3.09695400	-3.72358400	0.37477900

Cartesian Coordinates for top-hit compound **85** optimized in the gas phase using DFT/B3LYP/6-311+g(d,p)/GD3.

N	-6.33743100	-0.00610700	-1.70026200
C	-5.18737900	-0.00225400	-0.97931300
N	-5.36432900	0.00081300	0.37470300
C	-4.27554300	0.00249900	1.11647100

N	-4.37683300	0.00686100	2.46115900
C	-2.96258100	0.00061600	0.51019100
N	-1.85870400	0.00131900	1.27476900
C	-0.69425700	-0.00012300	0.64826000
C	0.55083800	0.00039000	1.52239800
N	1.82549100	0.00039700	0.82696700
C	2.36502800	1.21759300	0.35590400
C	1.60030500	2.39136000	0.28194800
C	2.16227600	3.56798000	-0.20838900
C	3.48111800	3.59093500	-0.65162900
C	4.24002200	2.42343100	-0.57908200
C	3.70768700	1.24497300	-0.06530100
C	4.53840800	0.00099900	0.12333100
C	3.70862900	-1.24383400	-0.06378600
C	4.24185800	-2.42250300	-0.57616000
C	3.48382500	-3.59065300	-0.64736500
C	2.16493600	-3.56814500	-0.20423900
C	1.60207900	-2.39136600	0.28469100
C	2.36594200	-1.21695700	0.35735700
C	-0.64550900	-0.00205400	-0.76941200
N	-1.71668900	-0.00295700	-1.53608200
C	-2.92313600	-0.00187600	-0.90901700
N	-4.04533400	-0.00313100	-1.65633000
H	-7.22319600	0.00490900	-1.22650800
H	-6.28599800	0.00067400	-2.70422700
H	-3.53834000	0.00358600	3.01888100
H	-5.28648300	0.00301900	2.89084200
H	0.51559400	0.87265600	2.18020100
H	0.51599800	-0.87148500	2.18074400
H	0.56032300	2.39433300	0.57919600
H	1.55277000	4.46301700	-0.25960400
H	3.91428600	4.50207700	-1.04658000
H	5.27390100	2.42848700	-0.90979100
H	4.94552300	0.00177900	1.14627400
H	5.39706300	0.00090800	-0.55264800
H	5.27574900	-2.42717500	-0.90683500
H	3.91768200	-4.50194000	-1.04122300
H	1.55607800	-4.46367800	-0.25447700
H	0.56206300	-2.39474000	0.58182300
H	0.31652000	-0.00285200	-1.27167400

Cartesian Coordinates for top-hit compound **99** optimized in the gas phase using DFT/B3LYP/6-311+g(d,p)/GD3.

N	6.19309400	-0.52535300	-1.71708500
C	5.04819900	-0.38410400	-0.99814200
N	5.19719500	0.26958700	0.18999600
C	4.11829700	0.40338900	0.93520600
N	4.19472900	1.04530200	2.11871400
C	2.84546600	-0.12572900	0.50112200
N	1.75145900	0.01132900	1.27106200
C	0.62493200	-0.49192100	0.80049200
C	-0.62205600	-0.36967000	1.65412000
N	-1.81063300	-0.05791900	0.88803500
C	-2.07114000	1.18324600	0.31682800
C	-1.32420100	2.35939900	0.39040900
C	-1.81863600	3.47774700	-0.27538100
C	-3.02119500	3.42600300	-0.99859700
C	-3.75552000	2.24679800	-1.07416600
C	-3.28178000	1.10951600	-0.41572700
C	-3.75926600	-0.25079700	-0.29124300
C	-4.87210600	-0.93210200	-0.78934700
C	-5.03106500	-2.28109400	-0.48961300
C	-4.08606900	-2.95376500	0.30079800
C	-2.96831600	-2.29848700	0.81078400
C	-2.81718900	-0.94312300	0.51368300
C	0.59698300	-1.13117700	-0.46768400
N	1.65588300	-1.27779100	-1.23354900
C	2.82966000	-0.77327400	-0.76179100
N	3.93931800	-0.90186100	-1.51451300
H	7.01557100	-0.02599400	-1.42550500
H	6.12684600	-0.88493000	-2.65439700
H	3.37172200	1.11926100	2.69418700
H	5.08411500	1.38928300	2.43956600
H	-0.80078500	-1.30069700	2.19762300
H	-0.45148300	0.40584800	2.40531700
H	-0.38784300	2.40142100	0.93352600
H	-1.25927700	4.40563600	-0.23844400
H	-3.37569000	4.31486700	-1.50714500
H	-4.68053300	2.20889200	-1.63861400
H	-5.59945400	-0.41505800	-1.40525300
H	-5.88863100	-2.82183600	-0.87218700
H	-4.22490000	-4.00684600	0.51727300

H	-2.24401900	-2.83452900	1.41249500
H	-0.34293400	-1.52350200	-0.84755700

Cartesian Coordinates for top-hit compound **180** optimized in the gas phase using DFT/B3LYP/6-311+g(d,p)/GD3.

N	-6.83292900	2.95109100	-0.85385400
C	-7.59012900	1.86353900	-0.53114100
C	-6.95604100	0.65744600	-0.18479100
N	-5.55891000	0.65925300	-0.25633700
N	-4.98509300	-0.40227600	0.10187200
C	-3.57870200	-0.40371700	0.01422000
C	-2.95243000	-1.59278500	0.40809800
C	-1.57008500	-1.71021100	0.36338400
C	-0.76997700	-0.64349600	-0.07381100
C	0.70250700	-0.76467900	-0.12474100
C	1.31658800	-1.96476900	-0.51464900
C	2.69950600	-2.07474900	-0.56119500
C	3.51168300	-0.98760300	-0.21826900
N	4.90558900	-1.20141900	-0.29806200
N	5.62438800	-0.20246500	0.00198300
C	7.00581100	-0.33381200	-0.05052100
C	7.69734000	0.84804800	0.29996000
C	9.06212000	0.91059600	0.31486400
S	9.76989700	2.46826000	0.80028700
O	8.70705300	3.41752100	1.08987900
O	10.86087900	2.29906800	1.75621700
O	10.46900100	2.87427200	-0.64632000
C	9.86673000	-0.22636200	-0.03813500
C	11.28195100	-0.21386700	-0.04317000
C	12.00367500	-1.33105400	-0.40011900
C	11.34563600	-2.51653900	-0.76938300
C	9.96985700	-2.55899100	-0.76990900
C	9.19340300	-1.43225100	-0.40792400
C	7.74479900	-1.49300800	-0.41021200
N	7.09362200	-2.62237400	-0.74685700
C	2.91155300	0.21907500	0.17590300
C	1.53130000	0.32099000	0.21788200
C	-1.41130300	0.54828100	-0.46551200
C	-2.78925800	0.67191100	-0.42625400
C	-7.72224400	-0.47284600	0.17678800
C	-9.08793000	-0.41841700	0.18305100

S	-9.94385900	-1.93353700	0.59573900
O	-10.98046000	-2.21128600	-0.37966300
O	-8.96297500	-2.94036100	0.97824500
O	-10.77428700	-1.51003500	1.96291900
C	-9.79967900	0.78383600	-0.15465900
C	-11.21104900	0.88921000	-0.14802400
C	-11.83611000	2.07883400	-0.45230800
C	-11.08197300	3.22246700	-0.76742400
C	-9.70721700	3.14998100	-0.78506600
C	-9.03003400	1.94111100	-0.49560000
H	-7.22806100	3.69257500	-1.40573300
H	-5.84456700	2.77668100	-0.96542900
H	-3.57198800	-2.41111500	0.75556100
H	-1.10294200	-2.62904700	0.69750000
H	0.70499600	-2.80898600	-0.81025100
H	3.16860100	-3.00050700	-0.87442700
H	7.10167400	1.71305000	0.56065500
H	11.13131500	3.56106800	-0.47162500
H	11.80535000	0.68298800	0.25142800
H	13.08681500	-1.29336700	-0.39274400
H	11.91652800	-3.39329400	-1.05099900
H	9.49189600	-3.48680400	-1.06009000
H	6.07488100	-2.58882900	-0.72456200
H	7.58347700	-3.46407700	-0.99215200
H	3.53929900	1.05531300	0.45321500
H	1.08098100	1.24943500	0.54976300
H	-0.81519700	1.37622700	-0.83176900
H	-3.26842800	1.58891000	-0.74192000
H	-7.20003100	-1.38517700	0.43123200
H	-10.19858100	-1.65817400	2.72787300
H	-11.81071600	0.02364200	0.09175400
H	-12.91855600	2.13131800	-0.44078300
H	-11.57953400	4.15930600	-0.98858300
H	-9.14597200	4.05017100	-1.00377200

Cartesian Coordinates for reference compound **MTX** optimized in the gas phase using DFT/B3LYP/6-311+g(d,p)/GD3.

C	-2.37227900	4.28902300	-0.15553000
N	-2.05592400	2.98988600	0.42043600
C	-3.07000600	2.30563900	1.20003700

C	-3.87358100	1.25624600	0.44647200
C	-3.79583000	1.09305200	-0.95958800
N	-4.50647100	0.21211100	-1.63376200
C	-5.35124900	-0.57508400	-0.91620400
N	-6.10051800	-1.48796400	-1.56646400
C	-6.89760600	-2.22647600	-0.80416800
N	-7.69135300	-3.13531800	-1.43044500
N	-7.04231100	-2.18334700	0.55270400
C	-6.31633300	-1.29544000	1.20086000
N	-6.40869600	-1.20025200	2.54257000
C	-5.41269600	-0.41477100	0.49348600
N	-4.68120000	0.49118900	1.16176500
C	-0.84093700	2.38570900	0.14984400
C	-0.56631300	1.06091900	0.55917000
C	0.66179000	0.47434000	0.29435100
C	1.66872600	1.16258800	-0.39306700
C	1.38923300	2.46429000	-0.82343500
C	0.17164000	3.06957000	-0.56090800
C	3.00631500	0.59083400	-0.72201600
O	3.74913400	1.09744700	-1.55955700
N	3.38451900	-0.53398400	-0.04062400
C	4.64987100	-1.18005000	-0.32264000
C	5.84179800	-0.43090200	0.33148300
C	7.19514200	-0.76640300	-0.29062300
C	8.33236900	-0.03774000	0.38173900
O	9.50092400	-0.23302100	-0.28164700
O	8.25937900	0.62858100	1.38371500
C	4.55979500	-2.61216100	0.17439200
O	5.54658700	-3.38510000	-0.33046600
O	3.73464500	-3.02735400	0.95056300
H	-3.40040500	4.54815700	0.09281500
H	-1.71867500	5.07756700	0.23527900
H	-2.28359700	4.28017100	-1.24809000
H	-2.62140900	1.83437600	2.08039000
H	-3.76384200	3.05467400	1.59030000
H	-3.11122200	1.71107600	-1.53229600
H	-8.19678700	-3.80395900	-0.87519500
H	-7.54112300	-3.30475800	-2.41073200
H	-5.85429900	-0.51395300	3.02808600
H	-7.04220000	-1.80256600	3.04070900
H	-1.31983500	0.47163100	1.06251000

H	0.80431300	-0.55479400	0.60601100
H	2.15474500	2.99822300	-1.37316800
H	0.01221600	4.08163300	-0.90556400
H	2.82550600	-0.92009500	0.70416000
H	4.79887800	-1.19101500	-1.40602200
H	5.64252000	0.63241900	0.19616100
H	5.85429900	-0.61941300	1.40823700
H	7.41854500	-1.83560300	-0.23819900
H	7.21006400	-0.51077400	-1.35484300
H	10.19029400	0.24788700	0.20041100
H	5.45122600	-4.27065100	0.05300300

Cartesian Coordinates for reference compound **PTX** optimized in the gas phase using DFT/B3LYP/6-311+g(d,p)/GD3.

N	2.03385300	0.99217300	-0.01949600
N	2.82634400	0.03355100	-0.20139400
C	4.18526400	0.31860100	0.00945500
C	4.69964800	1.58973400	0.39072000
C	6.04534700	1.76872000	0.56299000
C	6.96200700	0.69497400	0.36984800
N	8.28487000	0.91606700	0.53853800
C	9.07948500	-0.12845700	0.33671600
N	10.42040400	0.07167600	0.45404300
N	8.71101500	-1.39446000	0.01009100
C	7.42197200	-1.62429600	-0.15653400
N	7.06254300	-2.88872300	-0.50659900
C	6.43937200	-0.57980900	-0.00052200
C	5.05753100	-0.74261500	-0.18169500
C	0.66795700	0.69169100	-0.23804900
C	-0.22385000	1.74567400	-0.01104500
C	-1.58984700	1.56108300	-0.18964500
C	-2.08526600	0.31948700	-0.60345000
C	-1.18482000	-0.72799300	-0.85019400
C	0.17587300	-0.55354100	-0.66436900
C	-3.54217800	0.04695200	-0.83322300
O	-3.92639100	-0.86580900	-1.55685300
N	-4.42541900	0.86474200	-0.18975500
C	-5.85493600	0.70524600	-0.37238900
C	-6.41307300	-0.48376600	0.45370400
C	-7.77616400	-0.97929400	-0.02339100

C	-8.28782500	-2.13127900	0.80647000
O	-9.43581800	-2.63676100	0.28808900
O	-7.78054900	-2.56698900	1.80931500
C	-6.51779200	2.01448600	0.02063700
O	-7.79236000	2.08657500	-0.41940300
O	-5.99664700	2.88585000	0.67197800
H	4.00358900	2.40554900	0.53536100
H	6.45528200	2.73044200	0.84710400
H	11.03060300	-0.72758700	0.45666900
H	10.74080200	0.94780200	0.83059900
H	6.10884200	-3.19673700	-0.43519500
H	7.78541800	-3.59055600	-0.48336600
H	4.62481800	-1.68943200	-0.48473600
H	0.17519500	2.70396600	0.29918700
H	-2.25625500	2.40193900	-0.03370200
H	-1.58200200	-1.67573700	-1.19235300
H	0.87184000	-1.36150300	-0.84590700
H	-4.11913500	1.56012200	0.47384500
H	-6.04767700	0.51522700	-1.43190000
H	-5.68736500	-1.29147700	0.35452400
H	-6.44873100	-0.21556500	1.51287900
H	-8.53488900	-0.19271000	0.00694700
H	-7.72981700	-1.30806000	-1.06653300
H	-9.71854000	-3.36283200	0.86417600
H	-8.16535000	2.92546900	-0.10683100

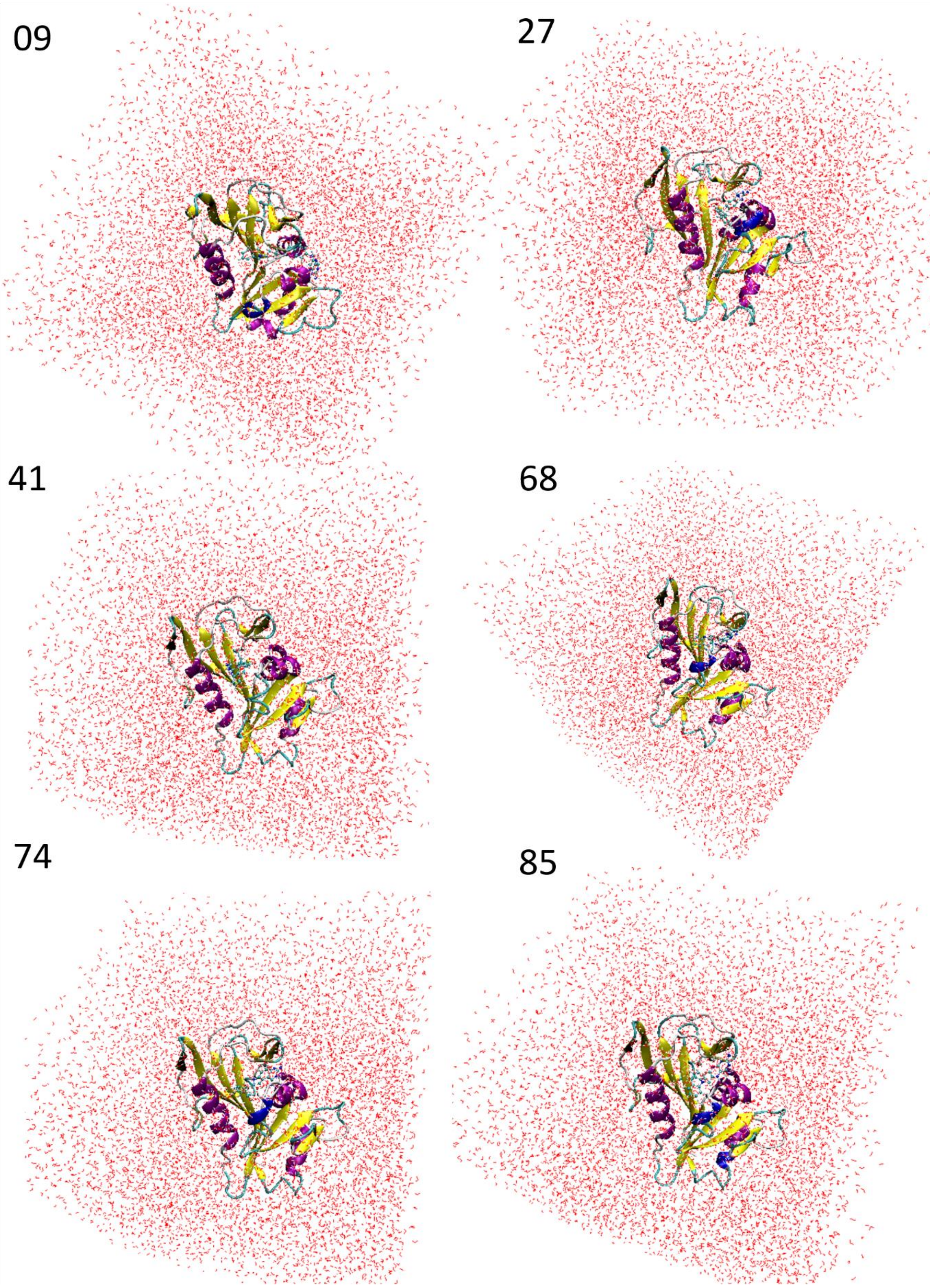
09
27
41
68
74
85

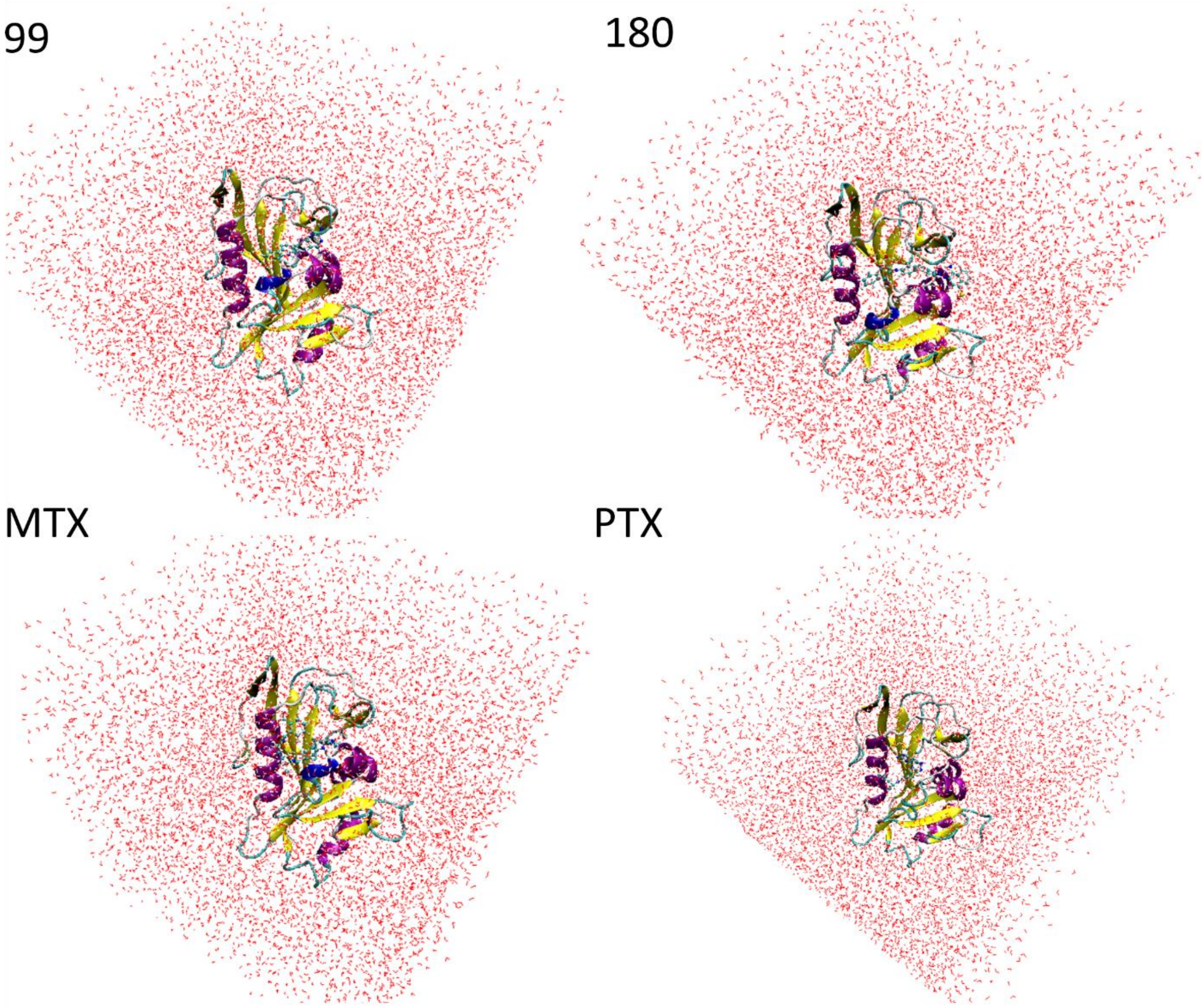

Figure S18: The snapshots of investigated ligands- and reference-protein complexes at 0 ns.

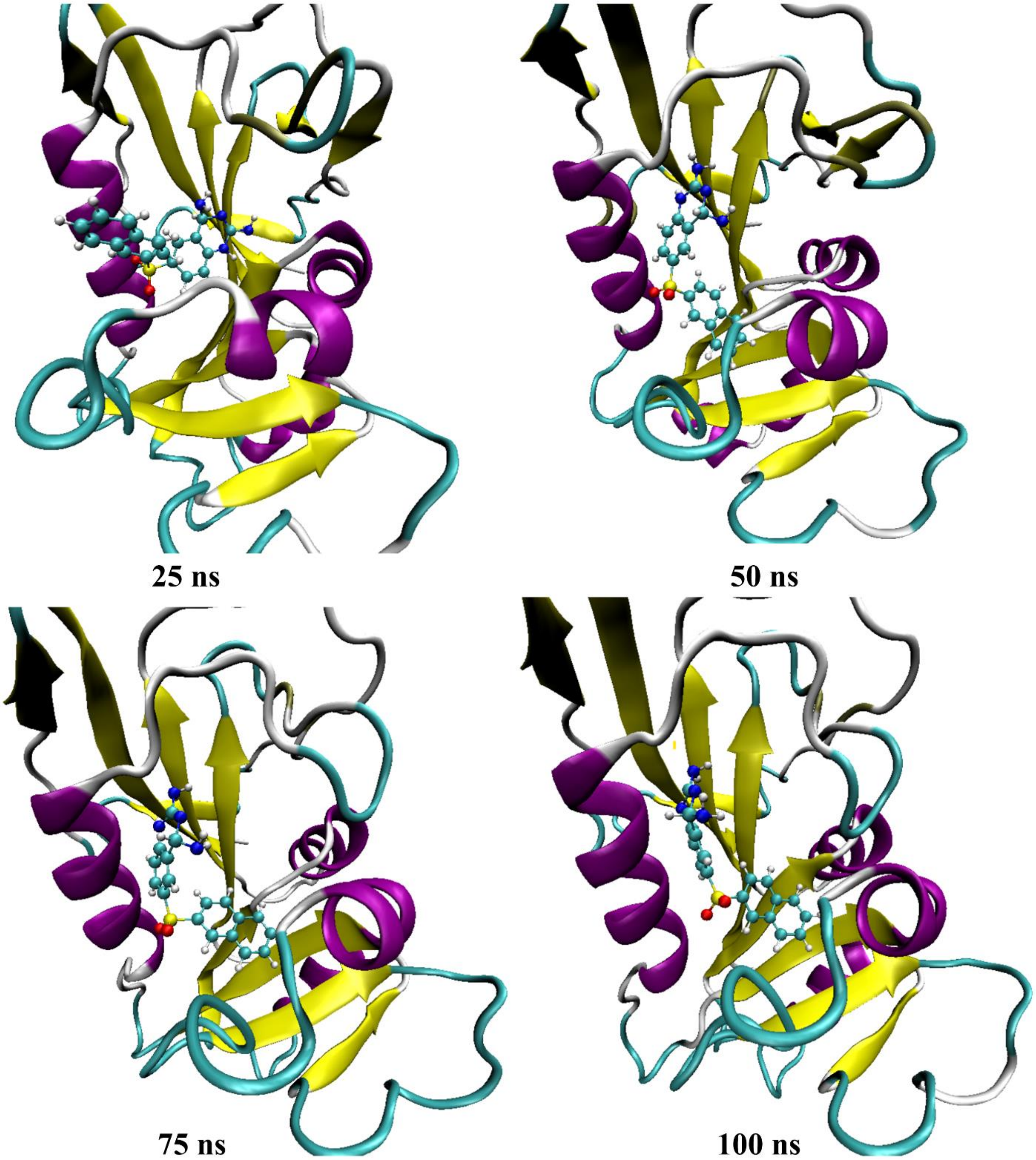

Figure S19: The snapshots of ligand-27 protein complex at 25, 50, 75 and 100 ns.

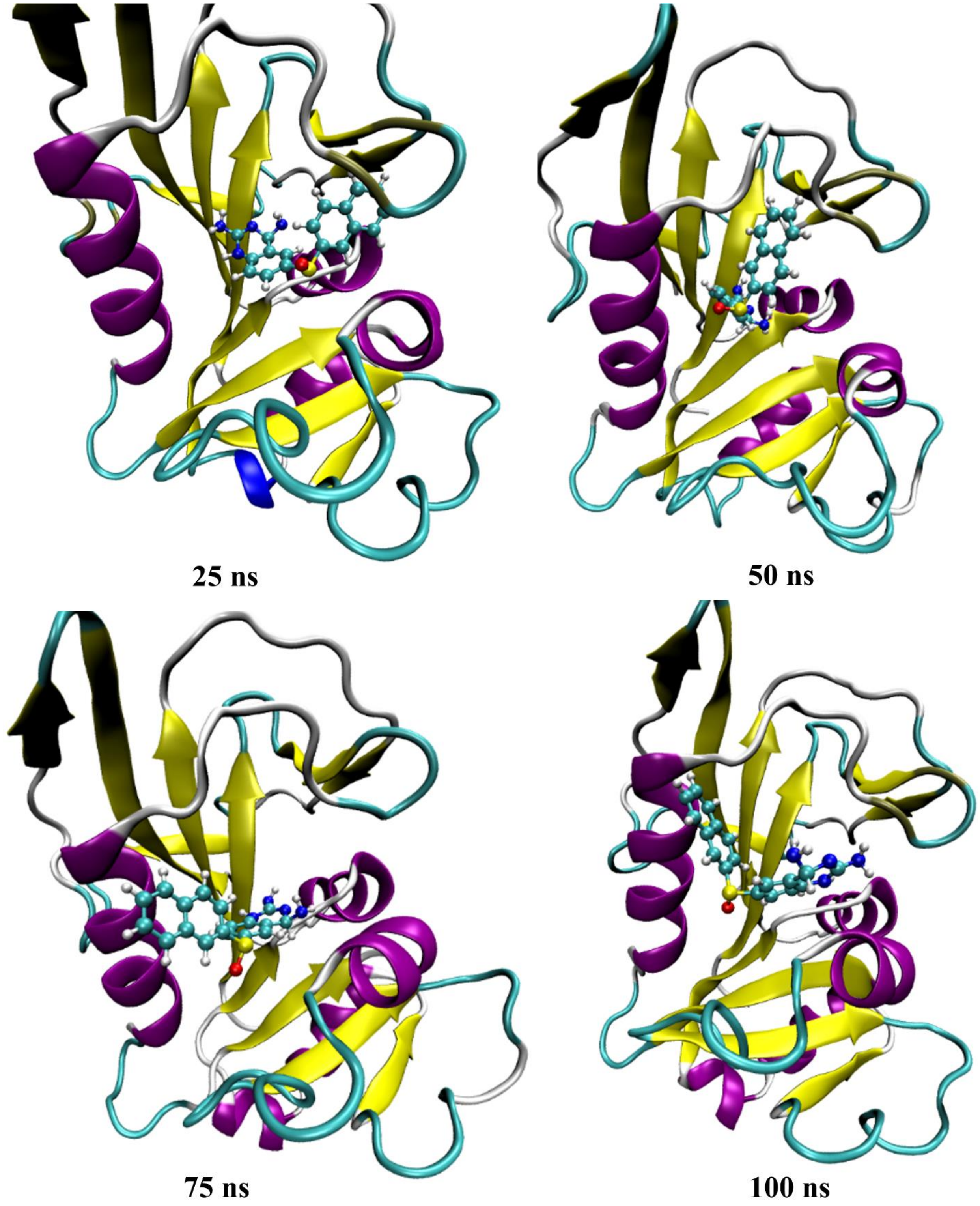

Figure S20: The snapshots of ligand-41 protein complex at 25, 50, 75 and 100 ns.

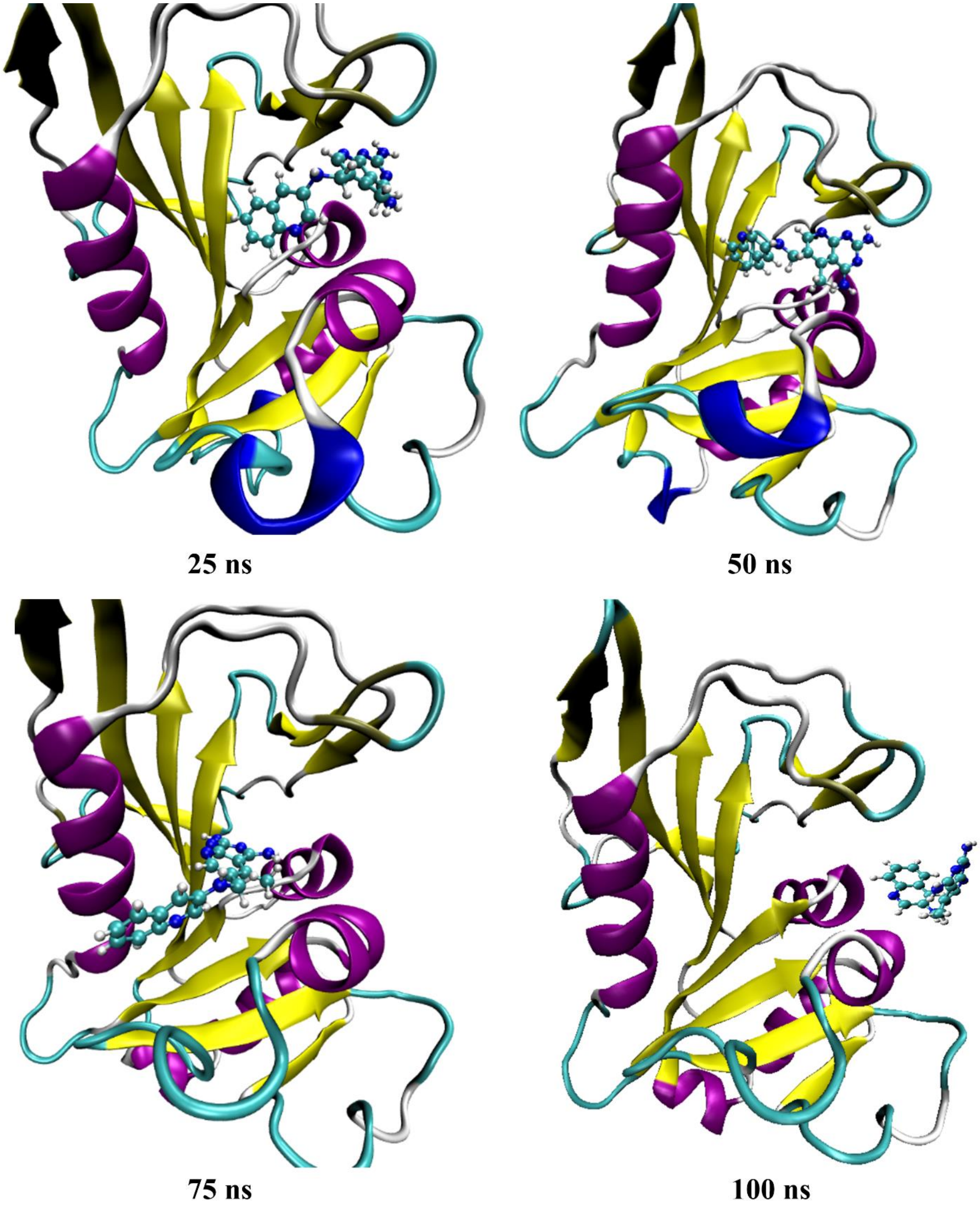

Figure S21: The snapshots of ligand-68 protein complex at 25, 50, 75 and 100 ns.

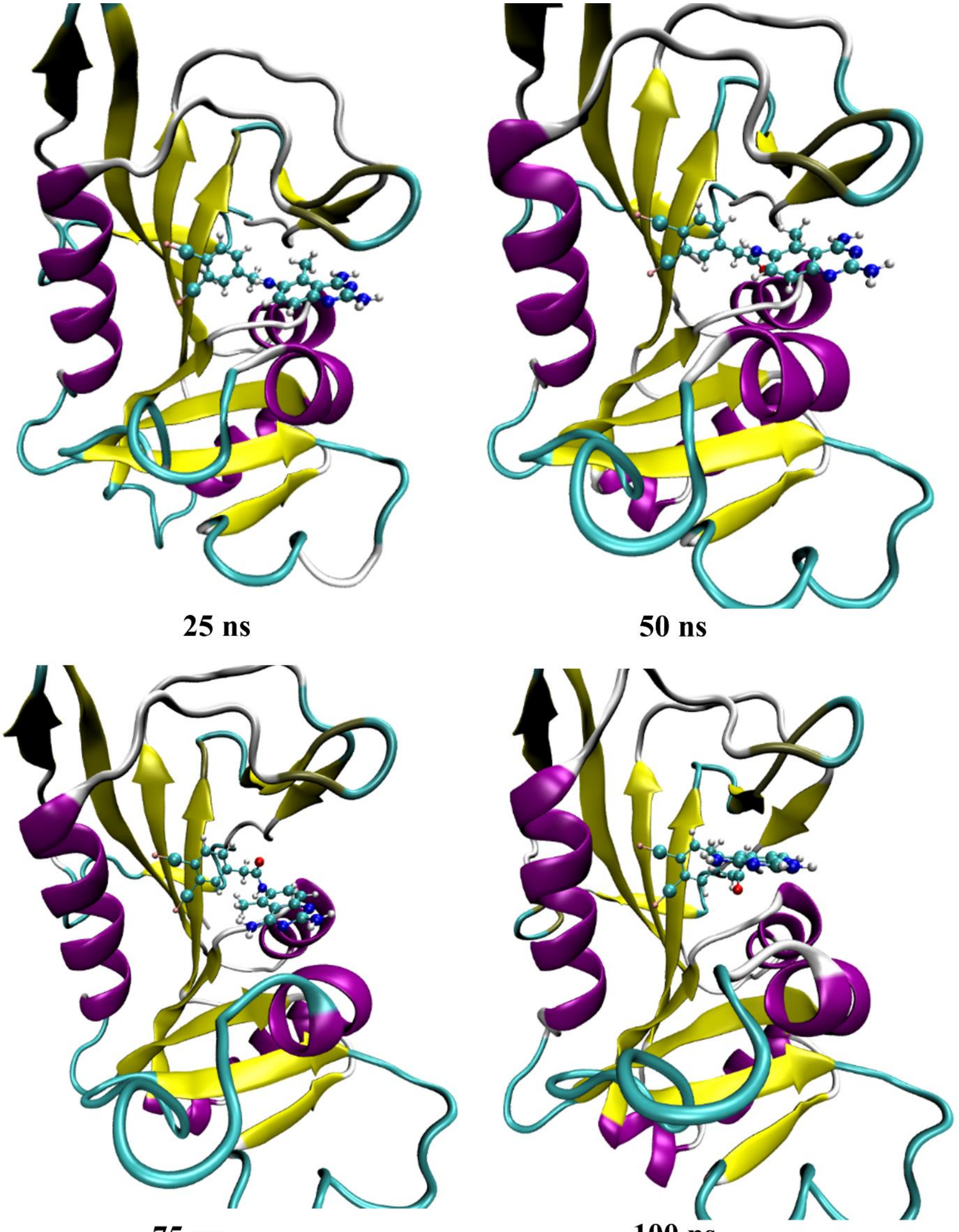

Figure S22: The snapshots of ligand-74 protein complex at 25, 50, 75 and 100 ns.

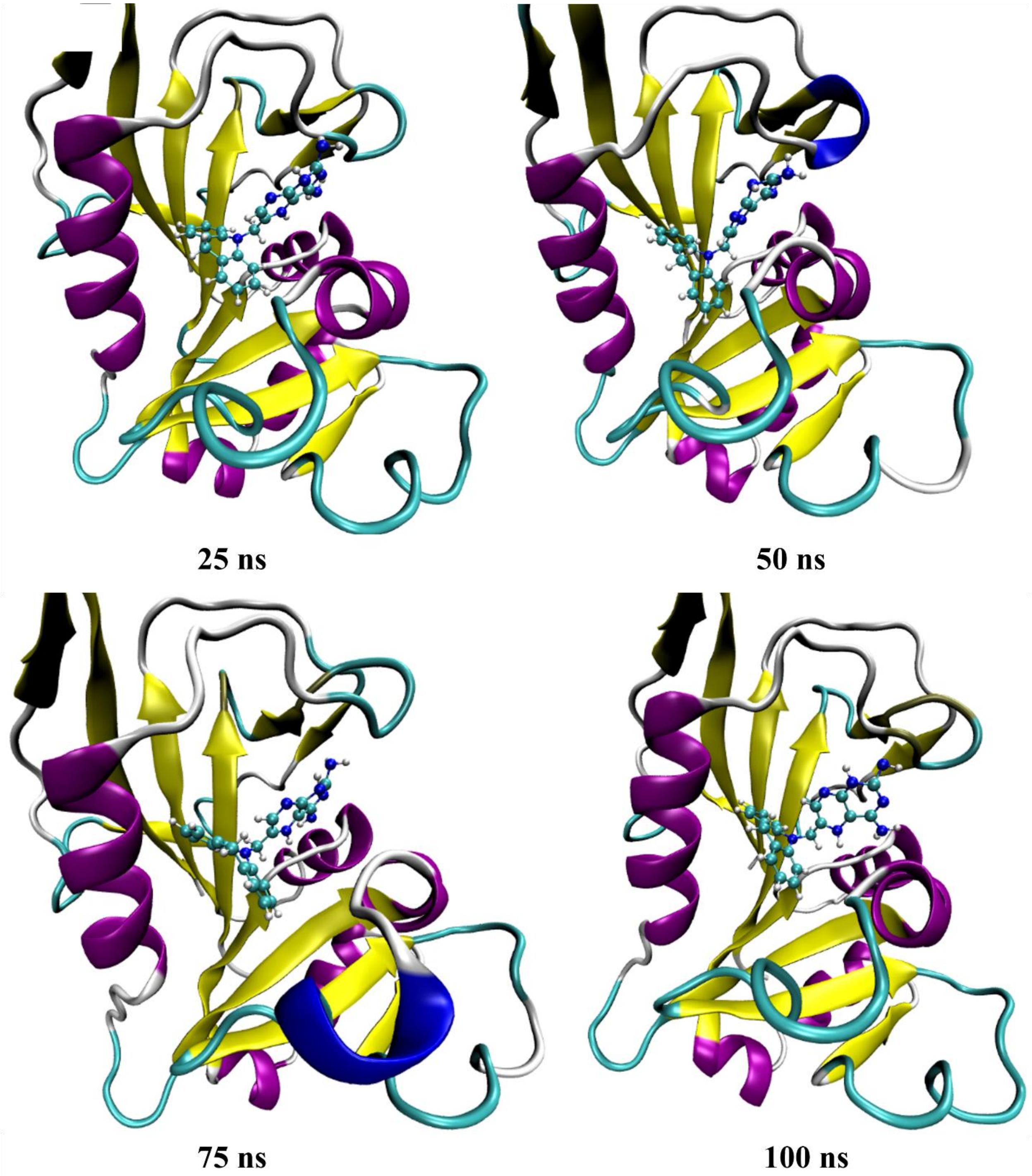

Figure S23: The snapshots of ligand-85 protein complex at 25, 50, 75 and 100 ns.

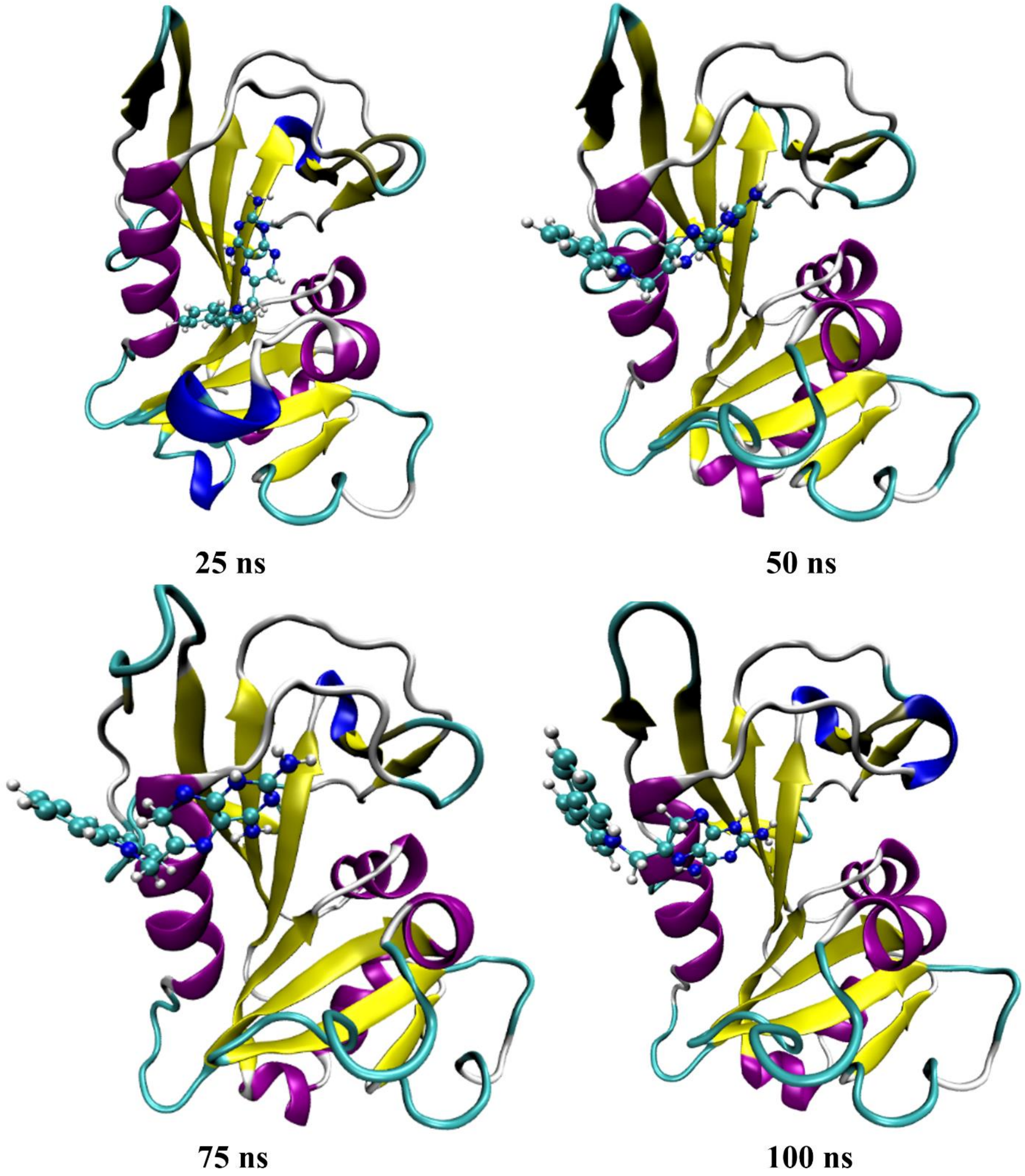

Figure S24: The snapshots of ligand-99 protein complex at 25, 50, 75 and 100 ns.

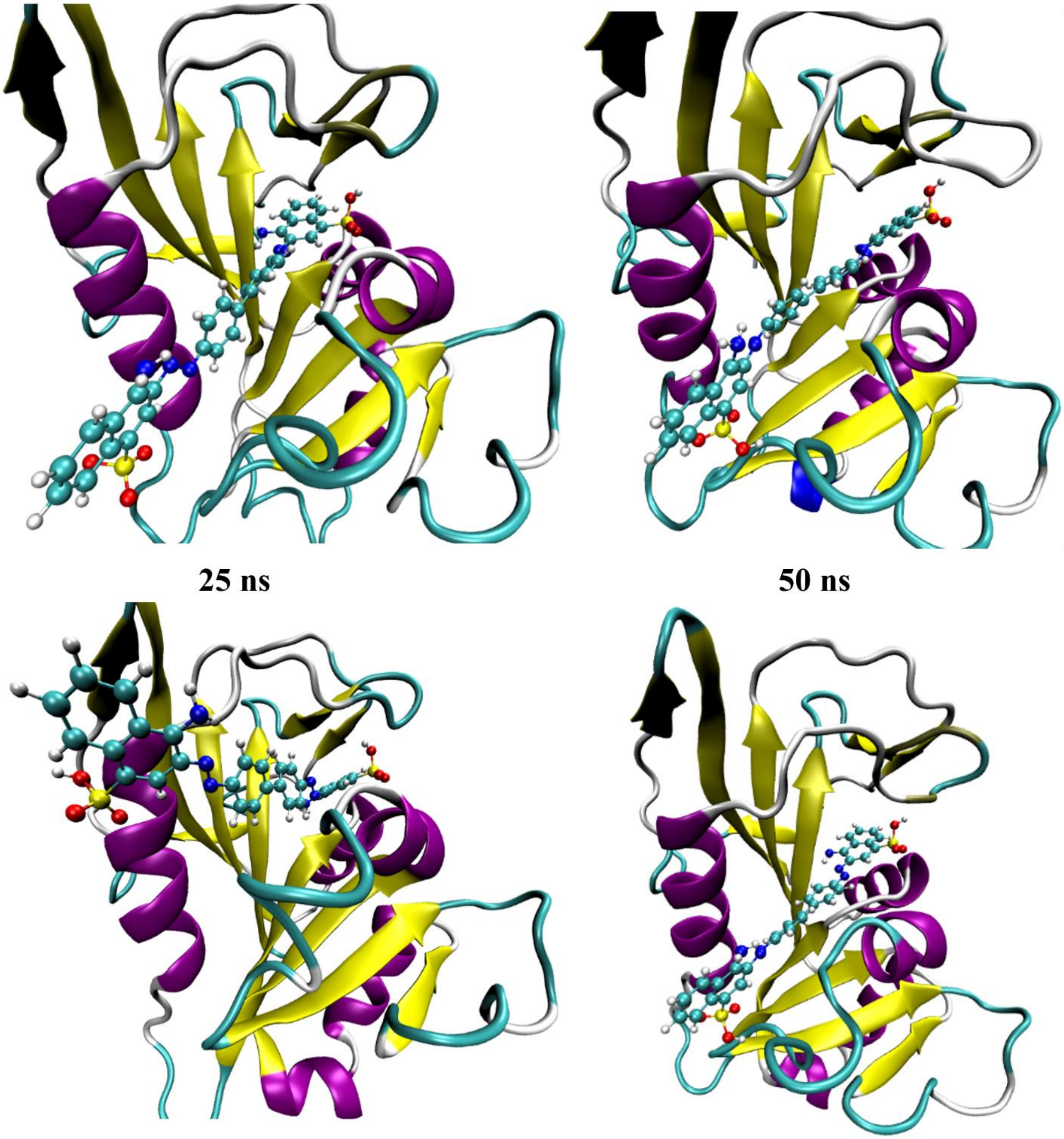

Figure S25: The snapshots of ligand-185 protein complex at 25, 50, 75 and 100 ns.

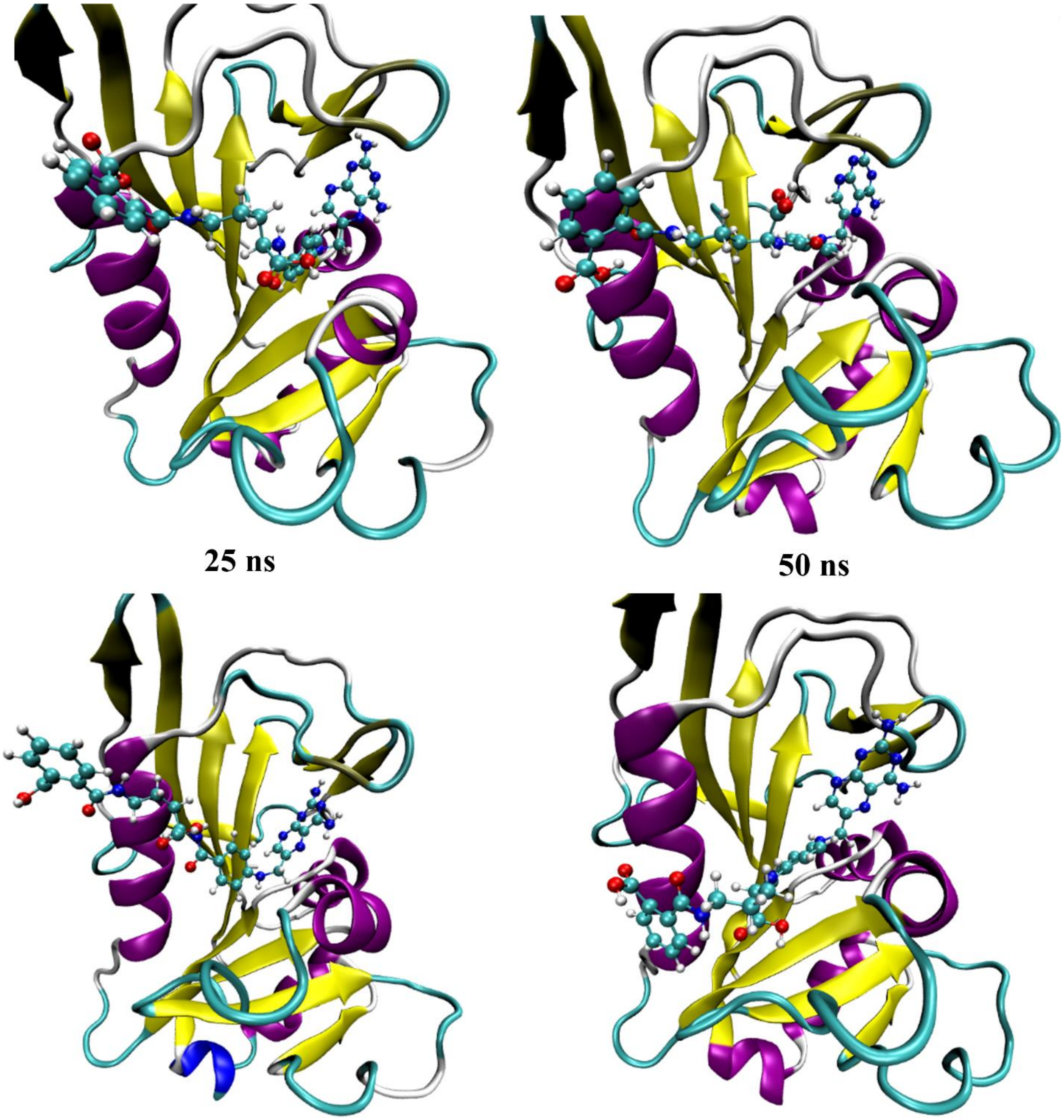

Figure S26: The snapshots of ligand-185 protein complex at 25, 50, 75 and 100 ns.

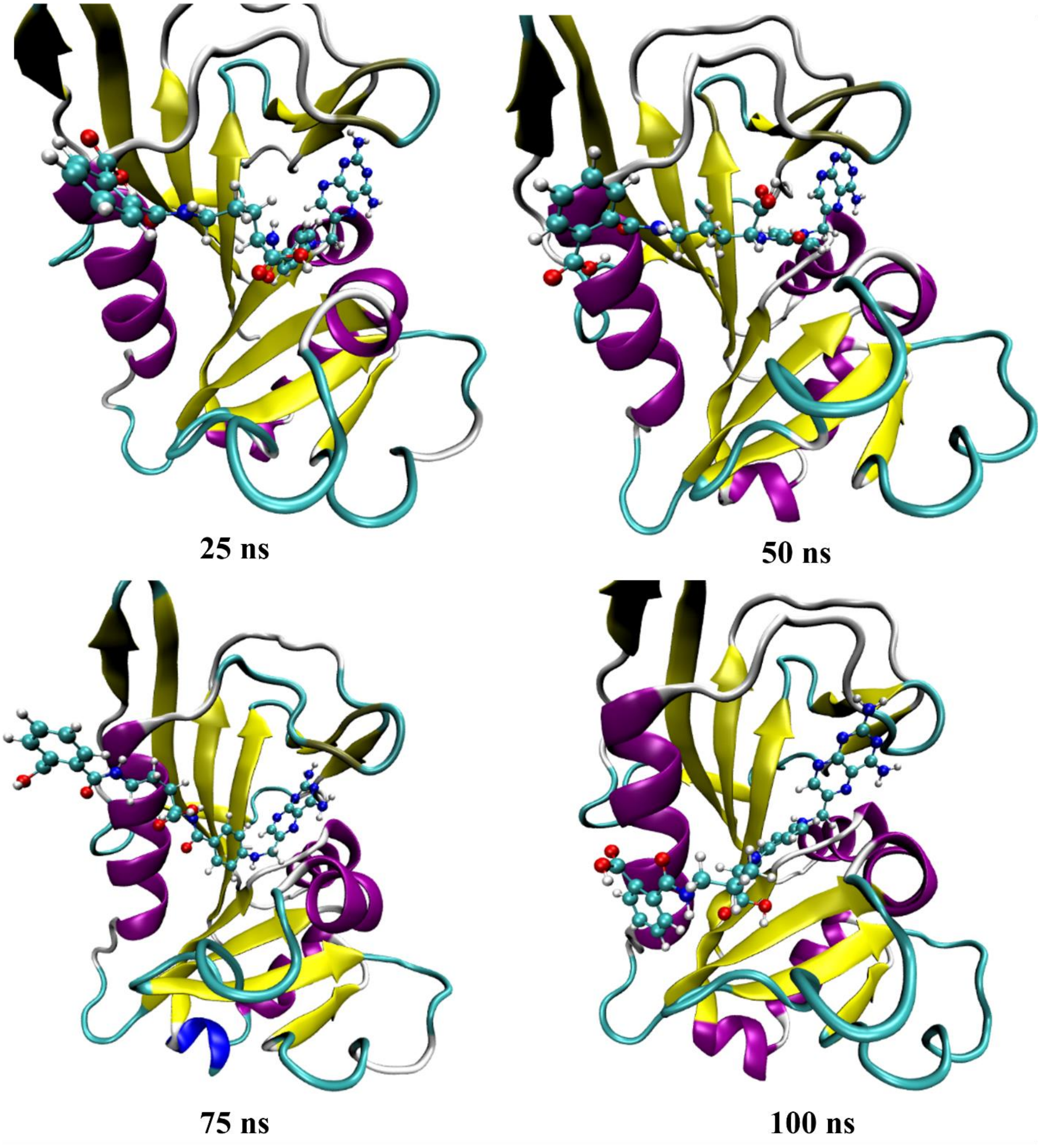

Figure S27: The snapshots of MTX-protein complex at 25, 50, 75 and 100 ns.

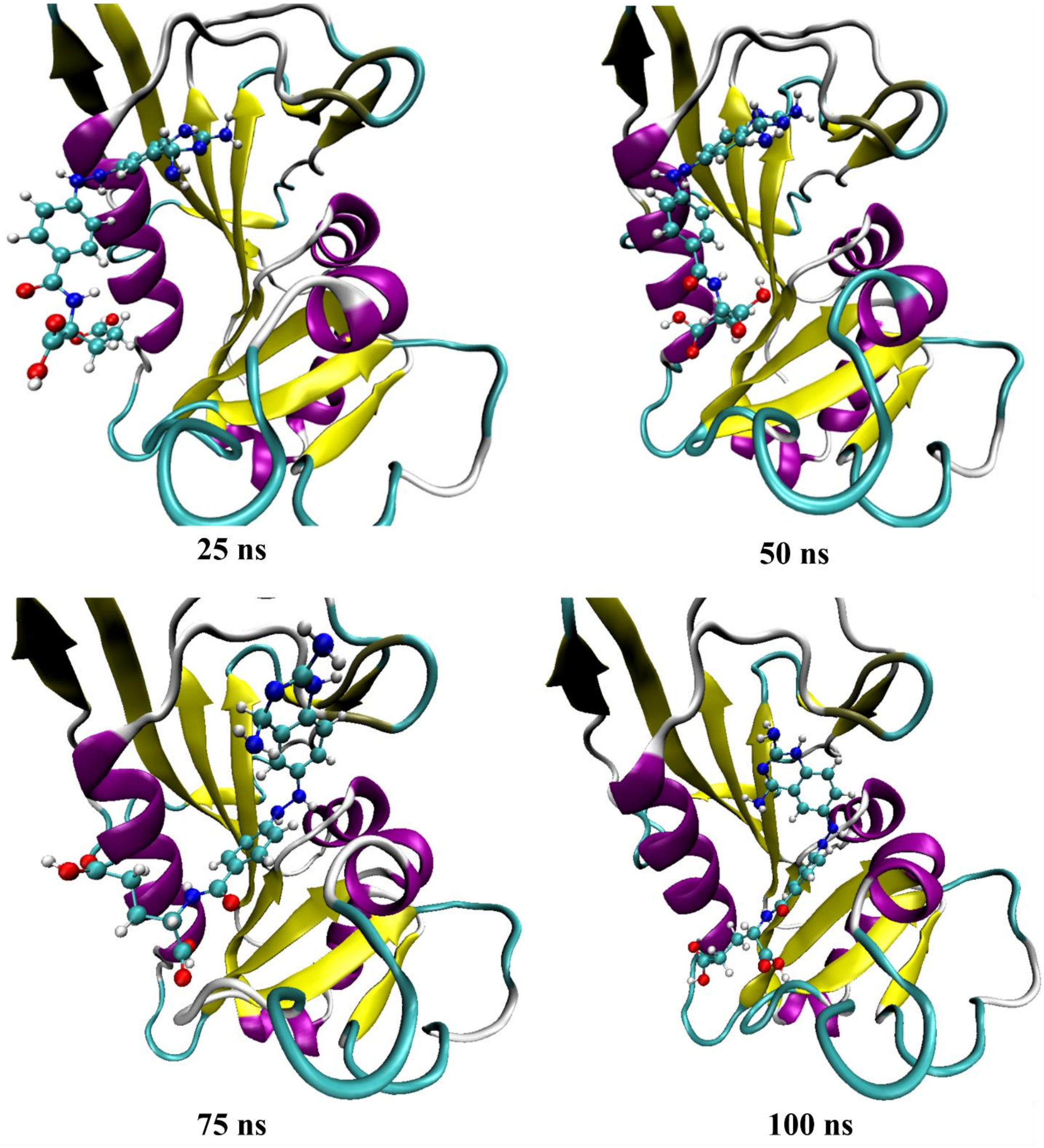

Figure S28: The snapshots of PTX-protein complex at 25, 50, 75 and 100 ns.
